# Wild parrots exhibit age-dependent conformity when learning about novel food

**DOI:** 10.1371/journal.pbio.3003741

**Published:** 2026-04-30

**Authors:** Julia Penndorf, Brendan J. Barrett, Sonja Wild, John M. Martin, Lucy M. Aplin

**Affiliations:** 1 Division of Ecology and Evolution, Research School of Biology, The Australian National University, Canberra, Australia; 2 Cognitive and Cultural Ecology Research Group, Department of Migration, Max Planck Institute of Animal Behavior, Radolfzell, Germany; 3 Department of Biology, University of Konstanz, Konstanz, Germany; 4 Centre for the Advanced Study of Collective Behaviour, University of Konstanz, Konstanz, Germany; 5 Department for the Ecology of Animal Societies, Max Planck Institute of Animal Behavior, Konstanz, Germany; 6 Department of Human Behavior, Ecology, and Culture, Max Planck Institute of Evolutionary Anthropology, Leipzig, Germany; 7 Department of Environmental Science and Policy, University of California Davis, Davis, Davis, California, United States of America; 8 School of Life and Environmental Sciences, The University of Sydney, Camperdown, New South Wales, Australia; 9 Department of Evolutionary Biology and Environmental Studies, University of Zurich, Zurich, Switzerland; Queen Mary University of London, UNITED KINGDOM OF GREAT BRITAIN AND NORTHERN IRELAND

## Abstract

There is extensive evidence that the spread of innovation via social learning can facilitate uptake of new foraging behaviours in populations. In comparison, social learning about novel food types has received comparatively little attention. Yet the adoption of novel food is vital to persistence in, or colonisation of, novel environments. Here, we present a novel food (almonds in the shell, coloured either blue or red) in a two-option and control cultural diffusion experiment to five neighbouring roosts of 705 individually-marked sulphur-crested cockatoos (*Cacatua galerita*) living in a highly urbanised environment. From 4 initially trained individuals, a total of 349 individuals across all roosts learned to feed on the novel food within 10 days of first exposure. Using network-based diffusion-analysis (*N* = 214 learners out of 322 individuals with available social information), we demonstrated that this spread occurred almost exclusively through social learning, with information spreading through social network ties. Second, using experience-weighted attraction models, we described age-differences in social learning strategies, with juveniles, but not adults, exhibiting a conformist bias to prefer the most frequently chosen food colour. Third, when analysing 539 opening techniques of the novel food by 147 individuals across the five roosts, we found that opening techniques were more similar between roost communities when the distance between sites was small, or the degree of movement between sites was high. In addition, when focusing on a subset for which social association data were available (273 openings by 78 individuals), techniques tended to be more similar between close associates. Taken together, our study suggests that the adoption of novel food in urban-living sulphur-crested cockatoos is facilitated by social transmission of knowledge through networks, with food choice further influenced in juveniles by a conformist learning bias. Social networks influenced both food choice and acquisition of foraging techniques within and between roosting communities, leading to differences at surprisingly local scales. The utilisation of new food resources is a fundamental component of adaptive behavioural responses to novel environments. Our study demonstrates how cognitive and social influences can be vital determinants of this adaptive flexibility.

## Abstract 2

Although adoption of novel food is vital to colonisation of novel environments, social learning about novel food types is not well studied. This study of urban sulphur crested cockatoos shows that they rapidly adopted a novel food through social learning, with juveniles (but not adults) showing conformist preferences.

## 1 Introduction

Learning about food resources is a major challenge for animals living in urban environments [1–3]. Urban resources are often highly novel, consisting of human-derived waste or provisioned items, exotic garden plants and street trees, and invasive plants and animals. While expanding one’s diet to encompass these items can be vital for animals colonising, or attempting to persist in, landscapes affected by human-induced rapid environmental change [1,4], consuming novel food items or accessing novel food resources carries major risks, such as exposure to parasites, or the ingestion of toxic food items [2,5–7]. Therefore, avoidance of novel food sources—or dietary wariness—should be favoured [8,9]. This food neophobia and dietary conservatism can potentially be overcome by accessing social information from conspecifics about the novel food items (social information hypothesis, [10]). Numerous examples suggest that the presence of knowledgeable conspecifics can increase the exploration and subsequent adoption of novel food items (reviewed by [10,11]). For example, wild jackdaws (*Corvus monedula*) are more likely to consume a novel food item after observing a conspecific doing so [12], and when presented with two novel food items, wild rooks (*Corvus frugilegus*) chose the same food as a knowledgeable demonstrator [13].

Such social learning, or “*learning through the observation of, or interaction with, other individuals or their product*” [14], is a vital form of behavioural plasticity [15,16]. By relying on the previous experience of others, individuals can avoid potential risks of individual learning (i.e., predation risk, ingestion of toxic food items, time for learning) [17]. Yet social learning is, per se, not inherently adaptive [18,19], as social information can be outdated, unsuited, or maladaptive [19,20]. Theory suggests that social learning is rendered adaptive when integrated with individual learning [21,22]. Modelling also suggests that individuals should not copy indiscriminately, but rather should be strategic in whom they learn from, and under which circumstances [23,24]; these are often referred to as “*who, what and when to copy*”. For example, a series of now classic lab studies have demonstrated that Norway rats (*Rattus norvegicus*) acquire long-lasting food preferences by sniffing the breath of knowledgeable demonstrators [11,25–27]. The study [25] did not find any evidence that individuals relied more on social learning after prior experience with toxic foods, but individuals used social learning more when uncertain about the safety of unfamiliar foods. In addition, individuals were more likely to copy when dissatisfied or when current options were unproductive. However, they did not exhibit any preferences for copying particular individuals [25], and the potential for frequency-dependent biases was not fully explored (but see [28]).

Yet, while social learning strategies in relation to novel food have been well studied in the laboratory, they have received relatively little empirical attention in wild studies, with most work focusing either on the ontogeny of dietary knowledge [29–32]—but see [33], or the acquisition of new foraging skills and techniques [34,35]. This is surprising, as, given the risks inherent in the consumption of novel food, evolved social learning strategies that minimize these risk—such as following maternal food choices or following local norms [33]—should be highly advantageous. Overcoming dietary wariness to expanding one’s foraging repertoire to include anthropogenic resources may be of particular importance in novel environments [2,36], understanding how social learning and transmission of dietary knowledge influences food adoption has extensive implications for predicting adaptive behavioural responses to anthropogenic change [37,38]. Furthermore, the transmission of behaviours through social learning could potentially lead to the emergence of adaptive cultures [39], where individuals of the same social group share specific dietary knowledge that persists over generations [40,41].

Here, we explore the importance of social learning about novel food in a wild, urban population of sulphur-crested (SC-) cockatoos (*Cacatua galerita*). These large parrots occur across Eastern Australia and New Guinea [42], and are highly social, with each communally-roosting community sharing a foraging range around a single sleeping site [43]. SC-cockatoos are generalist foragers that eat a variety of foods, including roots and bulbs, grass shoots and seeds, nuts, fruits, and wood-boring larvae. They are successful urban adaptors throughout their native range, and in urban areas they eat a variety of native and nonnative plants from eucalyptus gum-nuts and native figs to cape-lilac seeds and oak-tree acorns [44]. In some cities, they are also regularly provisioned by residents [45,46], with provisioned food largely consisting of seeds (e.g., sunflower), and nuts, including almonds (*personal observation*). Whether SC-cockatoos use social learning when incorporating novel food items into their diet has to date not been explored. Yet, SC-cockatoos are capable of socially learning foraging techniques; in a recent study, urban SC-cockatoos were observed to open household-bins to forage on their contents [47], with this innovation spreading via social learning from 2 to 42 suburbs over several years. However, these observations were largely at the population level, and we are lacking information on social learning at the individual level, including whether we observe heterogeneity in how and what individuals learn.

The aims of this study were therefore 2-fold. In an open diffusion experiment, we presented urban-dwelling SC-cockatoos with a novel food resource (unshelled almonds, considered novel to the birds due to their artificial colouring in the experiment) to explore (i) whether knowledge about this novel food resource will spread through social learning, and, if so, (ii) whether SC-cockatoos use social learning strategies by preferentially learning from specific individuals (e.g., adults, males or individuals of the same roost) or showing a bias towards the more frequent behaviours (conformity). To do so, we individually paint-marked SC-cockatoos at five roosting communities in central Sydney and measured their social associations [43] using presence scans [48]. We then trained two individuals at experimental roosts to eat one of two novel food items (unshelled almonds, artificially coloured red or blue—two colours that are easily distinguishable for tetra-chromatic birds [49], such as SC-cockatoos) and presented these food items at five roosting communities over a 10 day period, tracking uptake over space and time and across the social network. Given the inherent risks of consuming novel foods, we hypothesised that the adoption of the novel food should primarily occur via social learning, with information that the food items are palatable spreading via social network ties within and between roosts. Second, we hypothesised that learning should not be unbiased, but that individuals should preferentially copy those individuals or groups with more reliable local information. We had no clear predictions as to the form of this bias, but expected to observe age-differences, given that dietary wariness is often thought to change with both age and accumulation of prior experience [9,50].

Almond trees are not native to Australia and are not commercially grown in the Sydney region. While local residents occasionally provision birds with almonds, these are likely always commercial bags of pre-processed nuts (*Penndorf, pers. obs.*). Almonds in the shell should therefore be rarely encountered by local SC-cockatoos, although likely not completely unknown. Taking advantage of this, we further investigated (i) whether processing techniques were consistent within individuals and (ii) whether these techniques were socially transmitted by comparing variation in opening techniques between individuals and roosting communities. If opening techniques were socially influenced, we predicted that the level of variation in opening technique would (i) be predicted by the social association network (collected through group scans), (ii) increase with increasing distance between roosting communities, and (iii) decrease with higher connectivity between roosts.

## 2 Results

### 2.1 Cultural diffusion experiment

#### 2.1.1 Spread of novel behaviour in three neighbouring roosts.

**Fig 1:**
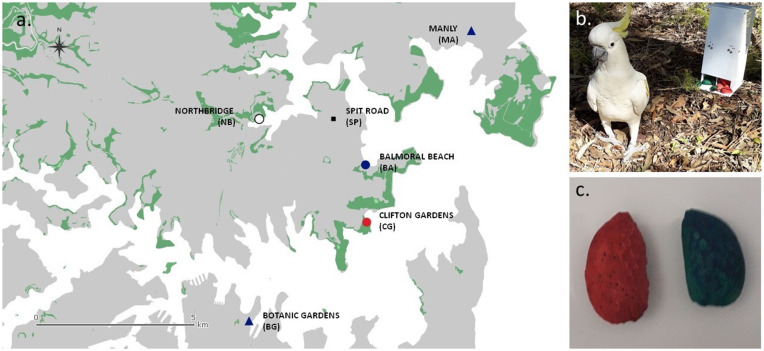
Study locations and set-up. **a)** Map of the roosting sites included in this study. The main roosts are represented with a circle. For these three roosts, social associations were recorded for the NBDA analysis. The secondary roosts are represented with a triangle. No social data was recorded at those sites, and the spread of the behaviour purely relied on individuals moving between the main and secondary roosts. The colours of each symbol represent the colour the two demonstrators were trained to use in each roost: red (Clifton Gardens—CG), blue (Balmoral Beach—BA, Manly—MA, and Botanic Gardens—BG), or white for the control group where no demonstrator was trained (Northbridge—NB). The black square represents an additional location where social data was collected (Spit Road, SP). The map was constructed in QGIS [51], using OpenStreetMap [52]. **b)** Male demonstrator of the CG roost during the preference test, shown in front of the experimental apparatus that dispensed blue and red coloured almonds. The dispensing side for each colour was alternated each day. **c)** Example of red and blue unshelled almonds. Almonds were safe for human consumption and were dyed using nontoxic food dye. Pictures by JP.

A resident pair (adult male and female) was trained in two roosting communities to overcome an initially strong averse reaction to accept either blue almonds (Balmoral Beach (BA, Fig 1A) or red almonds (Clifton Gardens CG, Fig 1A). No individual was trained in the third roosting community (Northbridge NB, Fig 1A), which served as a control. In one site (BA), the female rejected even unshelled almonds, and so one additional male was trained to match the number of demonstrators at each site. A dispenser containing both colours was then presented to all present individuals in daily experimental sessions at the same location nearby to the roost where social association data had previously been collected (Fig 1B). Sessions lasted until one option was depleted, or for a maximum of two hours. If one colour was depleted during the first hour, a second batch was presented at the beginning of the second hour of the experiment, in order to maximise the number of individuals that could participate in the experiment.

Demonstrators performed the trained behaviour shortly after the beginning of the first experimental session, and the behaviour was adopted by naive individuals within < 10 min (BA: 7 min, CG: < 1 min). The behaviour then spread quickly, with 19 individuals starting to consume almonds within the first session (BA: 5 individuals, CG: 14 individuals), following the demonstrators’ colour choice (blue for BA, red for CG). At both roosts, demonstrators also began to feed on the alternative colour (red for BA, blue for CG) on the first experimental day. In CG (trained on red), one demonstrator ate the alternative colour once (female_CG1_: 1 blue, 5 red; male_CG2_: 0 blue, 27 red). However, in BA (trained on blue), both demonstrators switched their colour preference on day one (male_BA1_: 2 blue, 14 red; male_BA2_: 4 blue, 5 red). Over the course of the experiment, all demonstrators then preferentially ate red almonds (male_BA1_: 0.76 red; male_BA2_: 0.68 red; female_CG1_: 0.77 red; male_CG2_: 0.84 red).

By contrast, in the control roost without trained demonstrators (Northbridge NB, Fig 1A), no individuals ate the novel food until day 4, despite it being available for 2 hours each day. The first individual to consume the novel food was a juvenile female, who had moved from the BA roost where she had previously observed conspecifics eating the novel food 130 times (18 blue, 112 red). She ate red almonds (0 blue, 8 red). Following this, the control roost started to feed on coloured almonds within 10 min, with 15 additional individuals adopting the behaviour before the session’s end. In total, over the 10 day experiment across the three roosts, 214 individuals fed at least once on the novel food (mean number of almonds eaten per individual: 28.9, range: 1–154; Fig 2), representing 62% of individuals. Within these individuals, a preference for red almonds was recorded at the group level for all three roosts (S1A, S1C, and S1E Figs).

**Fig 2:**
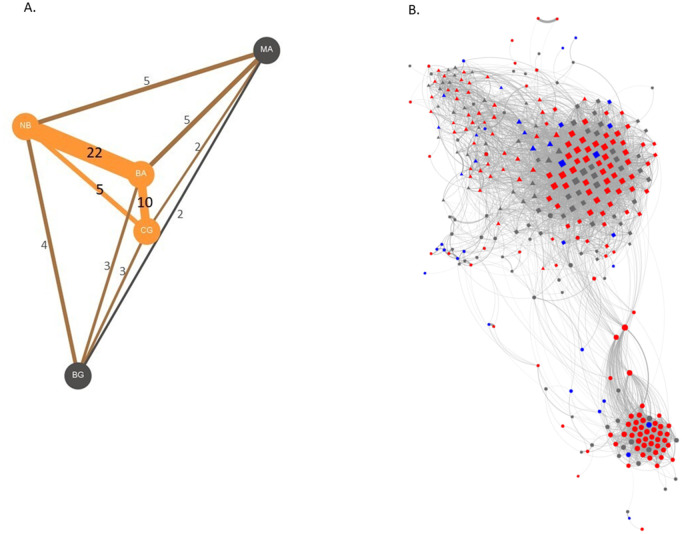
Social networks of the study population. **(A)** Network between neighbouring roosting communities, where edge labels represent the percentage of birds moving between sites. The network was constructed using GEPHI version 0.9.2 [53]. Orange circles represent the main roosts, grey circles the secondary roosts. **(B)** Social network recorded from the three neighbouring roost sites of BA (squares), CG (circles), and NB (triangles), including 322 individuals. Nodes represent individuals and the edges between nodes are scaled according to the proportion of all foraging observations where two individuals were observed foraging together during two 10 day social data collection periods. Node size indicates weighted degree, and nodes are coloured according to diet, i.e., red or blue if they ate the novel food (*n* = 197), and grey if they did not (*n* = 125). Node colour indicate individuals that displayed a preference for red (>50% solves on red) or blue (>50% solves on blue). The network was constructed in Gephi [53]. Edges below 0.10 are not shown for visual clarity. The data underlying this figure can be found in our data and code repository (https://doi.org/10.5281/zenodo.19052060).

#### 2.1.2 Spread of the novel behaviour beyond main roosts.

Given that the trained demonstrators at BA had lost their preference for blue on the first day of the experiment, we could not disentangle social learning of colour from an innate preference for red. We therefore expanded the experiment to two further roost-communities that neighboured either CG (BG) or BA (MA) (Fig 1A) over a second 10 day period. We did not train additional demonstrators at these sites, but relied on moving individuals, modifying the protocol to present blue almonds only on the first day a knowledgeable individual was present, and then switching to presenting both colour options on day 2.

In the two secondary roosts, the behaviour was introduced by moving individuals (MA: male of unknown age, 31 prior selections of red in BA; BG: juvenile of unknown sex, 56 prior selections of red and 49 of blue at BA and NB). After this, 39 naive individuals (BG: 17; MA: 22) started eating blue almonds prior to the introduction of the alternative colour. By the end of the 10 day experiment, 141 individuals (BG: *n* = 76, MA: *n* = 65) consumed at least one coloured almond (mean number of almonds eaten per individual: 32.4 [1–189]), representing 78% of individuals observed during the experimental period at BG and 70% observed at MA.

The alternative colour (red) was also consumed soon after it was offered, and both behavioural options were subsequently observed in the roosts. The roost level colour preference for blue was lost within 2 sessions (1 day) after the introduction of red almonds, reaching around chance level by the end of the experiment (S1B, S1D Fig). This supports the results from the main roosts, suggesting that there was a general underlying preference for red. However, a large variation was observed at the individual level: whilst in the main roosts, most individuals exhibited either a preference for red, or no preference at all (S1A, S1C, and S1E Fig), individuals in the secondary roosts exhibited strong preferences for either one of the colours or no preference at all (S1B, S1D Fig).

### 2.2 Network-based diffusion analysis

In the area of the three main roosts (Fig 1A), we visually conducted 864 group scans over two 12 day periods before and after the experimental period, during which we recorded the identity of all birds present. Using these, we constructed a social association network based on the co-occurrence of individuals in groups. Overall, 322 individuals were included in the network (Fig 2—including the three main roosts). This association network was then used in a *network based diffusion analysis* (NBDA), which assesses the relative importance of social transmission on the diffusion of knowledge about the novel food compared to an asocial model. This method infers that social learning is occurring if the spread of the behaviour follows the social network. We compared models both with and without social transmission and in all combinations of individual covariates (sex and age) (S1 Table), using Akaike Information Criterion corrected for sample size (AIC). We found strong evidence that the uptake of the novel food occurred through social transmission ($\Sigma AIC_{social}$ = 1, $\Sigma AIC_{asocial}$ = $\left[)3.2^{-56}\right)$). In the best performing model (ω = 0.28), 99.9% (95% CI: 96.5%–100%) of learning events were estimated to have occurred through social learning. Neither age nor sex affected an individual’s social or asocial learning rate (all Σω <0.28).

### 2.3 Analysis of learning biases

We then explored the potential social learning strategies that individuals might be influenced by when consuming coloured almonds. In particular, we were interested in assessing the contributions of individual and social information pertaining to four potential learning biases: 1) frequency-dependent social learning (conformity or anti-conformity), 2) male-bias, 3) same roost-bias, and 4) age-bias. To do so, we used a hierarchical dynamic learning model known as an *experience weighted attraction model*. This statistical approach estimates the joint contributions of individual learning (dynamic personal experience updated over time) and social learning (dynamic windows of social information corresponding to different social learning strategies) to predicting observed behaviour. More concretely, these models estimate the probability that an individual will produce a behaviour over time as a function of the weighted contributions of individual and social information.

Comparing widely applicable information criteria (WAIC) values and inspecting model predictions against raw data, we found that a positive frequency-dependent bias predicted our data better than any of the other considered models (Table 1). We then examined variations in performance in predictors and parameter estimates across all models across age and sex groups (S2 Table), as well as at the individual level (S2 Fig).

**Table 1:** Widely applicable information criteria (WAIC) estimates for all evaluated models with a 60 sec window of social information. fit_freq is a model with a positive frequent dependent bias; fit_male_lin is a model of bias towards copying the behaviour of males; fit_adult_lin is a model of bias towards copying the behaviour of adults; fit_roost is a model of bias towards copying individuals from the same roost; fit_i is the model for individual learning.

	WAIC	SE	dWAIC	dSE	pWAIC	weight
fit_freq	11,095.00	106.21	0.00		185.28	1.00
fit_male_lin	11,169.13	106.88	74.13	21.79	177.01	0.00
fit_adult_lin	11,187.93	106.82	92.94	21.51	170.02	0.00
fit_roost	11,196.83	106.59	101.83	28.57	228.87	0.00
fit_i	11,245.86	106.70	150.86	28.81	163.14	0.00

#### 2.3.1 Behavioural sensitivity (λ).

**Fig 3:**
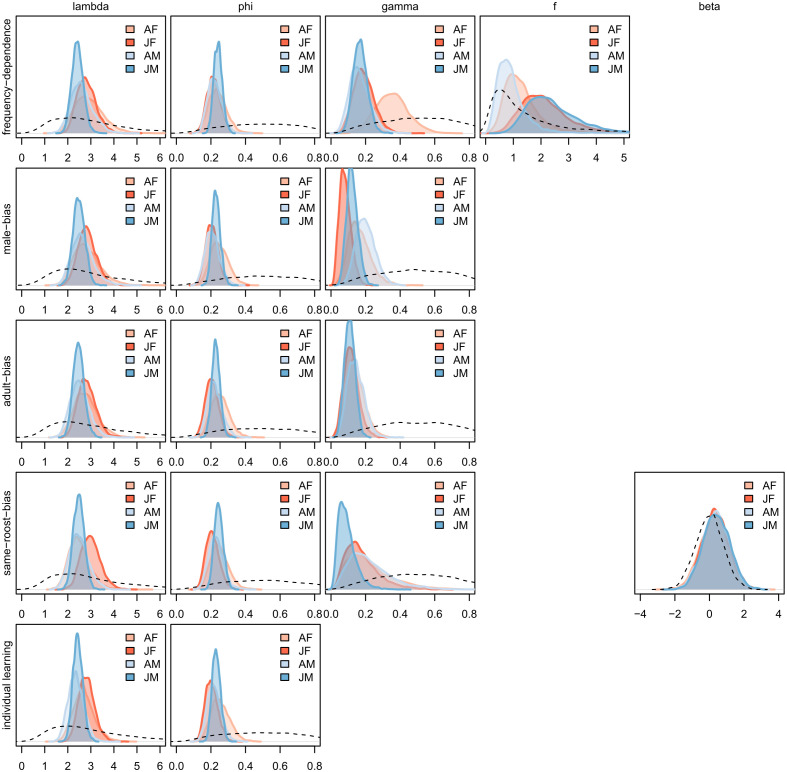
Posterior distributions of main learning parameters for each model. Only the posteriors for individuals with known age and sex classes are displayed. Each column is a parameter. Each row is a model. Dotted lines show the prior used in each model. The data underlying this figure can be found in our data and code repository (https://doi.org/10.5281/zenodo.19052060).

A higher sensitivity to differences in attraction scores indicates that individuals are more likely to switch their behaviour if information changes. Females ($\lambda_{jf}$= 2.83; $\lambda_{af}$= 2.90) tended to be more sensitive to differences in attraction scores than males ($\lambda_{jm}$= 2.41; $\lambda_{am}$= 2.53), while juveniles ($\lambda_{jf}$= 2.83; $\lambda_{jm}$= 2.41) tended to be more sensitive than adults of the same sex ($\lambda_{af}$= 2.90; $\lambda_{am}$= 2.53; S2 Table and Fig 3). These patterns were consistent across all models, suggesting they are robust.

#### 2.3.2 Weight given to personal experience (ϕ).

For all age and sex groups, ϕ was below 0.5, suggesting that individuals are more influenced by their memory of past personal experience than by new information (S2 Table and S1 Fig). There was little observed differences in this estimate across age and sex classes ($\phi_{am}$= 0.22; $\phi_{af}$= 0.23; $\phi_{jf}$= 0.21; $\phi_{jm}$= 0.24).

#### 2.3.3 Weight given to social information (γ).

Social cues varied in salience for each of the considered age and sex classes. For example, solves by males tended to influence the behaviour of other males ($\gamma_{am}$= 0.19; $\gamma_{jm}$= 0.12) more than they influenced the behaviour of females ($\gamma_{af}$= 0.15; $\gamma_{jf}$= 0.08). Adults ($\gamma_{af}$= 0.14; $\gamma_{am}$= 0.16) were slightly more influenced by the behaviour of members of the same roost than juveniles ($\gamma_{jf}$= 0.13; $\gamma_{jm}$= 0.09). Finally, there was no evidence for an age bias in copying (S2 Table and S1 Fig).

#### 2.3.4 Conformity (*f*).

**Fig 4:**
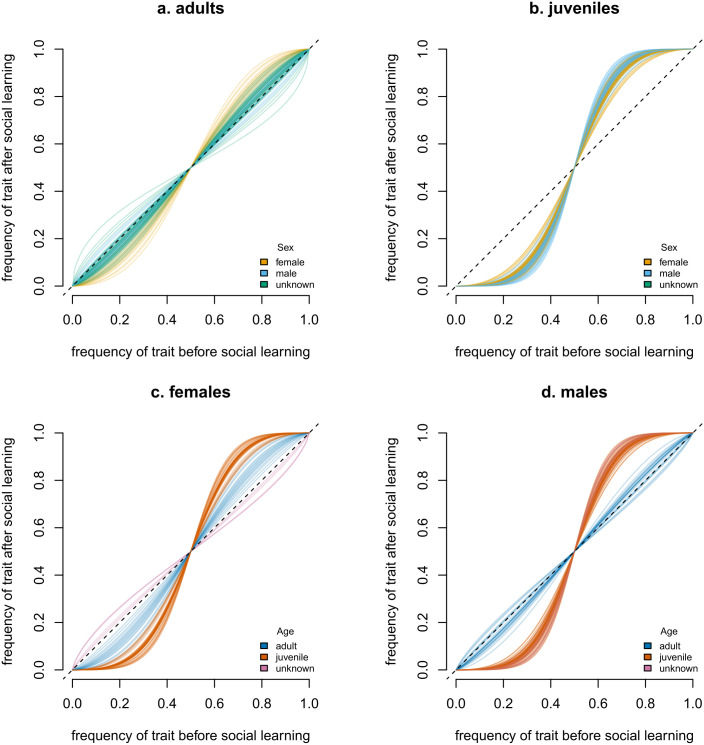
Individual-level acquisition curves comparing expressed choices against the frequency of behaviour produced in the previous 60 seconds, divided by age and sex class. The dotted line shows the expectation under unbiased copying, and the shape of the curve is the estimated posterior mean of *f* from the frequency-dependent learning EWA model. Results suggest that juveniles disproportionately choose the food colour that the majority chose in the 60s previous, while adults are not influenced by the frequency of other’s choices. **(a)** Adults (>7 years), with females in orange, males in blue, and birds of unknown sex in green. **(b)** Juveniles (1–7 years), with colours as per (a). **(c)** Females, with adults in blue, juveniles in orange, and birds of unknown age in pink. **(d)** Males, with age coloured as per **(c)**. The data underlying this figure can be found in our data and code repository (https://doi.org/10.5281/zenodo.19052060).

Juveniles of both sexes exhibited a positive frequency-dependent (conformist) bias (Fig 4—juvenile females: mean *f* = 1.67, posterior proportion > 1 = 0.86; juvenile males: mean *f* = 1.95, posterior proportion > 1 = 0.92). Adults by contrast, exhibit no, or a much weaker, conformist bias (Fig 4A). Adult females were weakly conformist or unbiased (mean *f* = 1.14, posterior proportion > 1 = 0.61), while adult males exhibited unbiased transmission or even weak anti-conformity (mean *f* = 0.89, posterior proportion > 1 = 0.43).

### 2.4 Opening techniques

**Fig 5:**
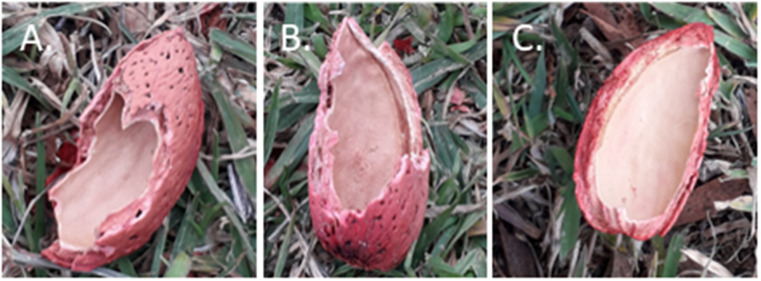
Foraging on shelled almonds by cockatoos. The pictures represent the remains of different opening techniques: two examples of “nibbling” starting **(A)** the hilum and **(B)** tip and an example of the **(C)** “splitting” technique. Pictures by JP.

**Fig 6:**
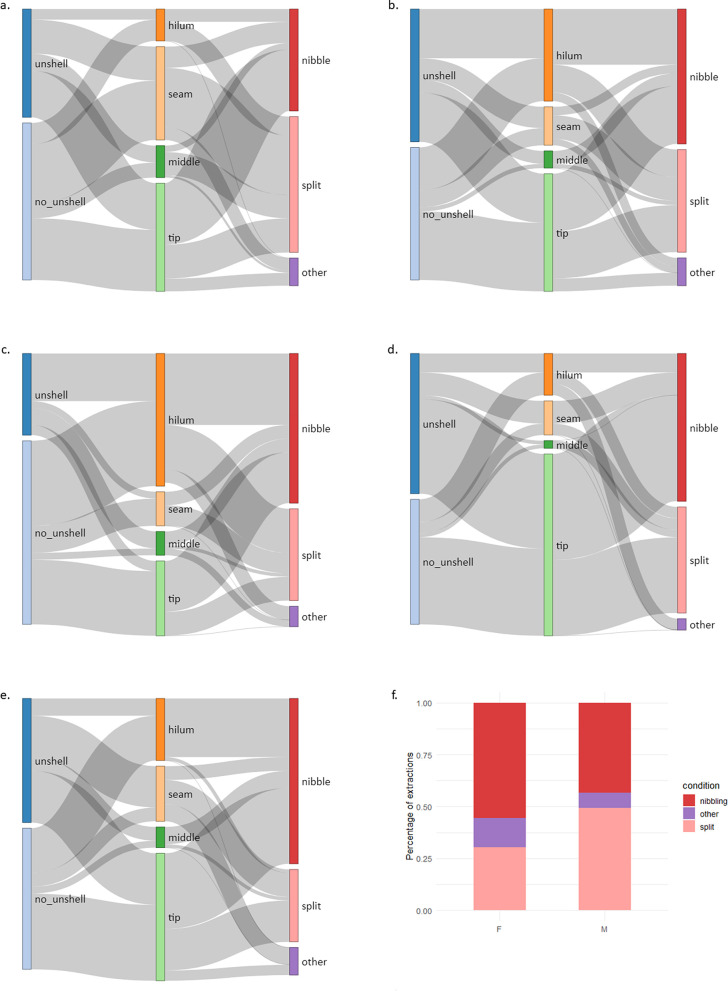
Behavioural sequences for successful almond openings across different roost sites (a: BA, b: BG, c: CG, d: MA, e: NB) and extraction techniques (f). Opening stages are depicted from left to right: (i) whether individuals remove the outer layers of the shell (optional step, binary), (ii) at which location the shell is initially cracked (4 options: tip, hillum, middle, seam), and (iii) how the almond is subsequentially extracted (3 options: nibble, split, other). Males (M) are more likely than females (F) to show the *’splitting’* technique (p = 0.01) at stage 3 (panel f), and sites show significant differences in extractive sequences (*p* = 0.001). The data underlying this figure can be found in our data and code repository (https://doi.org/10.5281/zenodo.19052060).

Almonds in the shell require opening before consumption—individuals often held the nut in their foot while stripping the shell with their bill (Fig 5). During the experiment, we recorded and coded 539 opening sequences from 147 individuals (median no. openings per individual = 3 [1–17]): Fig 6. We focused on three steps: (i) whether or not individuals exhibited the optional *’unshelling’* (removing the outer layers of the shell—binary variable), (ii) the location at which individuals first cracked the shell (4 options), and (iii) how the almond was extracted (3 options). We then created a dissimilarity matrix between each pair of sequences and conducted partial mantel tests to examine the effect of individual identity, social associations, and site. Our results revealed multiple sources of variation. First, opening sequences by the same individual were significantly more similar than sequences between different individuals (partial Mantel test, accounting for distances between roosting communities: *r* = 0.047, *p* = <0.001). Second, males extracted almonds using the “split” method (Fig 5C) significantly more often than females (*p* = 0.01; Chi-square post hoc test with Bonferri-correction *N*_*female*_=28, *N*_*male*_=52). Third, similarity between opening sequences decreased with increasing distance between roosting sites (partial Mantel test controlling for individual ID: *r* = 0.042, *p* < 0.001), and increased with increasing movement between sites (partial Mantel test controlling for individual ID: *r* = 0.022, *p* < 0.001; movement rates: 2% (MA—BG) to 22% (BA—NB), Fig 2A). Finally, opening sequences were more similar between close associates (partial Mantel Test controlling for individual ID: *r* = 0.011, *p* = 0.05—*N*_*ind*_=78, *N*_*openings*_=273), but not between genetically related individuals (partial Mantel test, accounting for individual ID: *r* = −0.0022, *p* = 0.62—*N*_*ind*_=79, *N*_*openings*_=327).

## 3 Discussion

Taken altogether, our results suggest four important findings. First, despite the generalist diet of SC-cockatoos and presumed prior familiarity with a similar food (naturally coloured almonds out of the shell) by many individuals, we found strong evidence that individuals acquired knowledge about novel food socially. Indeed, before observing another individual opening the coloured shells, individuals either ignored the food or exhibited active rejection (e.g., backing away). This suggests an important role for social learning in the formation of dietary repertoire of SC-cockatoos. Second, the rapid spread of knowledge about novel food suggests extensive potential in this species for adaptive dietary flexibility, with dietary knowledge spreading from 4 trained individuals in two roosting communities to 349 individuals in five roosts over the course of the experiment. Third, we found no evidence for age differences in learning, suggesting that this flexibility is not developmentally constrained or based on prior experience. However, clear age differences emerged when considering social learning strategies, with juveniles, but not adults exhibiting a striking conformity in their food choices. Finally, we find individuals used a variety of distinct opening sequences when opening almonds, and while all sequences were present at all sites, roosting communities showed differences related to the level of between-roost movement. This suggests a social influence on the acquisition of opening techniques, further suggesting that influence can lead to roost-level differences in foraging behaviour at relatively small spatial scales.

### 3.1 Behavioural flexibility

It has been suggested that one of the major adaptive behavioural responses exhibited by disturbance-tolerant species is the addition of introduced plants into their foraging repertoire [36,54]. This behavioural flexibility is likely to be especially important for long-lived and slow-reproducing species such as SC-cockatoos, where genetic evolution cannot keep pace with human-induced rapid environmental change [37]. Dietary flexibility has most often been considered an individual [55], or species-level trait [36], e.g., of innovativeness [56], and relatively fewer studies have considered a role for social learning [57]. However, our results suggest that the high dietary flexibility of SC-cockatoos [44,58] is also likely to have a social component, with the incorporation of novel foods facilitated by social learning. This could potentially help explain the success of this species across a broad range of environments. This aligns with previous studies in racoons, hypothesised at this species’ success in urban environments is based on their sociality (reviewed by [59]), but is at odds with other research in urban boat-tailed grackles [60].

In addition to social learning between individuals, our study documented a rapid geographic spread of knowledge, with information being carried by moving individuals over a matter of days between roosts of 1.5–3 km distance. This contrasts with another recent study on SC-cockatoos in the south of Sydney, where a novel foraging behaviour (bin-opening) took several years to spread 25 km from the presumed source [47]. The authors suggested that this could have been linked to one of two potential factors: (i) the cognitive difficulty of the task, with behaviour potentially taking a long time to socially learn and/or master, and (ii) the highly modular social network [47]. While our study was conducted at different locations, geographic distances between roosts (1.5–10 km), and the structure of within-roost social networks were comparable [43,47]. However, acquiring information about a novel food is cognitively less demanding, suggesting that the slow diffusion of the bin-opening behaviour is most likely explained by the difficulty of the task. This further illustrates the importance of considering the cognitive and social factors in tandem when predicting the diffusion of innovations [61,62].

### 3.2 Behavioural differences between neighbouring roosts

A further interesting comparison with [47] comes from our analysis of almond opening techniques. While we did not experimentally seed specific opening techniques, we observed that individuals were variable in how they opened almonds. A post hoc examination of this variation revealed a weak social and spatial correlation; within roosts, individuals were more similar to close social associates, while between roosts, similarity decreased with greater distance and lower movement rates. Given the scale of the study, these observed differences are unlikely to be driven by phenotypic, ecological, or genetic differences [43]. We would further argue that differences are unlikely to be driven by variation in the size or the structural properties of the almonds, as all almonds originated from the same batch. Therefore, it seems more parsimonious that these differences represent emergent cultural variation.

Cultural differences in foraging techniques are now well described in groups like primates and cetaceans [63–67]. However, these differences have largely been described at relatively large geographic scales, and the subtle behavioural differences that may occur between neighbouring groups of the same population have received comparatively little attention ([68]—though see [47,69–73]). Our work adds to the recent emerging evidence that, when behavioural sequences are examined at fine scales, then cultural differences can also be observed at surprisingly local scales. Our results reflect the sub-cultural variation in bin-opening techniques found in [47], supporting the idea that modular, partially-connected social networks such as that observed in SC-cockatoos could support both the rapid spread of information and the emergence of local cultures [74,75].

### 3.3 Colour-preferences

As part of our experiment, we seeded different roosts with demonstrators trained to prefer either red or blue coloured almonds. However, despite evidence for social learning of the novel food, and evidence for emergent roost-level differences in opening techniques in our experiment and previous work [47], roosts did not maintain a local tradition for food colour, with roosts seeded on blue—but not red—losing their initial colour preference over the course of the experiment. It seems likely that this was due to a general underlying preference for red; this would also explain why the demonstrators in one group (BA) quickly changed to opening red almonds.

Such preferences for red foods have been described in other bird species (Crimson rosella, *Platycercus elegans*: [76]; blue tits, *Cyanistes caeruleus*, and great tits, *Parus major*: [77]). Yet exposure to coloured food has also been shown to overcome innate preferences, for example, crimson rosellas exposed to blue grains for two weeks maintained a preference for blue grains after the preferred colour (red) was introduced [76]. Perhaps in the case of our study, the previous exposure to blue almonds was too short to overcome prior preferences.

Previous studies have also suggested that colour-biases can depend on age and previous experience. For example, juvenile tits (*Cyanistes caeruleus* and *Parus major*) exhibit a preference for red, while adults exhibit a preference for red only after a positive experience with this colour [77]. Similarly, naive hand-raised blackcaps (*Sylvia atricapilla*), but not wild caught adults, have a preference for red food [78]. Individually-varying preferences can not be implemented in the current version of the EWA model and so we were unable to explore this further. However, if so, it would most likely influence the weight given to social information by different classes γ, rather than the estimates of social learning strategies. As such, underlying preferences are likely common; a valuable future direction would be to incorporate innate preferences alongside reinforcement and belief learning into the EWA modelling framework [79] with a >2 option task problem to increase analytical leverage [80].

### 3.4 Age-differences in social learning

Our NBDA gave no evidence that age influenced an individual’s social learning rate about novel food. By contrast, our EWA model suggested that for colour choice, juvenile SC-cockatoos may rely on social information less than adults. First, while the EWA model identified age differences in the weight given to social information γ, the posterior distributions of both parameters overlapped; results should be interpreted with caution. Second, the models measure different social learning processes: NBDA estimates social influence on *acquisition* of behaviour, while EWA-models estimates social influences on its *production*, based on the assumption that individuals have existing knowledgeable of all behavioural variants [81]. This suggests that there is no strong effect of age on social learning, in line with a recent meta-analysis [82]. However, our experiment did reveal striking age differences in social learning strategies, with juveniles, but not adults, exhibiting conformity.

Conformity (here defined as a positive frequency-dependent copying bias: [83,84]) may be particularly advantageous when encountering new food sources and adaptive behaviour is common, as it allows individuals to avoid potentially toxic foods even without any prior knowledge about the local environment. For example, migrant male vervet monkeys adopted the artificial preference of their new group for a specific colour of corn, despite having fed on the alternative colour in their previous group [33].

In SC-cockatoos, movement events are relatively frequent; individuals of both sexes switch roosting communities on average every 2.5 years [45] and regularly visit neighbouring roosts [43]. In Sydney, the environment varies over small geographic scales; for example, with different (often exotic) street trees planted across local council areas. Given this, we hypothesise that selection should favour a general reliance on conformity [85], especially since costs associated with individual learning (here, potentially toxic food) are high [85]. Our finding that juveniles, but not adults, are conformist is therefore surprising, and contrasts with other studies showing an increase in conformity with age [86]. Yet, juvenile SC-cockatoos move more frequently than adults [43], and in adults, movements more often appear to be triggered by particular events (e.g., losing a nest hollow; *personal observation*). There are, therefore, two nonmutually exclusive possibilities. First, social learning strategies may change across development. In this system, juveniles are likely to have less local information than adults, and so may use conformist learning as a short-cut to gain the best local knowledge. This explanation is supported by our finding that only adults preferentially weigh social information provided by members of the same roost, a strategy which implies individual recognition and knowledge of the residence status of other individuals [87,88]. Second, shifts in learning strategies may be triggered during periods of dispersal or transience. This was recently shown in a captive experiment in tits, where individuals shifted learning biases when moved into new groups in different environments [89].

In humans, the strength of conformity has been suggested to be age dependent [90,91]. For example, when given the opportunity to update their prior beliefs using social information, young children (<6 years) only updated their behaviour when all social demonstrators were in agreement, while older children (>7 years) conformed to the majority [92]. The authors suggested that with age, children increasingly exhibit learning styles of adults ([92]—but see [90]). Yet, developmental differences are difficult to disentangle from enculturation in humans (i.e., learning to learn), and levels of adult conformity show large differences across cultural groups [90]. How learning biases may change over an animals life-time has received little theoretical [93–95] and empirical attention, outside of whom to copy (reviewed by [96], and see [86]). How the level of conformity may change across development in animals has, to our knowledge, not been explored. Further research is therefore needed to investigate age-dependent variation in conformity in nonhuman animals, as well as to investigate whether these patterns extend to other contexts in SC-cockatoos (e.g., to puzzle-box tasks).

### 3.5 Conclusion

In conclusion, we provide experimental evidence that dietary knowledge about new foods is socially transmitted in SC-cockatoos, the first such study to our knowledge in a wild parrot. This social learning appears to include both food type and processing techniques, with the partially-connected, modular networks of SC-cockatoos leading both to rapid spread and potentially the emergence of cultural variants. We further find that, even after the initial acquisition of the behaviour, individuals continue to update their behaviour based on recent social information. Yet, how individuals update their behaviour varies with age, with juveniles, but not adults, preferentially selecting the food choice of the majority. The utilisation of new food resources is a fundamental component of adaptive behavioural responses to novel environments. Understanding how animals adopt new food items is therefore of increasing importance in a world undergoing rapid and widespread anthropogenic change, to predict—and potentially improve—human wildlife coexistence. Our study demonstrates how cognitive and social influences can be vital determinants of this adaptive flexibility.

## 4 Methods and materials

### 4.1 Study population

We conducted the study on an urban-living population of SC-cockatoos in central Sydney (Australia—Fig 1A). In this area, SC-cockatoos form year-round communal sleeping roosts of 50–300 individuals. Such communal roosts consist of both nonbreeding and breeding individuals, with breeding individuals defending tree-hollows in close proximity to the main roost [45]. When foraging, roosting communities break into smaller subgroups [58,97] of fluid group membership that potentially include individuals of other roosts. Birds return to the roost site at regular intervals throughout the day, and individuals of different roosting communities also often visit neighbouring roosts [43,45].

The study encompassed five neighbouring roost-sites in northern Sydney (Fig 1A), located at Balmoral Beach (BA; −33.828494, 151.253983), Clifton Gardens (CG; −33.841444, 151.252889), Manly (MA; −33.796200,151.282786), Northbridge/Cammeray (NB; −33.817167, 151.221694) and the Royal Botanic Gardens (BG; −33.864776, 151.220025). At each roost site, the birds were attracted to the ground using small amounts of sunflower seeds at the same time each day, and over several weeks. Once the individuals were habituated to the observer, they were temporarily and noninvasively marked using nontoxic dye (Marabu Fashion Spray, MARABU GMBH), applied with sponges on the middle of the back. Each individual was marked with a unique combination of one to three coloured dots (methods detailed in [43]). In total, 550 birds were marked with nontoxic dye, and 11 birds were recognisable through natural variations in morphology. In addition, 144 birds were equipped in 2010–2016 with yellow cattle marks as part of the ongoing citizen science project *Big City Birds* [42,45], and some of these birds were present in the study area. Overall, 349 marked or recognisable individuals were observed regularly, and included in the study.

Adults (>7 years) were sexed based on eye-colour (black: male, red: female). Juveniles (<7, brown eyes) are fed by parents for approximately 9–12 months after leaving the nest, but remain sexually immature until 7 years of age. Given the time of year the study was conducted, all juveniles were assumed to be independent of parents, and none were observing begging. Juveniles were therefore aged by eye colour as 1–6 years, but could not be morphologically sexed. However, feather samples collected for a parallel study [43] were used to verify assigned sexes of adults, and to sex juveniles when possible (205 samples across all five the roosting locations: N_BA_ = 68, N_CG_ = 55, N_NB_ = 41, N_BG_ = 17, N_MA_ = 24). Altogether, this resulted in 106 males (78 adults, 21 juveniles, 17 of unknown age), 76 females (55 adults, 16 juveniles, 5 of unknown age), 44 juveniles of unknown sex, and 102 individuals of unknown age and sex being included in the study.

All procedures were approved by the ACEC (ACEC Project No. 19/2107), and were conducted under a NSW Scientific License (SL100107).

### 4.2 Social and relatedness data

#### Social data collection.

Before and after the cultural diffusion experiment, we recorded social association data at three main sites (BA, CG, NB, Fig 1A), for 3 hours per day in July (July 8—July 20) and 2.5 hours per day in September (September 19—October 2) 2019. Prior to each session, birds were encouraged to start ground foraging by scattering sunflower seeds over a 385–500 *m*^2^ area at <700 m distance to the roost. Once the birds started foraging on natural resources (i.e., digging for roosts or feeding on seeding grasses), we started monitoring their social behaviour. To do so, group scans [48] were conducted every 10 min, recording the identities of all individuals present (defined as present in the study area, either on the grass or in trees). In order to capture between-roost social associations, group scans were also conducted at a fourth location where individuals from all three roost sites mixed when being fed by local residents (Fig 1A).

To assess roost membership, we conducted three roost-counts at each of the main sites (July 17th–21st, September 15th–20th, and September 30th–October 3rd, 2019). At each count, the experimenter arrived at the roosting site pre-dawn and attracted the birds to the ground as soon as the roost started to be active, then recorded the identities of all individuals present. Roost-membership was determined if a bird was recorded at least two out of three counts at the same location. As roost-membership was highly correlated with marking site (Pearson’s Chi-squared test, *X*^2^ = 178.67, df = 4, *p* < 0.001, *n* = 153), we used the latter as proxy for roost-membership for those individuals that were not observed during roost-counts.

#### Social association networks.

We pooled social data from both observation periods, giving 864 scans across the three main sites. Using a gambit of the group approach [98], we constructed a social association network for the entire population using the simple ratio index (SRI), where dyads were assigned an edge strength that ranged between 0 (never observed in the same scan) to 1 (always observed together). All network analyses were conducted using the R-package *asnipe* [99]; for more detail see [43].

#### Movement between roosting communities.

To estimate the degree of movement between the 5 roosting sites in our study, we calculated the degree of overlap between roosting communities was calculated as follows:


$$m_{AB}=\frac{n_{ov}}{n_{A}+n_{tB}-n_{ov}}$$

with *m*_*AB*_ being the movement rate between roosts A and B, *n*_*ov*_ being the number of individuals present for the experiment at both roosts A and B, *n*_*A*_ the number of birds present at roost A, and *n*_*B*_ the number of birds present at roost B. Given that birds regularly move between roosting-communities, birds can appear twice in the data set. For example, the individual “teal-orange-pink vertical” participated in the experiment at three sites (BA, NB, MA), and therefore contributed to the overlap between BA and NB, MA and NB, as well as MA and BA.

#### Relatedness.

As part of a different project, feather samples were opportunistically collected across all three roosting locations by plucking feathers (1–5) from the back of individuals. These samples were used to assess relatedness between individuals (for details, see [43]).

### 4.3 Diffusion experiment

#### Training.

At two roosting sites (BA, CG), we trained one breeding pair underneath their nest hollow to act as the initial demonstrators. The dyad at BA were trained to eat unshelled almonds dyed blue (Queen Blue, Fig 1C), and the pair at CG were trained to eat unshelled almonds dyed red (Queen Pillar Red, Fig 1C). At the control roost location (NB) no training was given (Fig 1A).

The demonstrators were attracted down under their nest hollow with sunflower seeds, and then first presented with natural unshelled almonds. Three of four birds accepted the food item, suggesting they had prior knowledge of unshelled almonds, or were generalising from similar, locally available nuts (male and female at CG, male at BA). At BA, a second adult male who was frequently in vicinity of the tree hollow was therefore trained as the second demonstrator. Second, birds were presented with coloured almonds. This induced strong negative reactions from all individuals (e.g., backing away, or taking and throwing the item). We therefore first trained demonstrators to accept almonds where half of the coloured shell had been sawn off. Once individuals accepted coloured, half-opened almonds, we moved to presenting them with unshelled, coloured, almonds.

Once all demonstrators took coloured almonds without hesitation, we conducted a preference test where we presented demonstrators with both options (Fig 1B). All male demonstrators (2 in BA, 1 in CG) chose their trained colour for 7/7 trials, and the female demonstrator in CG chose the trained colour in 6/7 trials.

#### Diffusion experiment: main roosts.

Once demonstrators were trained, we conducted a two-option and control cultural diffusion experiment at each of the three main roosts (NB = control, CG = trained on red and BA = trained on blue). Over 10 days between August 14th and August 25th 2019, all three roosts were presented with a dispenser in which both options (red or blue unshelled almonds) were freely available (Fig 1B). Daily sessions started at 7h00 for NB, 10h30 for CG and 13h30 for BA, and lasted until one option was depleted, for a maximum of two hours. If one colour was depleted during the first hour, a second batch was presented to the roost at the beginning of the second hour of the experiment (8h00 for NB, 11h30 for CG, 14h30 for BA), in order to give access to as many individuals as possible.

The dispenser was installed daily on the ground at the same location at which social data was collected (except the spit road site), at <700m distance to the respective roost. A Panasonic HD camcorder was installed on a tripod at approximate 1.7m distance from the dispenser, so that it could view both the identity of the individual taking the almond as its choice, and the immediate surrounding area. Additionally, we conducted presence scans [48] every 10 minutes throughout the experiment.

#### Diffusion experiment: secondary roosts.

The demonstrators at BA lost their trained preference for blue on the first day of the experiment, instead exhibiting a preference for red almonds. This effectively meant initially, most demonstrations witnessed by naive individuals were on red, and we could not disentangle social learning of colour from a general preference for red. We therefore expanded the experiment to two additional neighbouring roosts (BG and MA, Figure 1a) over a 10 day period between August 30th and September 14th, 2019.

Given that movements between neighbouring communities are frequent [43], we did not train additional demonstrators. Instead, we relied on moving individuals that had previously learned to forage on coloured almonds to introduce the behaviour. We modified the protocol such that both roosts were initially only presented with blue almonds, in order to seed the knowledge of blue almonds. The first session only started when least one previously knowledgeable individual was present. Once one kilogram of blue almonds had been eaten (2nd day at both sites), the alternative option (red) was introduced alongside the blue.

#### 4.3.1 Video coding of the experiment.

All videos were analysed in BORIS (v. 7.9.5,2019-11-27, [100]). For each solve (defined as one individual picking up an almond), we recorded (i) the time of the selection, (ii) the ID of the solver, (iii) the colour of the chosen almond, (iv) whether the individual dropped the almond while on screen. Unmarked individuals were rare, and not included in the final EWA-model. However, solves of unmarked individuals were recorded, and contributed to the social information witnessed by other individuals. All videos were coded by JP and one assistant. Inter-reliability scores were high (>0.9, 5% of videos re-scored).

#### 4.3.2 Analyses.

**Network based diffusion analysis (NBDA).** To investigate (i) whether individuals socially learned to feed on coloured almonds (regardless of colour), and to (ii) assess the relative strength of a potential social learning effect compared to asocial learning, we performed a *network-based diffusion analysis* (NBDA, [101–103]) where we compared the social network data with diffusion data from the three main experimental sites (BA, CG, NB) the R-package *NBDA* version 0.9.4 [104] in R-4.0.4 [105].

We applied the “*order of acquisition diffusion analysis*” (OADA) variant of NBDA with multiple diffusions (one at each of the main three sites—BA, CG, NB) through the association network at population level. We considered the order with which individuals first consumed coloured almonds (regardless of colour) as diffusion data. Additionally, we controlled for two individual-level variables (ILVs) that could potentially influence an individual’s rate of acquisition: sex (coded as −0.5 for females, 0.5 for males, and 0 for individuals of unknown sex), and age (coded as −0.5 for juveniles, 0.5 for adults, and 0 for individuals of unknown age). We fit unconstrained models that allow ILVs to influence social and asocial learning rates independently [106]. We fit models in all possible combinations of ILVs, with and without the social network (without social network to test for a model of asocial learning only). We calculated variable support using the Akaike Information Criterion, corrected for sample size [107].

**Experienced weighted attraction models (EWA).** In a second analysis, we explored the potential social learning strategies that individuals might be using when deciding on colour choice. In particular, we assessed four potential learning biases: frequency-dependent social learning (conformity, unbiased learning, or anti-conformity), male-bias, same roost-bias, and age-bias. To do so, we used a hierarchical dynamic learning model known as an *experience weighted attraction model* [34,35,79,86,108–110]. These models estimate the probability that an individual will produce a behaviour over time as a function of the weighted contributions of individual and social information. They can be modified to for heterogeneity amongst individuals or by any state variable (i.e., age or sex).

EWA models have an individual and social information component. The probability of individual *j* choosing behaviour *i* at time *t* is a convex-combination of their individual (*I*_*ij*,*t*_) and social (*S*_*ij*,*t*_) information, weighted by $\gamma_{j}$, the weight of social information which is between 0 and 1.


$$\mathrm{Pr}(i|A_{ij,t},\Theta_{j})=(1-\gamma_{j})I_{ij,t}+\gamma_{j}S_{ij,t}$$ (1)

**Personal Information:** The individual information contains an *attraction score*, which is a scalar value that represents accumulated personal experience with behavioural choice that is updated over time—a commonly used mathematical formulation of reinforcement learning in the cognitive sciences [111]. Attraction scares are represented as:


$$A_{ki,t+1}=(1-\phi_{ij})A_{ik,t}+\phi_{ij}\pi_{ik,t}$$ (2)

where *A*_*ki*,*t*_, unique to each behaviour *i*, individual *j*, and time *t*. At each time step *t* + 1, an attraction score for behaviour *k*, *A*_*ij*,*t*+1_, is upda*t*ed as a function of the attraction score at the previous timestep, *A*_*ik*,*t*_, and an observed payoff. $\pi_{ki,t}$. If an almond is not opened, animals experience a payoff of $\pi_{ki,t}=0$, and they experience a payoff $\pi_{ki,t}=1$ if an almond is successfully opened. The parameter $\phi_{i}j$ controls the importance of recent experienced payoffs relative to previous experience in influencing attraction scores. When $\phi_{i}j$ is high, more weight is given to recent experience over past experiences. When $\phi_{i}j$ is low, individuals are greater influenced by their memory of past experience. If $\phi_{i}j$=1, individuals have no memory and if $\phi_{i}j$= 0 they never update their beliefs from their initial state.

Attraction scores were converted into probabilities of behavioural choice using a softmax function:


$$\mathrm{Pr}(k|A_{ki,t},\lambda_{ij})=\frac{\exp(\lambda_{ij}A_{ki,t})}{\sum_{K}\exp(\lambda_{ij}A_{ki,t})}=I_{ij}$$ (3)

$\lambda_{ij}$ is lower bound by zero and controls sensitivity to differences in attraction scores. It is unique to an individual *j*. Choice is random with respect to attraction score when $\lambda_{ij}=0$, proportional when $\lambda_{ij}=1$, and grows until becoming deterministic.

**Social Information:** Social cues were incorporated into *S*_*ki*,*t*_ depending on the learning strategy. For frequency-dependent social learning, the probability of displaying a behaviour *k*, solely as a function of social cues, is:


$$S_{ki,t}=\frac{N_{ki,t}^{f_{ij}}}{\sum_{m}N_{Ki,t}^{f_{ij}}}$$ (4)

Here, *N*_*ki*,*t*_ are the observed frequencies of each technique *k* at time *t* by individual *i*. The parameter *f*_*ij*_ controls the amoun*t* and type of frequency dependence, and is calculated on the log scale. When *log*(*f*)=0, social learning is unbiased by frequency. When *log*(*f*)>0, social learning is positive frequency-dependent or conformist. Finally, when *log*(*f*)<0, social learning is negative frequency-dependent and a bias is shown towards less common behaviours.

Cues associated with demonstrators, namely membership in the same roost were incorporated via:


$$S_{ki,t}=\frac{\exp(\beta_{i,t}\kappa_{ki,t})}{\exp(\beta_{i,t}\kappa_{Ki,t})}$$ (5)

Here, $\beta_{i}$ is the strength of cue bias, namely roost-bias, and the cue values $\kappa_{ki,t}$ are 1 or 0, associated with whether an individual is a member of the same roost. For computational ease, we used the alias method [112]; rendering $\exp(\beta_{i,t}\kappa_{ki,t})=1$ for *k* = 1.

For male and age bias, we used the number of behaviors observed in that social category (male or adult) at time *t*. This model is identical to frequency-dependent learning where *f* is fixed to 1 to one and count of behaviours are tallied in the demonstrator group of interest. We estimated varying effects of individual, as well as a fixed-effect interaction between index variables of age and sex class, for the primary EWA parameters:


$$log(\lambda_{ij})={\bar{\alpha }}_{\lambda }[age_{j},sex_{j}]+\alpha_{\lambda ,i}$$ (6)

$$logit(\phi_{ij})={\bar{\alpha }}_{\phi }[age_{j},sex_{j}]+\alpha_{\phi ,i}$$ (7)

$$logit(\gamma_{ij})={\bar{\alpha }}_{\gamma }[age_{j},sex_{j}]+\alpha_{\gamma ,i}$$ (8)

$$log(f_{ij})={\bar{\alpha }}_{f}[age_{j},sex_{j}]+\alpha_{f,i}$$ (9)

$$\beta_{ij}={\bar{\alpha }}_{\beta }[age_{j},sex_{j}]+\alpha_{\beta ,i}$$ (10)

Age categories included adult, juvenile, and unknown and sex categories included male, female, and unknown. All social variables were estimated based on a 60 s window prior to an individual’s solve, with this window derived from a visual assessment of the average period which birds were likely to direct attention towards the dispenser before making a choice. In total, we fit five models. One model evaluated pure individual reinforcement learning. Four others combined individual learning alongside social information from the following learning biases: frequency-dependent social learning, male-bias, same roost-bias, and age-bias. We initialised attraction scores for the 4 human-tutored individuals to be 1 for the tutored option, and 0 for the non-tutored option. All nontutored individual had their initial attraction score set to 0, indicating no starting preference. All models were fit using the *cmdstanr* interface [113] to *R-Stan* [114] in *R* [105]. Models were fit using 4 chains, with 1,000 sampling iterations, and a warm-up of 1,000 iterations, while multi-threading 8 cores per chain.

### 4.4 Opening techniques

During the social learning experiment, we opportunistically filmed all observable almond openings, using a handheld camera (Nikon D3000). As multiple individuals were often processing almonds at the same time, we prioritised capturing opening behaviour for the largest possible sample and capturing observations with a full sequence from the unopened almond to full consumption.

#### 4.4.1 Video coding (opening techniques).

All videos were coded using BORIS (version 7.9.5, [100]) by JP and a research assistant. For each opening, we recorded 3 variables (Fig 5A–5C) describing the opening procedure in which individuals engaged:

1. **unshell**: an optional step, where individuals removed the outer layers of the shell, before moving on to the subsequent steps. This was encoded as a binary variable (*TRUE/FALSE*).
2. **crack**: the location at which individuals first cracked the shell (options: *tip/ seam/ hilum/ middle*).
3. **extract**: how the almond was extracted. This could be done either by *nibbling* (breaking small pieces of the shell, to access small parts of the almond—and repeating the process until the almond is entirely eaten), *splitting* (breaking the shell open to extract the entire almond), and *other* (extending the initial hole made during the *crack* stage, before extracting the entire almond).

For a subset of openings (*n* = 52), we additionally coded the duration of the opening process, starting from the moment the individual picked up the almond, and ending when the last piece of almond disappeared in the beak.

Inter-reliability was high (Cohen’s Kappa >0.70, 39% of videos scored by both observers, [115]) for all variables, R-package *irr* [116]. While this study aimed to analyse individual- and site-specific differences, neither JP nor the research assistant had knowledge as to which of the variables scored could be affected.

#### 4.4.2 Analysis.

We normalised each of the four behavioural variables before calculating a dissimilarity matrix between each pair of sequences using the Euclidian distance using the R-package [117]. We then compared this to four putative drivers of between-site differences:

- distance between roosting locations, where we expect differences in opening sequences to increase with increasing distances between roost;
- movement, with highly connected sites likely to exhibit more similar opening sequences;
- social network, where we expect a greater similarity between opening sequences of close associates;
- relatedness matrix: where, if differences in opening sequences are due to genetic predisposition, we expect related individuals to exhibit similar sequences.

To test these predictions, we used four partial Mantel tests (R package *vegan*, version 2.5–7, [118]), each assessing the effect of one of the above cited predictors on differences in opening sequences while controlling for individual ID. In a second step, we tested the influence of individual ID on opening sequences in four additional partial Mantel tests, while controlling for above cited predictors.

## Supporting information

S1 Fig Probability of choosing a red almond during each session at each site. Each panel represents one roost. The main groups (BA, CG, NB) are represented in the left column, the secondary roosts (BG, MA) in the right column. In the secondary roosts, choice was constrained to blue for the first two sessions. Dark red lines represent choices made by single individuals, and the bright red line represents the roost mean. Trained demonstrators have been removed. The data underlying this figure can be found in our data and code repository (https://doi.org/10.5281/zenodo.19052060).
(PDF)

S2 Fig Model predictions of the probability of choosing red across foraging bouts. Each panel represents one individual. The ID, sex, and age of the individual are indicated at the top of each panel. The top row of each graph represents the colour choice of the individual (red or blue) at each foraging bouts. Filled dots represent success, while empty circles represent failures (i.e., individuals dropping the almond with 3 s after picking it up from the dispenser). Coloured lines represent the prediction by each of the considered models. The roost(s) recorded at the top of each graph show at which site(s) an individual solved over the course of the experiment. The data underlying this figure can be found in our data and code repository (https://doi.org/10.5281/zenodo.19052060).
(PDF)

S1 Table Description of the five best NBDA-models, and their respective Akaike weights. (PDF)

S2 Table Parameter estimates for each age (a = adult, j: juvenile, u: unknown) and sex (f: female, m: male, u: unknown) group and each model. (PDF)

S1 Video Video 1: Video of opening techniques. The individual cracks at the tip of the almond, before extracting the nut by splitting.
(MP4)

S2 Video Video 2: Video of opening techniques. The individual cracks at the tip of the almond, before extracting the nut by nibbling.
(MP4)

S3 Video Video 3: Video of opening techniques. The individual extracts the nut by nibbling.
(MP4)

S4 Video Video 4: Video of opening techniques. The individual extracts the nut by splitting.
(MP4)

## Abbreviations

AIC: Akaike Information Criterion
ILVs: individual-level variables
NBDA: network based diffusion analysis
OADA: order of acquisition diffusion analysis
SRI: simple ratio index
WAIC: widely applicable information criteria
