# Supplementary material for: Wild parrots exhibit age-dependent conformity when learning about novel food
Source: PLoS Biol. 2026 Apr 30;24(4):e3003741. doi: 10.1371/journal.pbio.3003741 (PMC13132227; doi:10.1371/journal.pbio.3003741)
Supplement: S1 Fig — Each panel represents one roost. The main groups (BA, CG, NB) are represented in the left column, the secondary roosts (BG, MA) in the right column. In the secondary roosts, choice was constrained to blue for the first two sessions. Dark red lines represent choices made by single individuals, and the bright red line represents the roost mean. Trained demonstrators have been removed. The data underlying this figure can be found in our data and code repository (https://doi.org/10.5281/zenodo.19052060). (PDF) [file pbio.3003741.s001.pdf]

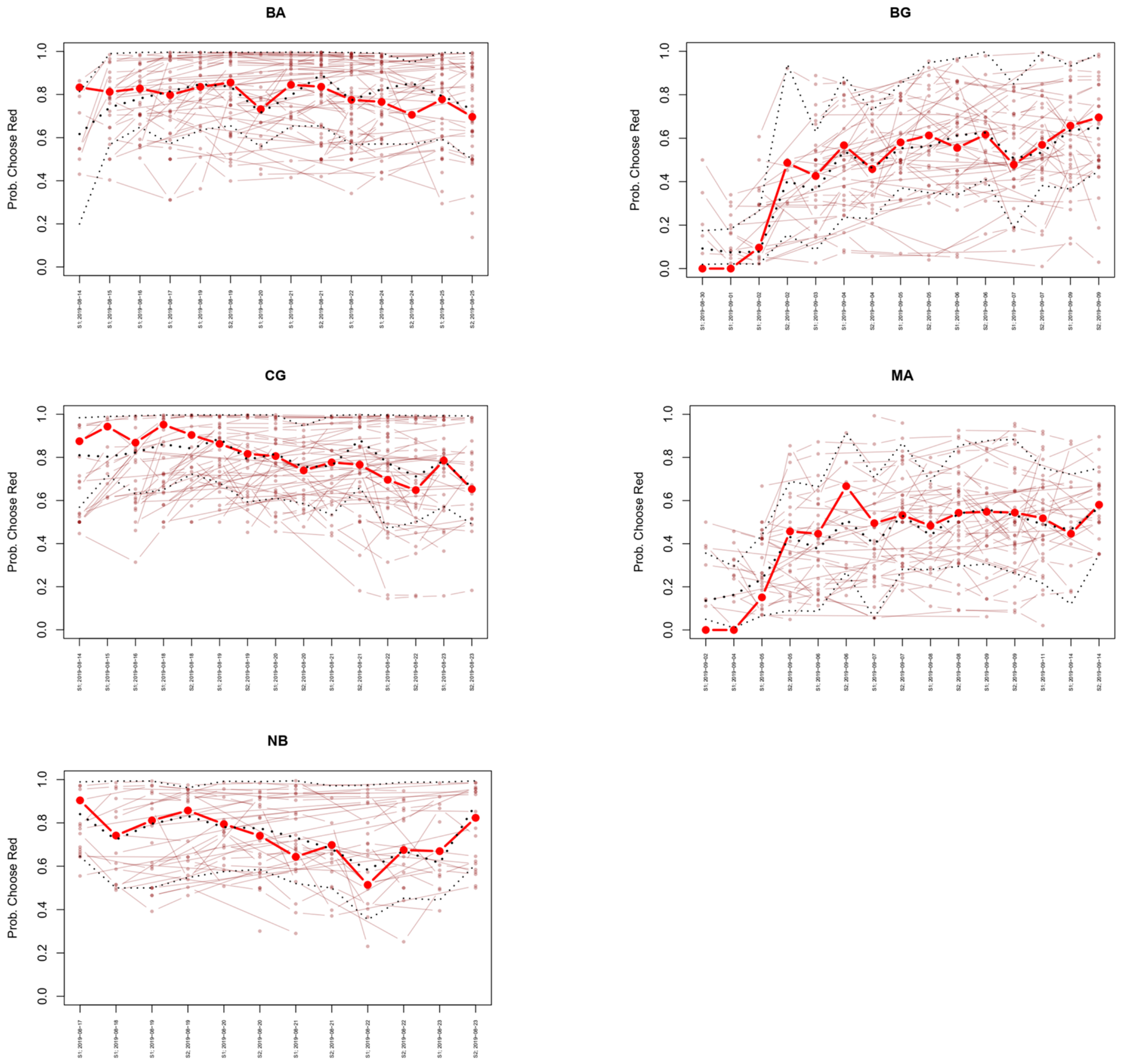


Probability of choosing a red almond during each session at each site. Each panel represents one roost. The main groups (BA, CG, NB) are represented in the left column, the secondary roosts (BG, MA) in the right column. In the secondary roosts, choice was constrained to blue for the first two sessions. Dark red lines represent choices made by single individuals, and the bright red line represents the roost mean. Trained demonstrators have been removed. The data underlying this figure can be found in our data and code repository (https://doi.org/10.5281/zenodo.19052060).
