# Supplementary material for: Wild parrots exhibit age-dependent conformity when learning about novel food
Source: PLoS Biol. 2026 Apr 30;24(4):e3003741. doi: 10.1371/journal.pbio.3003741 (PMC13132227; doi:10.1371/journal.pbio.3003741)
Supplement: S2 Fig — Each panel represents one individual. The ID, sex, and age of the individual are indicated at the top of each panel. The top row of each graph represents the colour choice of the individual (red or blue) at each foraging bouts. Filled dots represent success, while empty circles represent failures (i.e., individuals dropping the almond with 3 s after picking it up from the dispenser). Coloured lines represent the prediction by each of the considered models. The roost(s) recorded at the top of each graph show at which site(s) an individual solved over the course of the experiment. The data underlying this figure can be found in our data and code repository (https://doi.org/10.5281/zenodo.19052060). (PDF) [file pbio.3003741.s002.pdf]

1

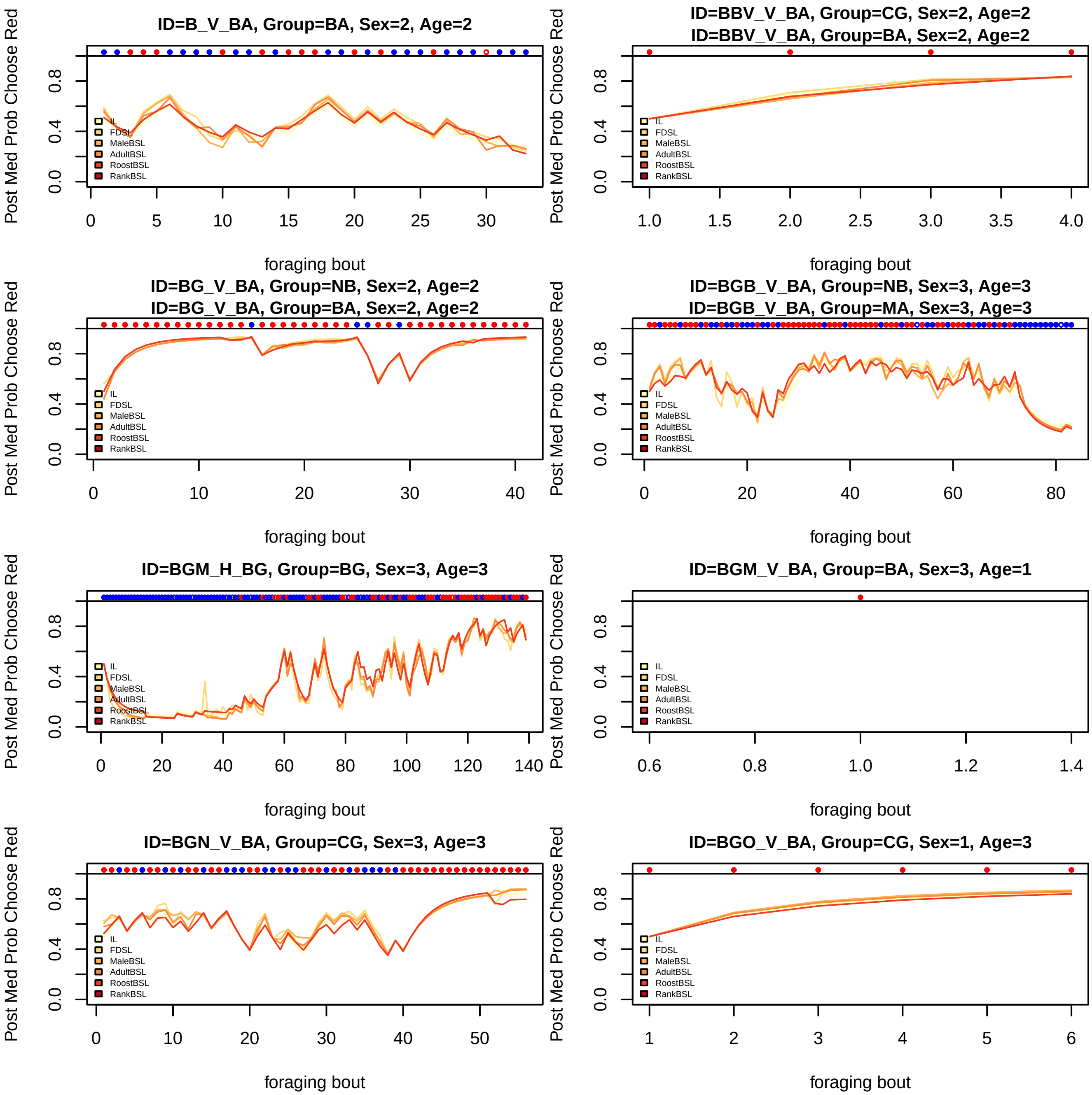

ID=B_V_BA, Group=BA, Sex=2, Age=2
ID=BBV_V_BA, Group=CG, Sex=2, Age=2
ID=BBV_V_BA, Group=BA, Sex=2, Age=2
ID=BG_V_BA, Group=NB, Sex=2, Age=2
ID=BG_V_BA, Group=BA, Sex=2, Age=2
ID=BGB_V_BA, Group=NB, Sex=3, Age=3
ID=BGB_V_BA, Group=MA, Sex=3, Age=3
ID=BGM_H_BG, Group=BG, Sex=3, Age=3
ID=BGM_V_BA, Group=BA, Sex=3, Age=1
ID=BGN_V_BA, Group=CG, Sex=3, Age=3
ID=BGO_V_BA, Group=CG, Sex=1, Age=3
Post Med Prob Choose Red
foraging bout
IL
FDSL
MaleBSL
AdultBSL
RoostBSL
RankBSL

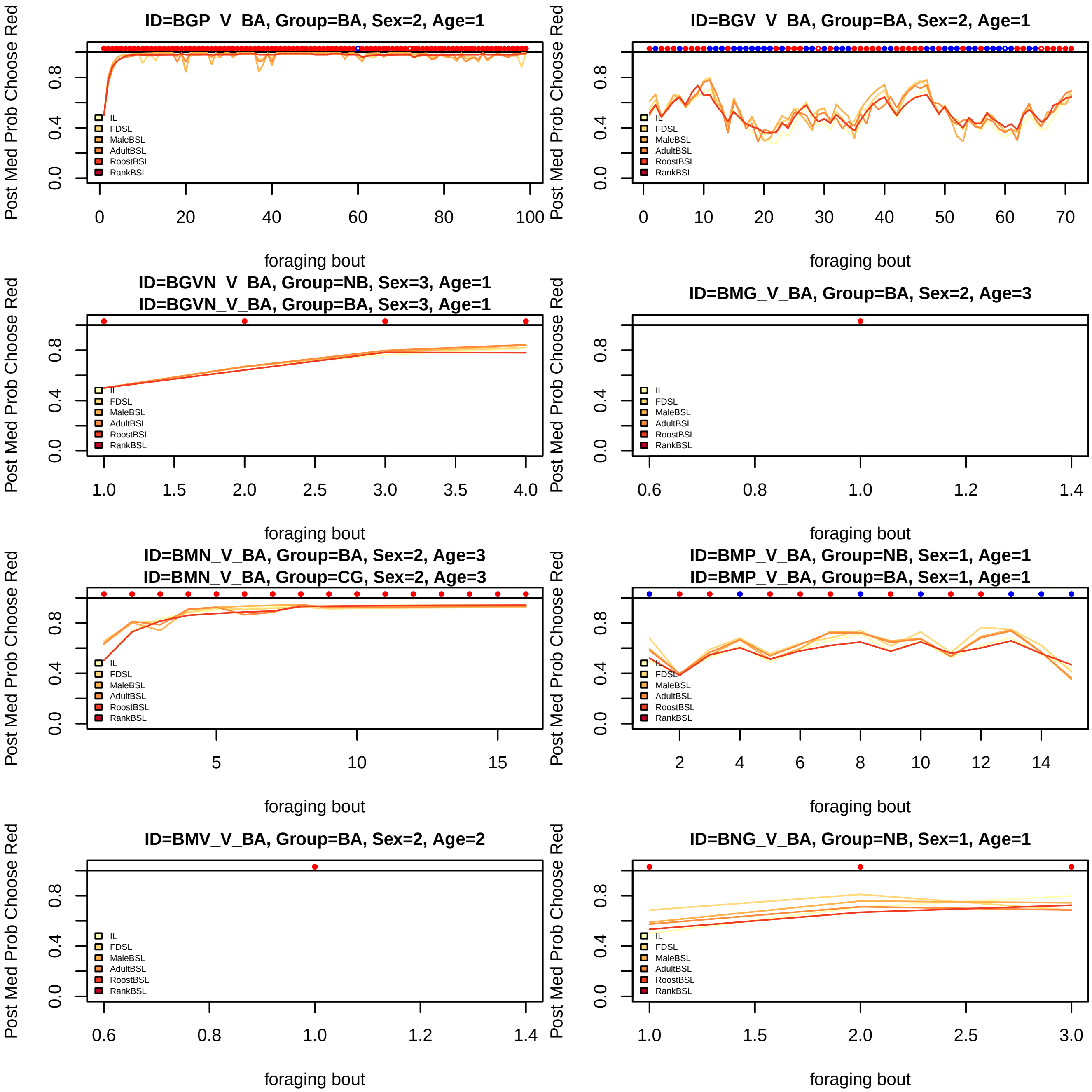
ID=BGP_V_BA, Group=BA, Sex=2, Age=1
ID=BGV_V_BA, Group=BA, Sex=2, Age=1
ID=BGVN_V_BA, Group=NB, Sex=3, Age=1
ID=BGVN_V_BA, Group=BA, Sex=3, Age=1
ID=BMG_V_BA, Group=BA, Sex=2, Age=3
ID=BMN_V_BA, Group=BA, Sex=2, Age=3
ID=BMN_V_BA, Group=CG, Sex=2, Age=3
ID=BMP_V_BA, Group=NB, Sex=1, Age=1
ID=BMP_V_BA, Group=BA, Sex=1, Age=1
ID=BMV_V_BA, Group=BA, Sex=2, Age=2
ID=BNG_V_BA, Group=NB, Sex=1, Age=1
Post Med Prob Choose Red
foraging bout
IL
FDSL
MaleBSL
AdultBSL
RoostBSL
RankBSL

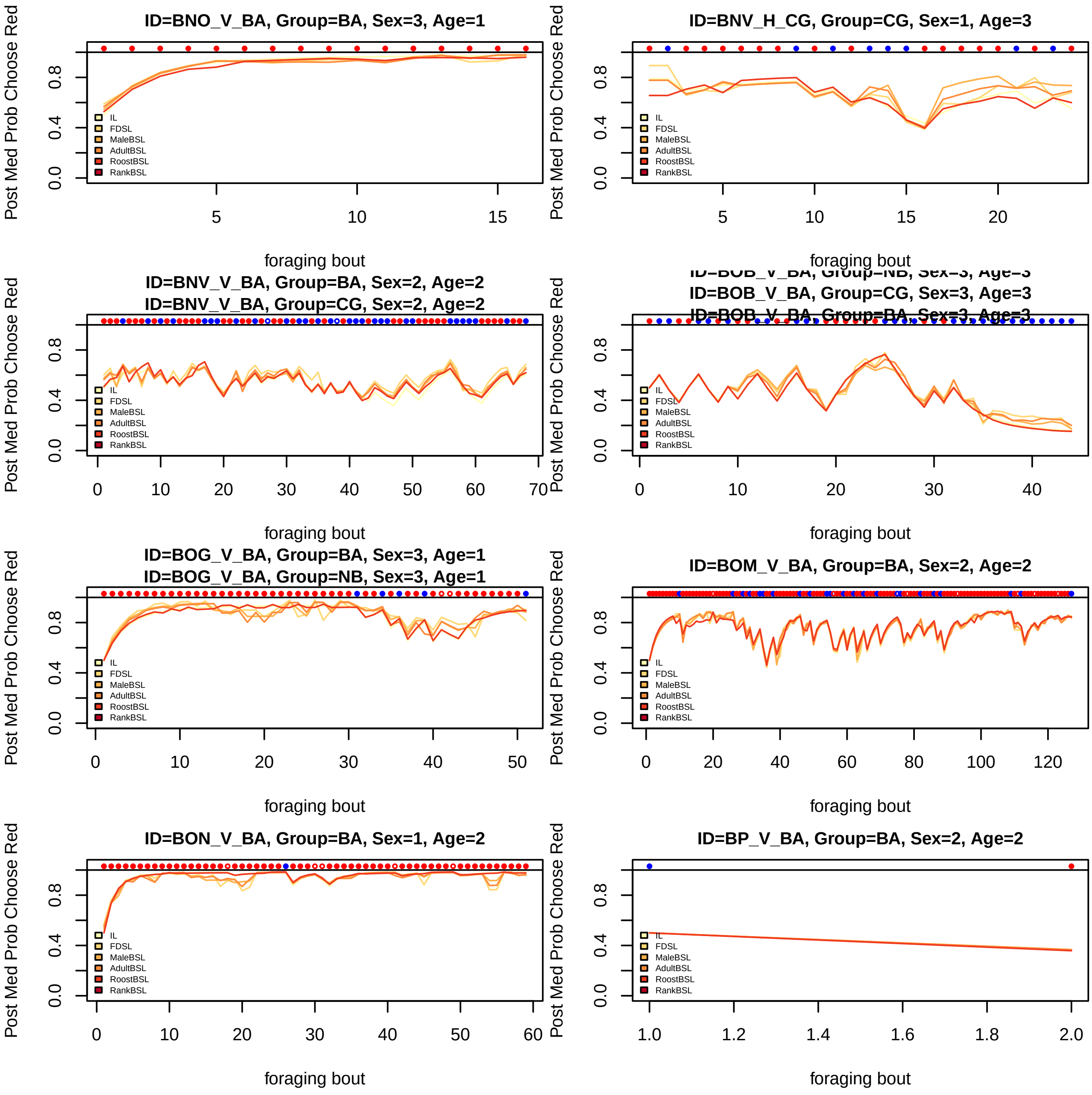
ID=BNO_V_BA, Group=BA, Sex=3, Age=1
ID=BNV_H_CG, Group=CG, Sex=1, Age=3
ID=BNV_V_BA, Group=BA, Sex=2, Age=2
ID=BNV_V_BA, Group=CG, Sex=2, Age=2
ID=BOB_V_BA, Group=CG, Sex=3, Age=3
ID=BOB_V_BA, Group=BA, Sex=3, Age=3
ID=BOG_V_BA, Group=BA, Sex=3, Age=1
ID=BOG_V_BA, Group=NB, Sex=3, Age=1
ID=BOM_V_BA, Group=BA, Sex=2, Age=2
ID=BON_V_BA, Group=BA, Sex=1, Age=2
ID=BP_V_BA, Group=BA, Sex=2, Age=2
Post Med Prob Choose Red
foraging bout
IL
FDSL
MaleBSL
AdultBSL
RoostBSL
RankBSL

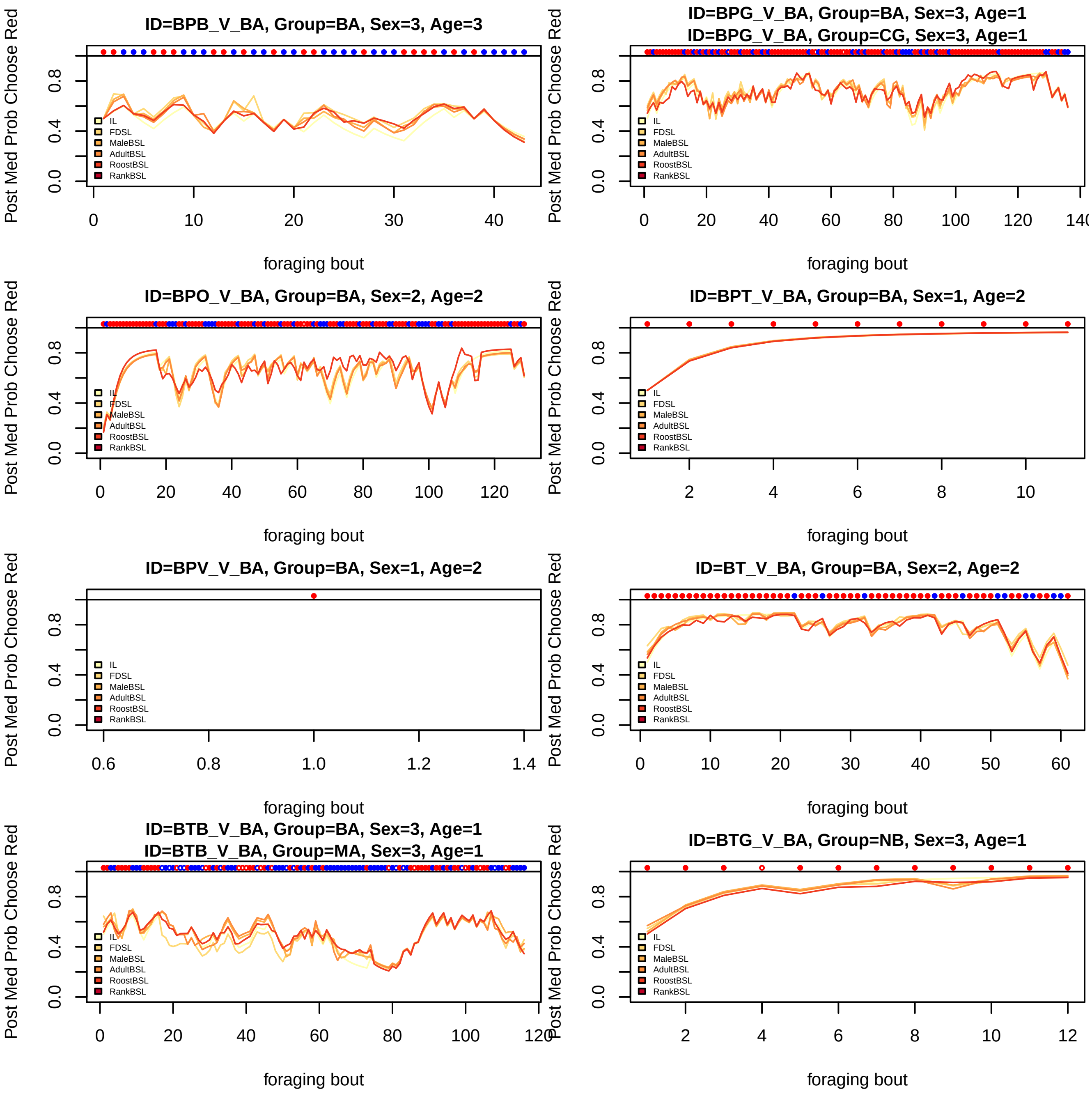
ID=BPB_V_BA, Group=BA, Sex=3, Age=3
ID=BPG_V_BA, Group=BA, Sex=3, Age=1
ID=BPG_V_BA, Group=CG, Sex=3, Age=1
ID=BPO_V_BA, Group=BA, Sex=2, Age=2
ID=BPT_V_BA, Group=BA, Sex=1, Age=2
ID=BPV_V_BA, Group=BA, Sex=1, Age=2
ID=BT_V_BA, Group=BA, Sex=2, Age=2
ID=BTB_V_BA, Group=BA, Sex=3, Age=1
ID=BTB_V_BA, Group=MA, Sex=3, Age=1
ID=BTG_V_BA, Group=NB, Sex=3, Age=1
Post Med Prob Choose Red
foraging bout
IL
FDSL
MaleBSL
AdultBSL
RoostBSL
RankBSL

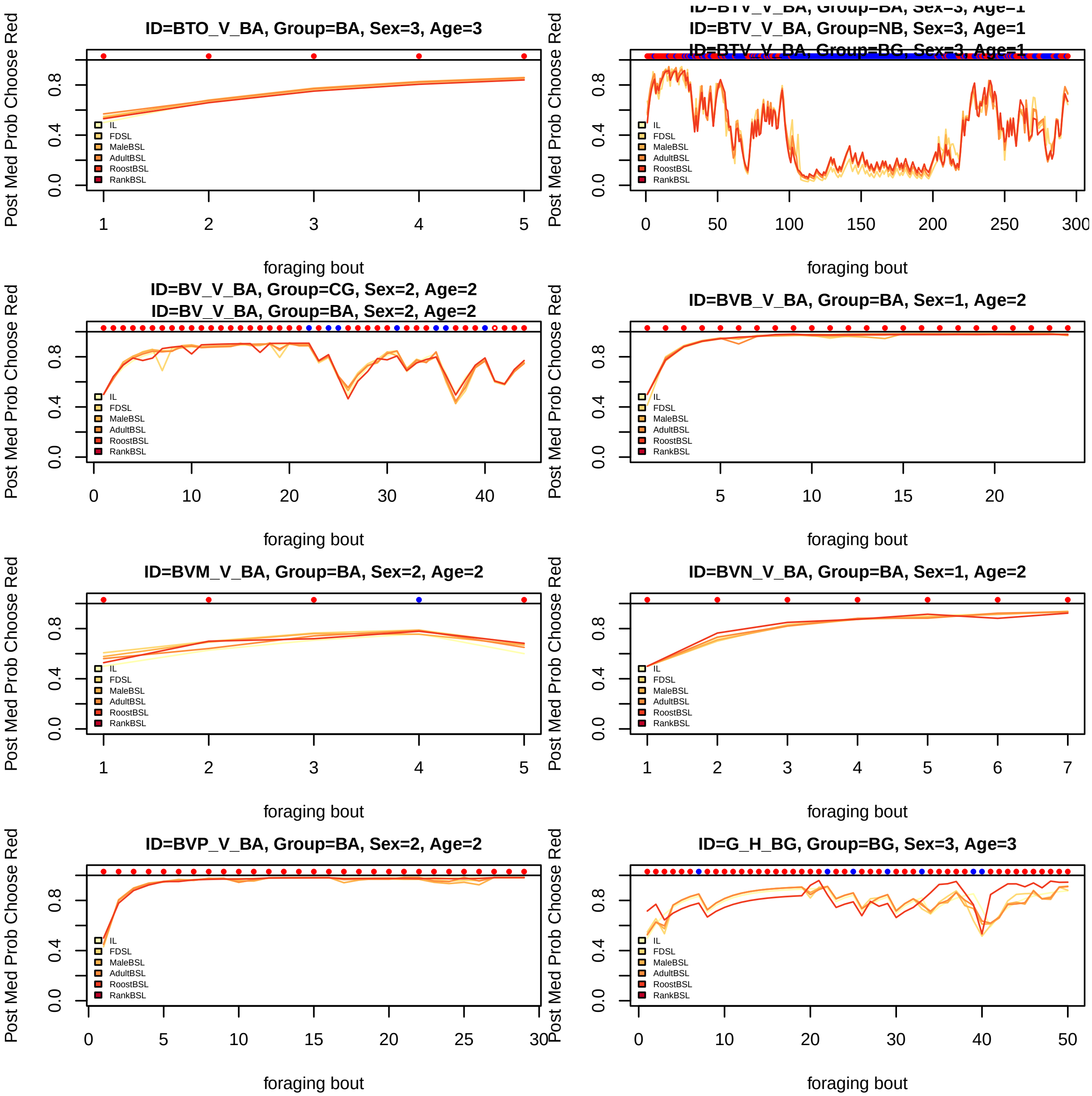
ID=BTO_V_BA, Group=BA, Sex=3, Age=3
ID=BTV_V_BA, Group=NB, Sex=3, Age=1
ID=BTV_V_BA, Group=BG, Sex=3, Age=1
ID=BV_V_BA, Group=CG, Sex=2, Age=2
ID=BV_V_BA, Group=BA, Sex=2, Age=2
ID=BVB_V_BA, Group=BA, Sex=1, Age=2
ID=BVM_V_BA, Group=BA, Sex=2, Age=2
ID=BVN_V_BA, Group=BA, Sex=1, Age=2
ID=BVP_V_BA, Group=BA, Sex=2, Age=2
ID=G_H_BG, Group=BG, Sex=3, Age=3
Post Med Prob Choose Red
foraging bout
IL
FDSL
MaleBSL
AdultBSL
RoostBSL
RankBSL

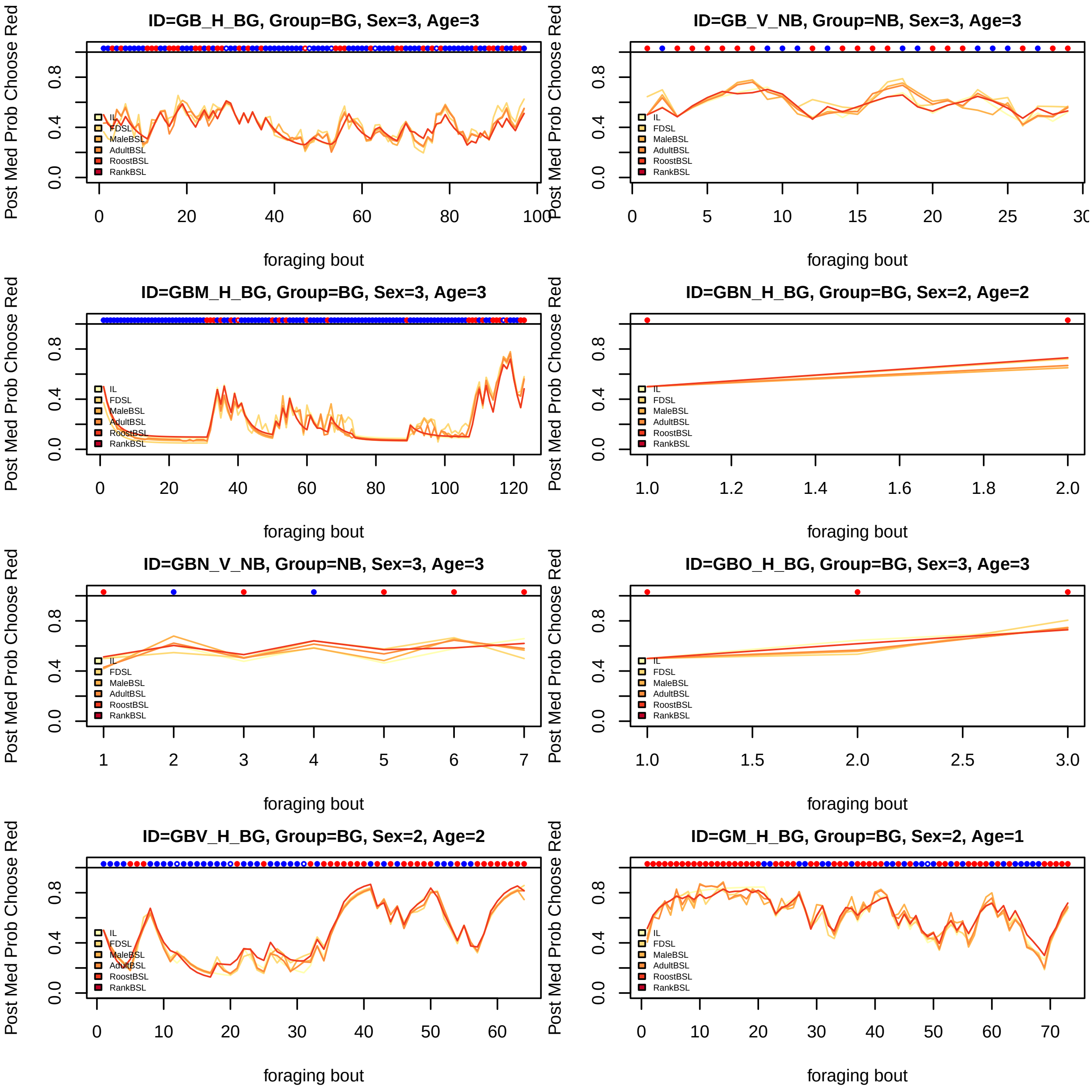
ID=GB_H_BG, Group=BG, Sex=3, Age=3
ID=GB_V_NB, Group=NB, Sex=3, Age=3
ID=GBM_H_BG, Group=BG, Sex=3, Age=3
ID=GBN_H_BG, Group=BG, Sex=2, Age=2
ID=GBN_V_NB, Group=NB, Sex=3, Age=3
ID=GBO_H_BG, Group=BG, Sex=3, Age=3
ID=GBV_H_BG, Group=BG, Sex=2, Age=2
ID=GM_H_BG, Group=BG, Sex=2, Age=1
Post Med Prob Choose Red
foraging bout
IL
FDSL
MaleBSL
AdultBSL
RoostBSL
RankBSL

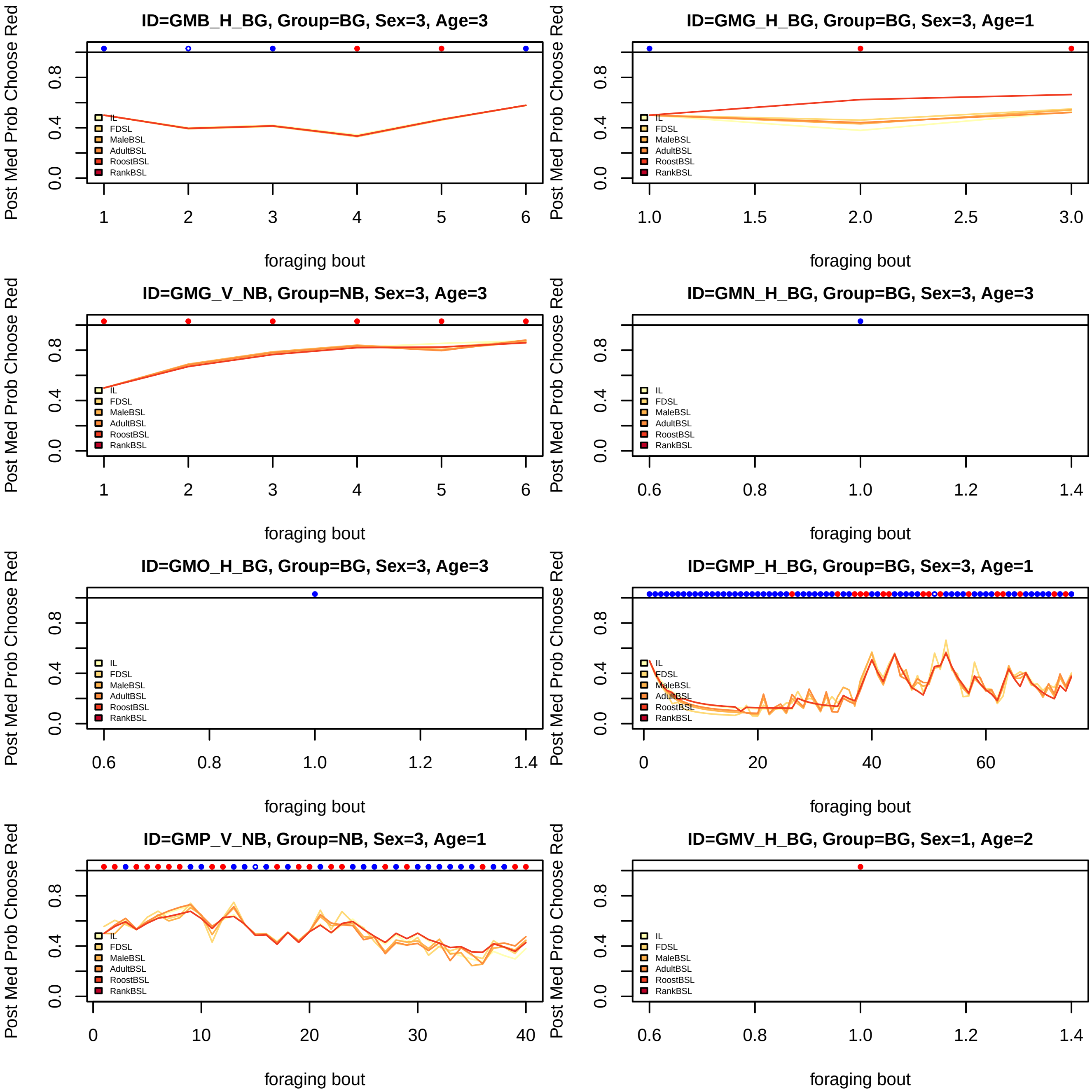
ID=GMB_H_BG, Group=BG, Sex=3, Age=3
ID=GMG_H_BG, Group=BG, Sex=3, Age=1
ID=GMG_V_NB, Group=NB, Sex=3, Age=3
ID=GMN_H_BG, Group=BG, Sex=3, Age=3
ID=GMO_H_BG, Group=BG, Sex=3, Age=3
ID=GMP_H_BG, Group=BG, Sex=3, Age=1
ID=GMP_V_NB, Group=NB, Sex=3, Age=1
ID=GMV_H_BG, Group=BG, Sex=1, Age=2
Post Med Prob Choose Red
foraging bout
IL
FDSL
MaleBSL
AdultBSL
RoostBSL
RankBSL

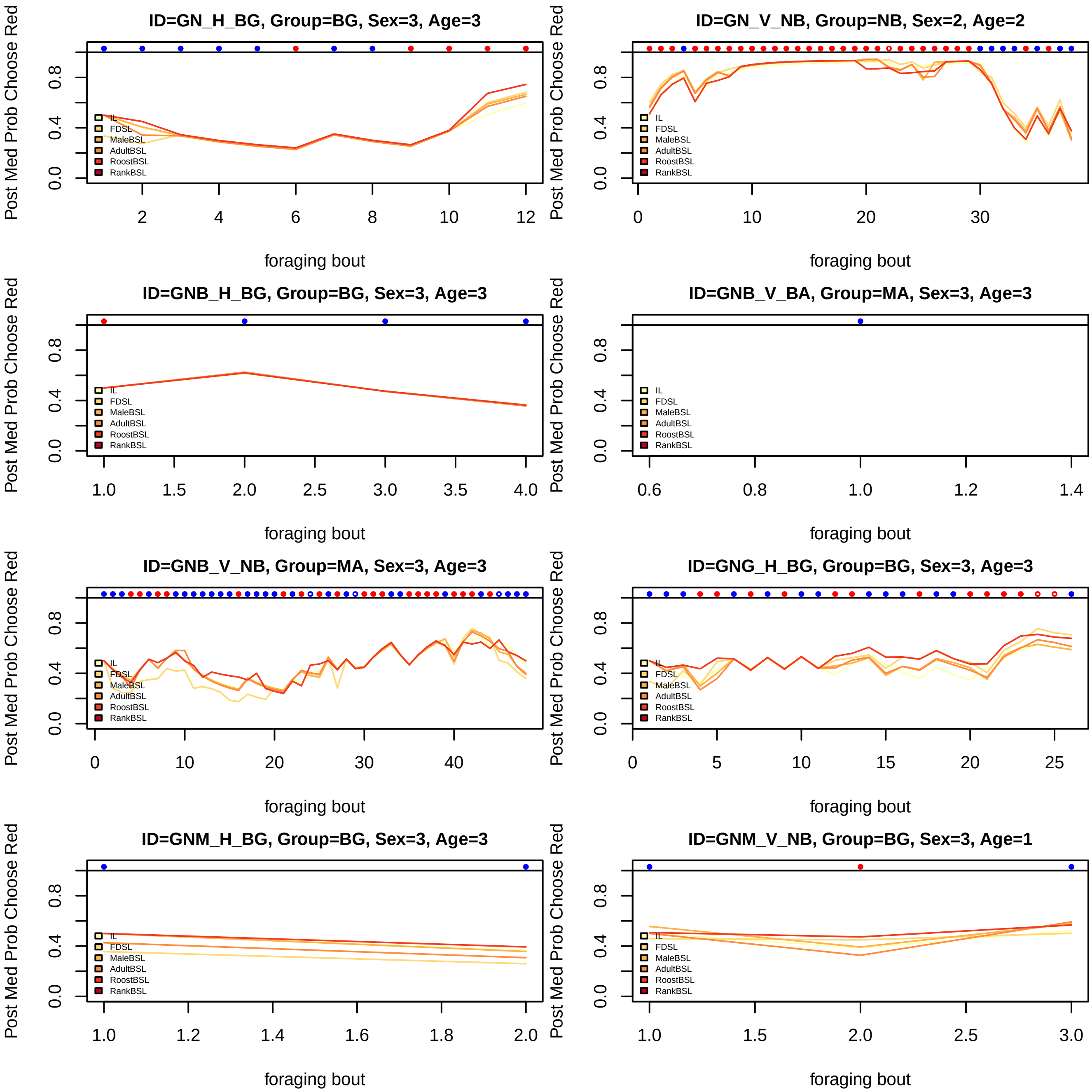
ID=GN_H_BG, Group=BG, Sex=3, Age=3
ID=GN_V_NB, Group=NB, Sex=2, Age=2
ID=GNB_H_BG, Group=BG, Sex=3, Age=3
ID=GNB_V_BA, Group=MA, Sex=3, Age=3
ID=GNB_V_NB, Group=MA, Sex=3, Age=3
ID=GNG_H_BG, Group=BG, Sex=3, Age=3
ID=GNM_H_BG, Group=BG, Sex=3, Age=3
ID=GNM_V_NB, Group=BG, Sex=3, Age=1
Post Med Prob Choose Red
foraging bout
IL
FDSL
MaleBSL
AdultBSL
RoostBSL
RankBSL

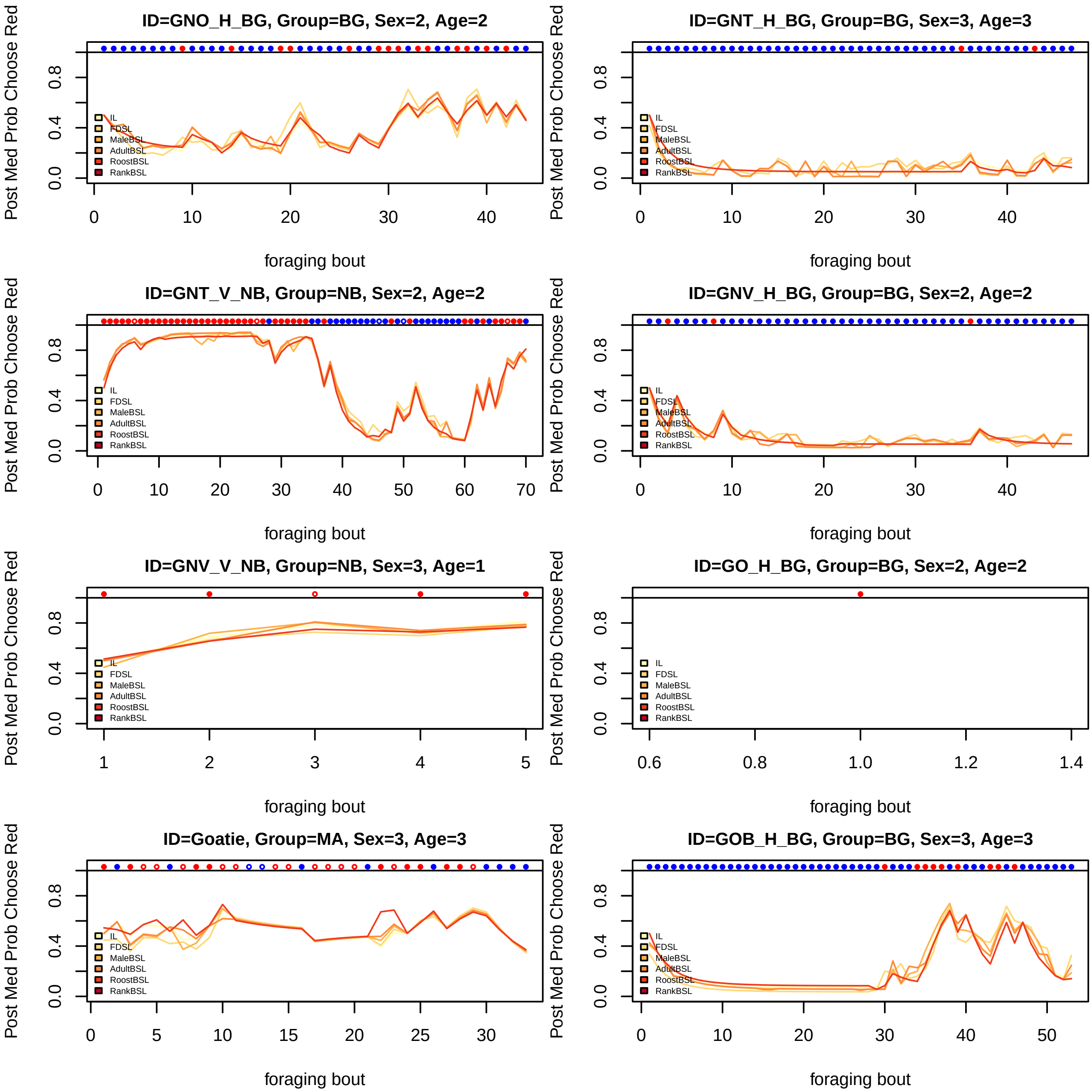

ID=GNO_H_BG, Group=BG, Sex=2, Age=2
ID=GNT_H_BG, Group=BG, Sex=3, Age=3
ID=GNT_V_NB, Group=NB, Sex=2, Age=2
ID=GNV_H_BG, Group=BG, Sex=2, Age=2
ID=GNV_V_NB, Group=NB, Sex=3, Age=1
ID=GO_H_BG, Group=BG, Sex=2, Age=2
ID=Goatie, Group=MA, Sex=3, Age=3
ID=GOB_H_BG, Group=BG, Sex=3, Age=3
Post Med Prob Choose Red
foraging bout
IL
FDSL
MaleBSL
AdultBSL
RoostBSL
RankBSL

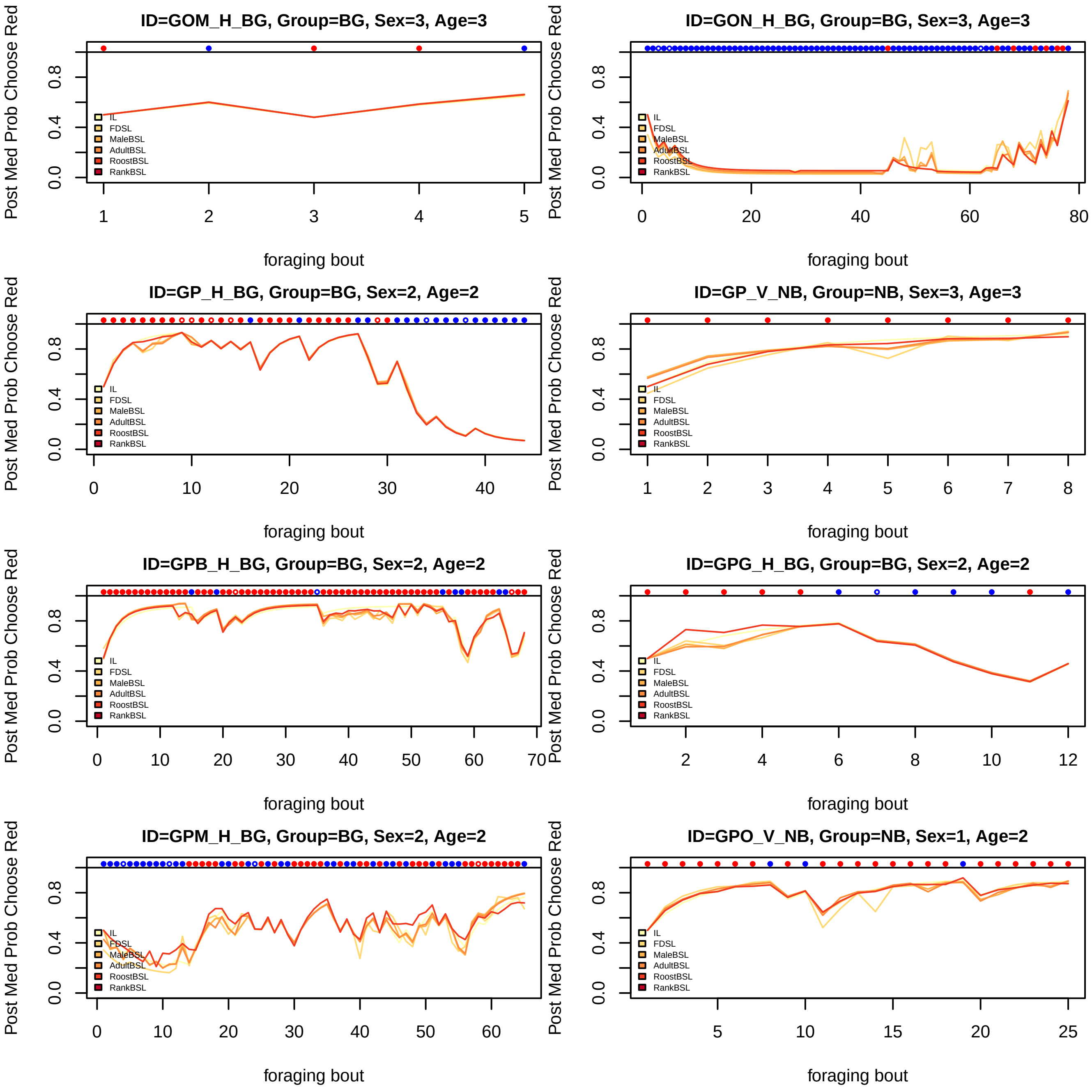
ID=GOM_H_BG, Group=BG, Sex=3, Age=3
ID=GON_H_BG, Group=BG, Sex=3, Age=3
ID=GP_H_BG, Group=BG, Sex=2, Age=2
ID=GP_V_NB, Group=NB, Sex=3, Age=3
ID=GPB_H_BG, Group=BG, Sex=2, Age=2
ID=GPG_H_BG, Group=BG, Sex=2, Age=2
ID=GPM_H_BG, Group=BG, Sex=2, Age=2
ID=GPO_V_NB, Group=NB, Sex=1, Age=2
Post Med Prob Choose Red
foraging bout
IL
FDSL
MaleBSL
AdultBSL
RoostBSL
RankBSL

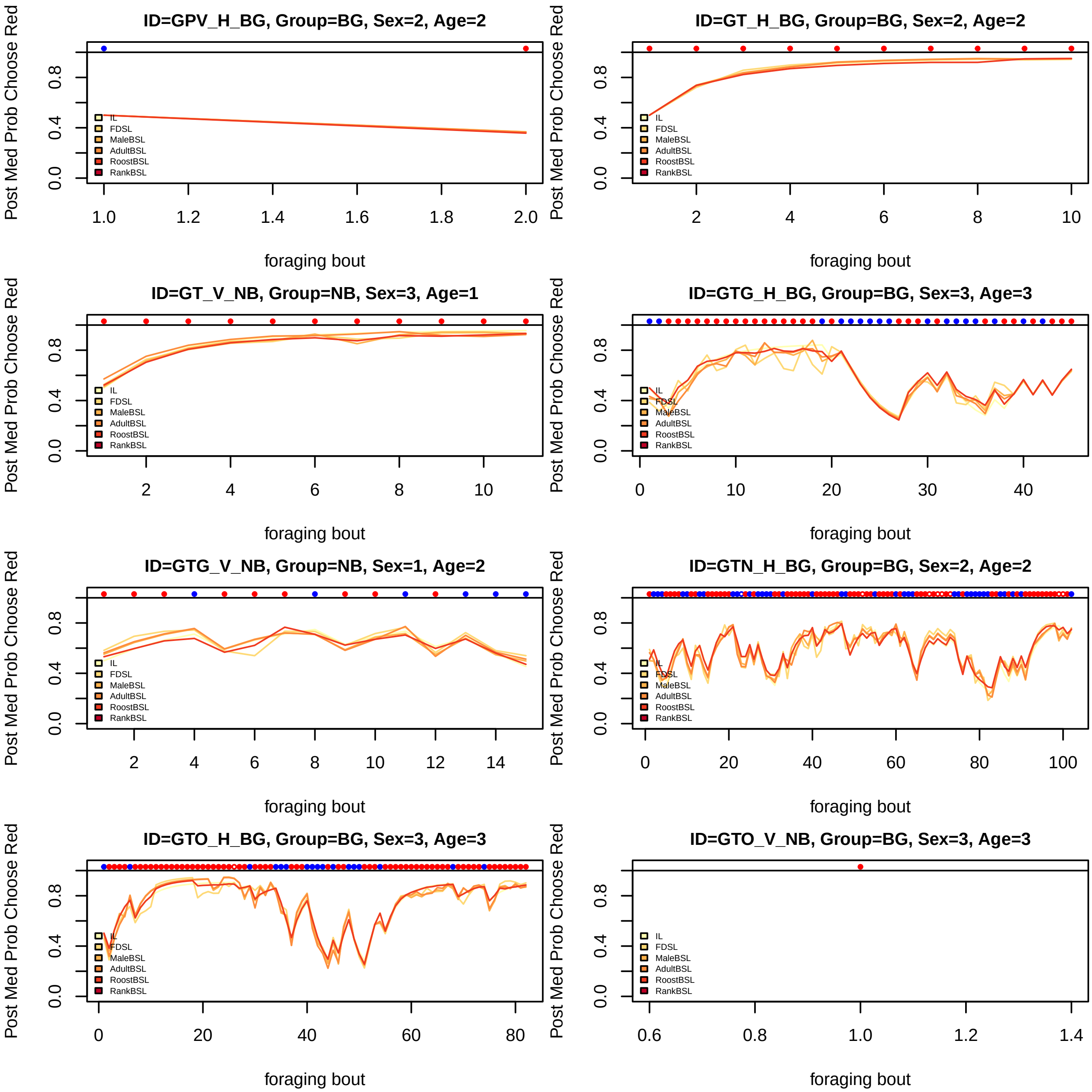
ID=GPV_H_BG, Group=BG, Sex=2, Age=2
ID=GT_H_BG, Group=BG, Sex=2, Age=2
ID=GT_V_NB, Group=NB, Sex=3, Age=1
ID=GTG_H_BG, Group=BG, Sex=3, Age=3
ID=GTG_V_NB, Group=NB, Sex=1, Age=2
ID=GTN_H_BG, Group=BG, Sex=2, Age=2
ID=GTO_H_BG, Group=BG, Sex=3, Age=3
ID=GTO_V_NB, Group=BG, Sex=3, Age=3
Post Med Prob Choose Red
foraging bout
IL
FDSL
MaleBSL
AdultBSL
RoostBSL
RankBSL

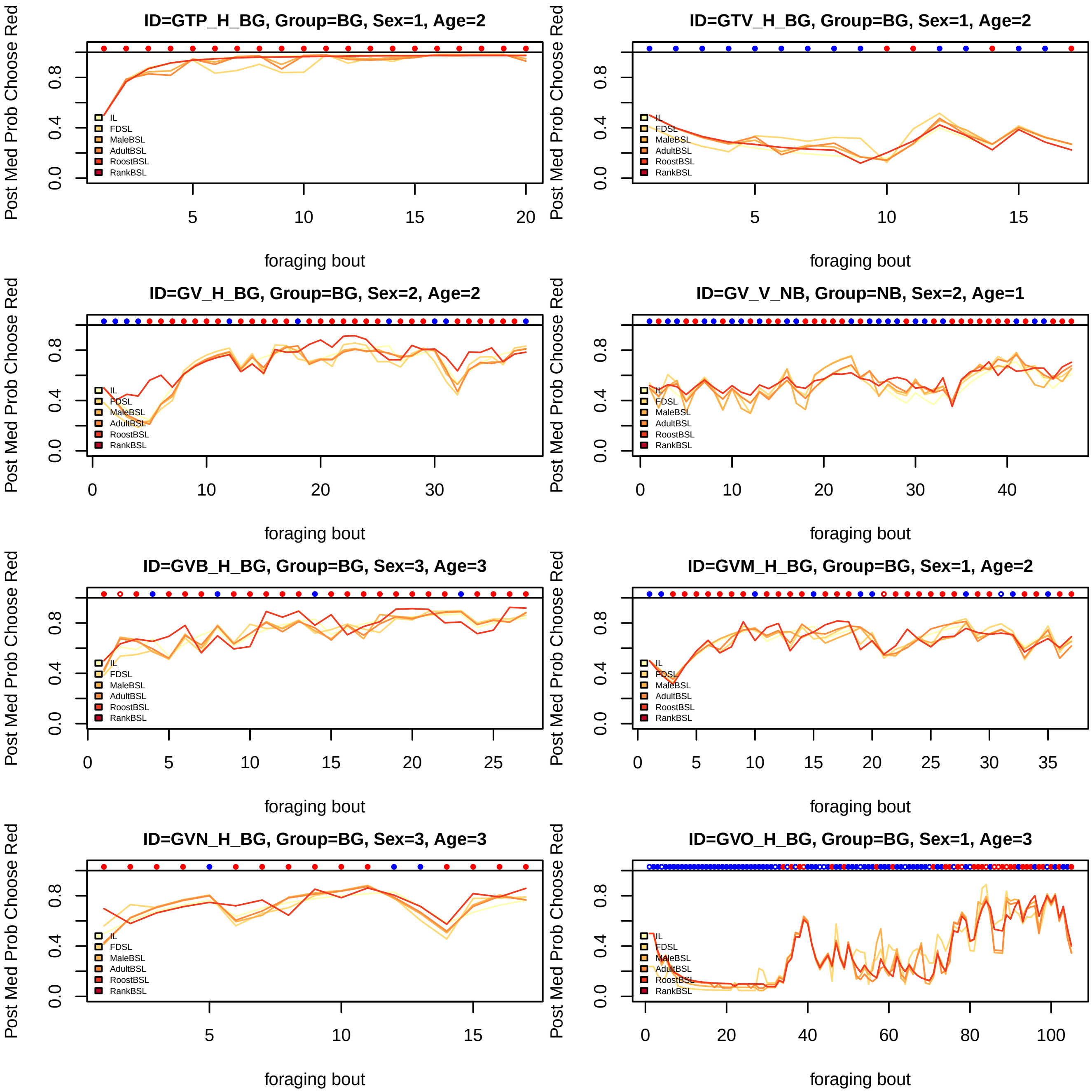
ID=GTP_H_BG, Group=BG, Sex=1, Age=2
ID=GTV_H_BG, Group=BG, Sex=1, Age=2
ID=GV_H_BG, Group=BG, Sex=2, Age=2
ID=GV_V_NB, Group=NB, Sex=2, Age=1
ID=GVB_H_BG, Group=BG, Sex=3, Age=3
ID=GVM_H_BG, Group=BG, Sex=1, Age=2
ID=GVN_H_BG, Group=BG, Sex=3, Age=3
ID=GVO_H_BG, Group=BG, Sex=1, Age=3
Post Med Prob Choose Red
foraging bout
IL
FDSL
MaleBSL
AdultBSL
RoostBSL
RankBSL

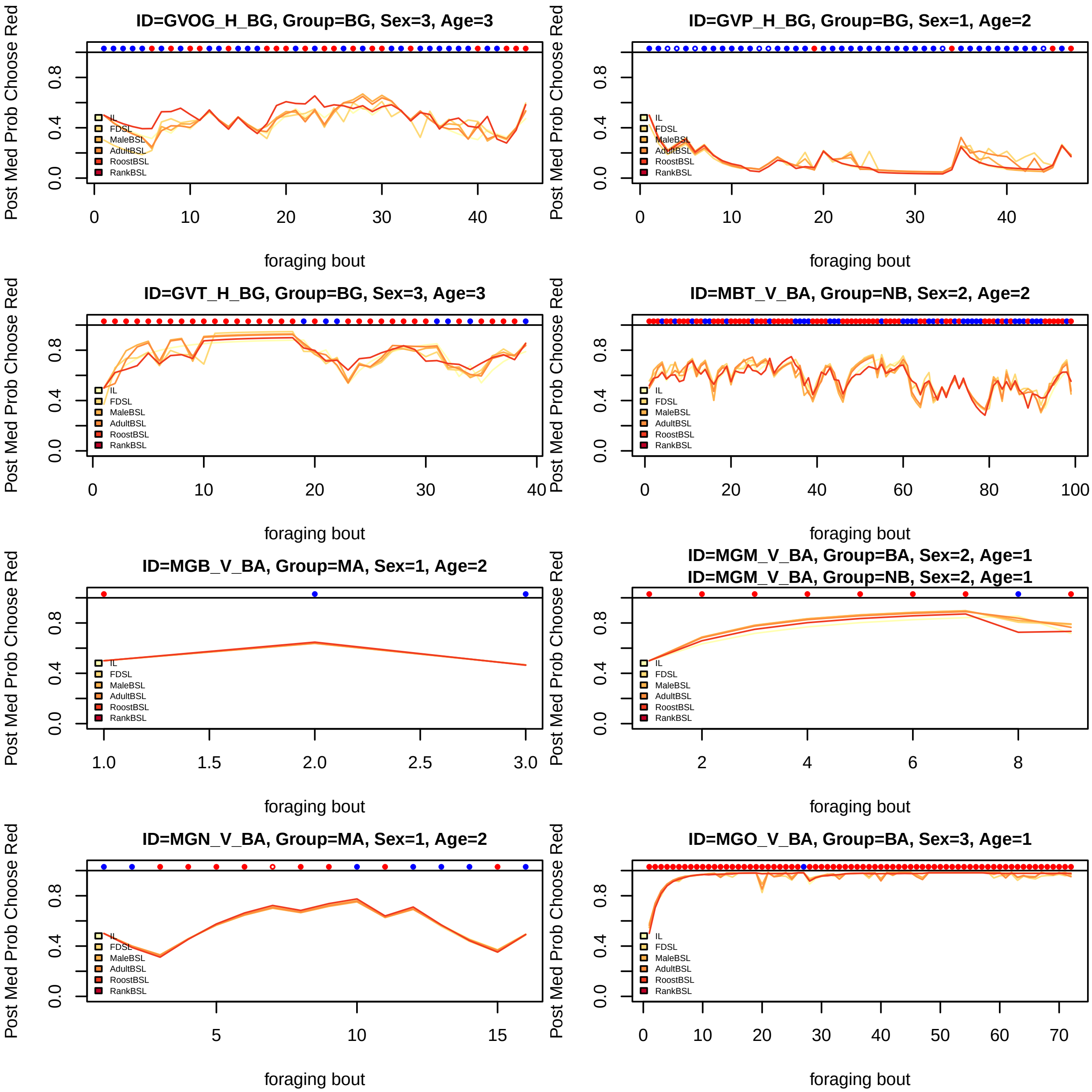
ID=GVOG_H_BG, Group=BG, Sex=3, Age=3
ID=GVP_H_BG, Group=BG, Sex=1, Age=2
ID=GVT_H_BG, Group=BG, Sex=3, Age=3
ID=MBT_V_BA, Group=NB, Sex=2, Age=2
ID=MGB_V_BA, Group=MA, Sex=1, Age=2
ID=MGM_V_BA, Group=BA, Sex=2, Age=1
ID=MGM_V_BA, Group=NB, Sex=2, Age=1
ID=MGN_V_BA, Group=MA, Sex=1, Age=2
ID=MGO_V_BA, Group=BA, Sex=3, Age=1
Post Med Prob Choose Red
foraging bout
IL
FDSL
MaleBSL
AdultBSL
RoostBSL
RankBSL

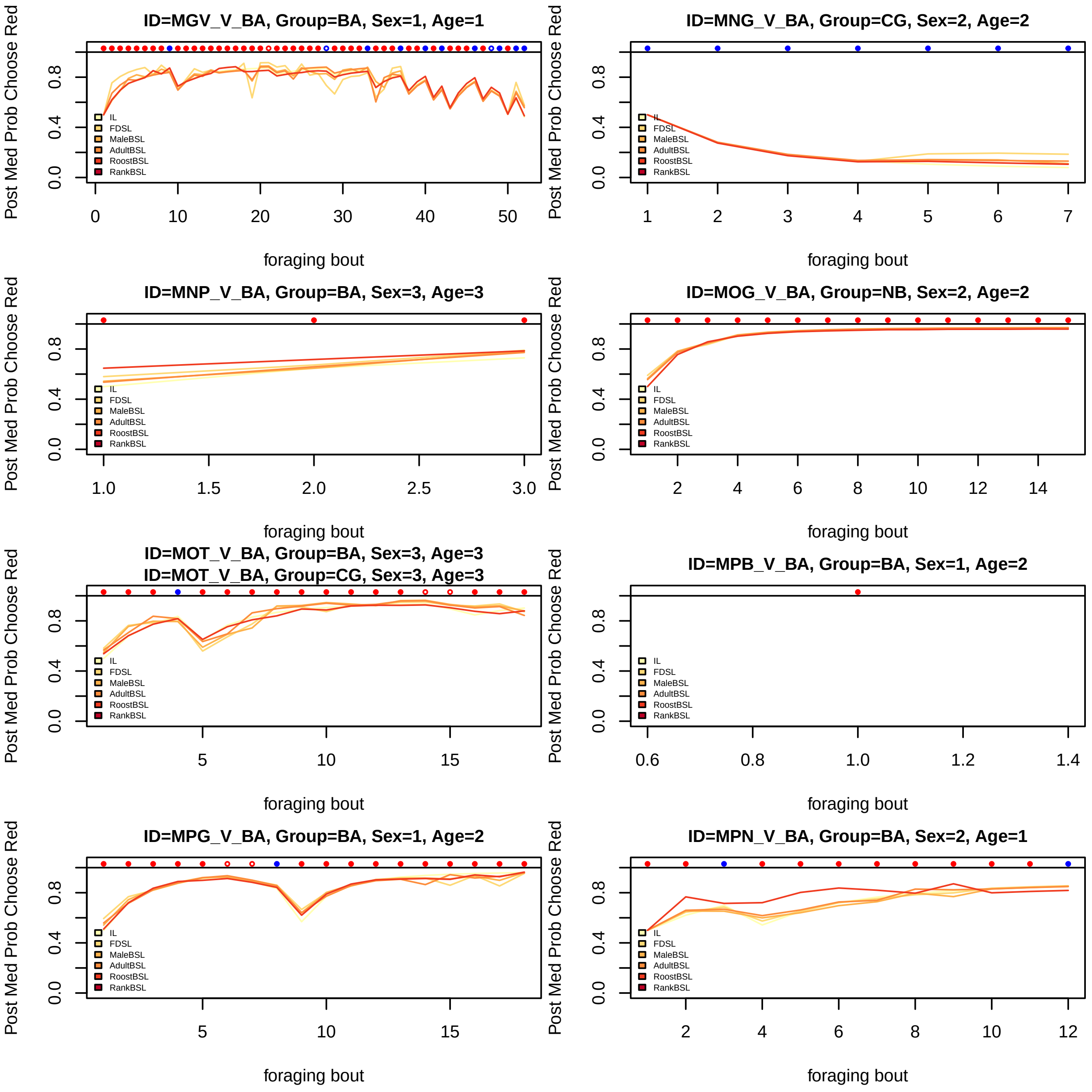
ID=MGV_V_BA, Group=BA, Sex=1, Age=1
ID=MNG_V_BA, Group=CG, Sex=2, Age=2
ID=MNP_V_BA, Group=BA, Sex=3, Age=3
ID=MOG_V_BA, Group=NB, Sex=2, Age=2
ID=MOT_V_BA, Group=BA, Sex=3, Age=3
ID=MOT_V_BA, Group=CG, Sex=3, Age=3
ID=MPB_V_BA, Group=BA, Sex=1, Age=2
ID=MPG_V_BA, Group=BA, Sex=1, Age=2
ID=MPN_V_BA, Group=BA, Sex=2, Age=1
Post Med Prob Choose Red
foraging bout
IL
FDSL
MaleBSL
AdultBSL
RoostBSL
RankBSL

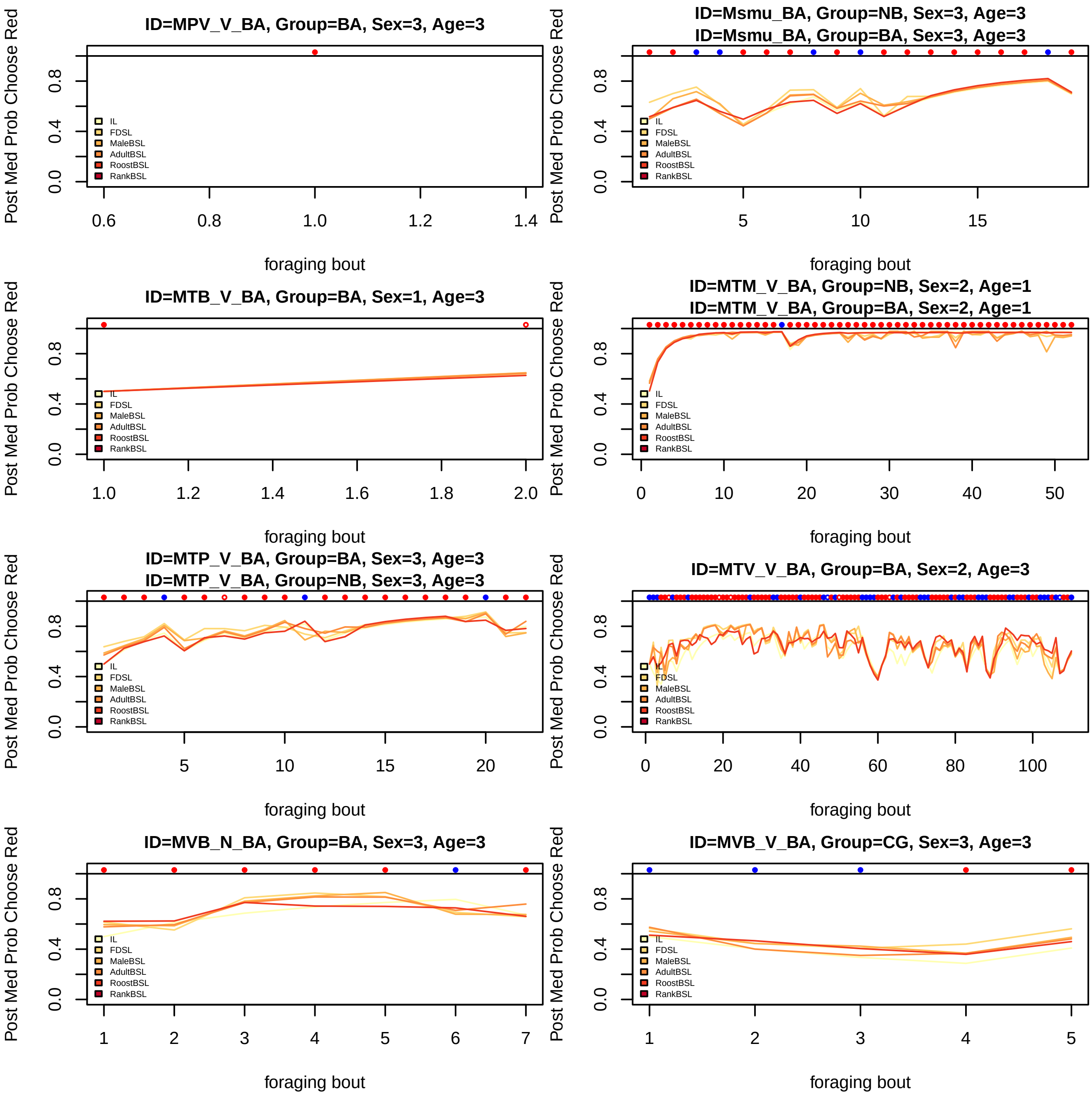
ID=MPV_V_BA, Group=BA, Sex=3, Age=3
ID=Msmu_BA, Group=NB, Sex=3, Age=3
ID=Msmu_BA, Group=BA, Sex=3, Age=3
ID=MTB_V_BA, Group=BA, Sex=1, Age=3
ID=MTM_V_BA, Group=NB, Sex=2, Age=1
ID=MTM_V_BA, Group=BA, Sex=2, Age=1
ID=MTP_V_BA, Group=BA, Sex=3, Age=3
ID=MTP_V_BA, Group=NB, Sex=3, Age=3
ID=MTV_V_BA, Group=BA, Sex=2, Age=3
ID=MVB_N_BA, Group=BA, Sex=3, Age=3
ID=MVB_V_BA, Group=CG, Sex=3, Age=3
Post Med Prob Choose Red
foraging bout
IL
FDSL
MaleBSL
AdultBSL
RoostBSL
RankBSL

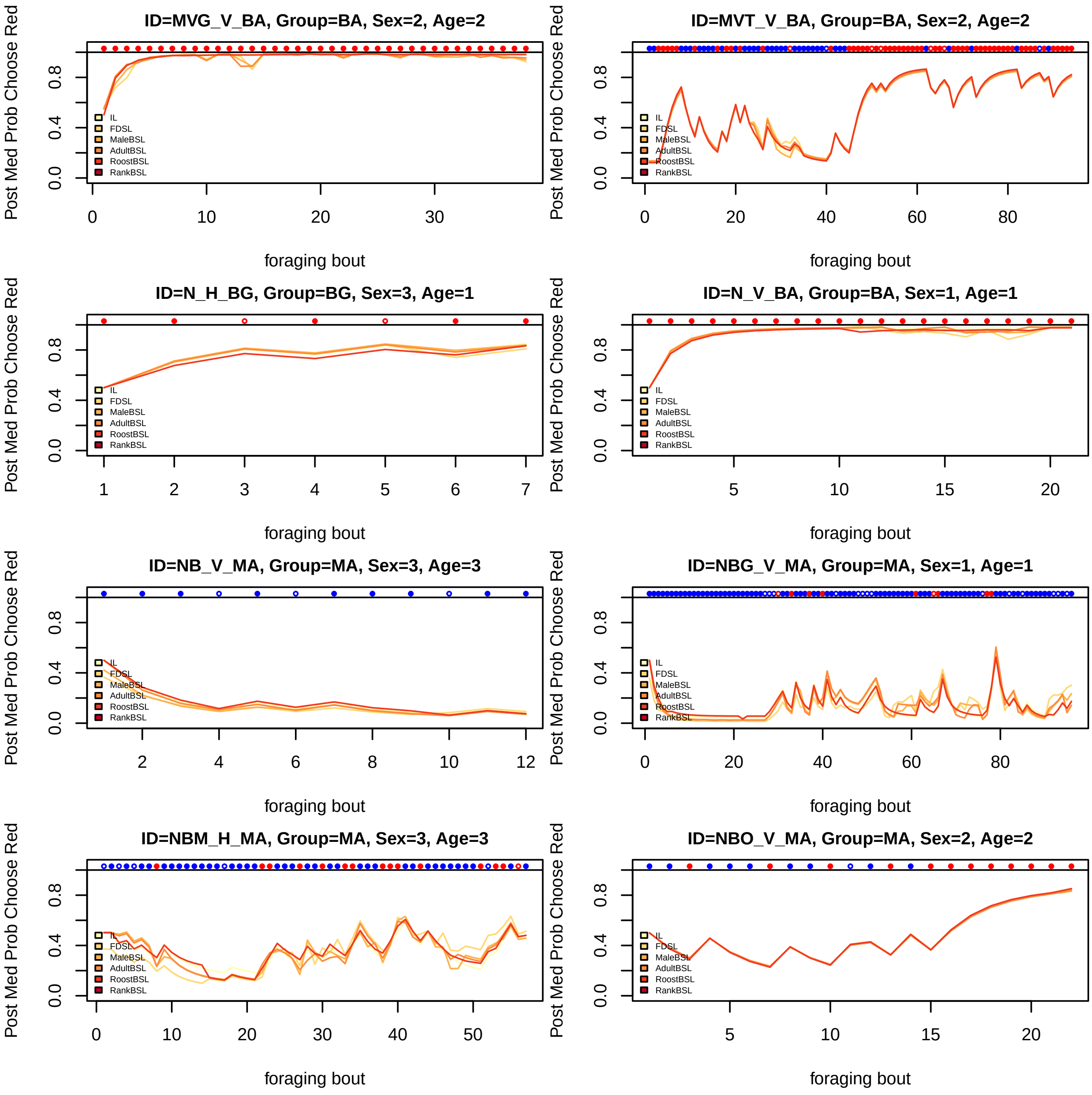
ID=MVG_V_BA, Group=BA, Sex=2, Age=2
ID=MVT_V_BA, Group=BA, Sex=2, Age=2
ID=N_H_BG, Group=BG, Sex=3, Age=1
ID=N_V_BA, Group=BA, Sex=1, Age=1
ID=NB_V_MA, Group=MA, Sex=3, Age=3
ID=NBG_V_MA, Group=MA, Sex=1, Age=1
ID=NBM_H_MA, Group=MA, Sex=3, Age=3
ID=NBO_V_MA, Group=MA, Sex=2, Age=2
Post Med Prob Choose Red
foraging bout
IL
FDSL
MaleBSL
AdultBSL
RoostBSL
RankBSL

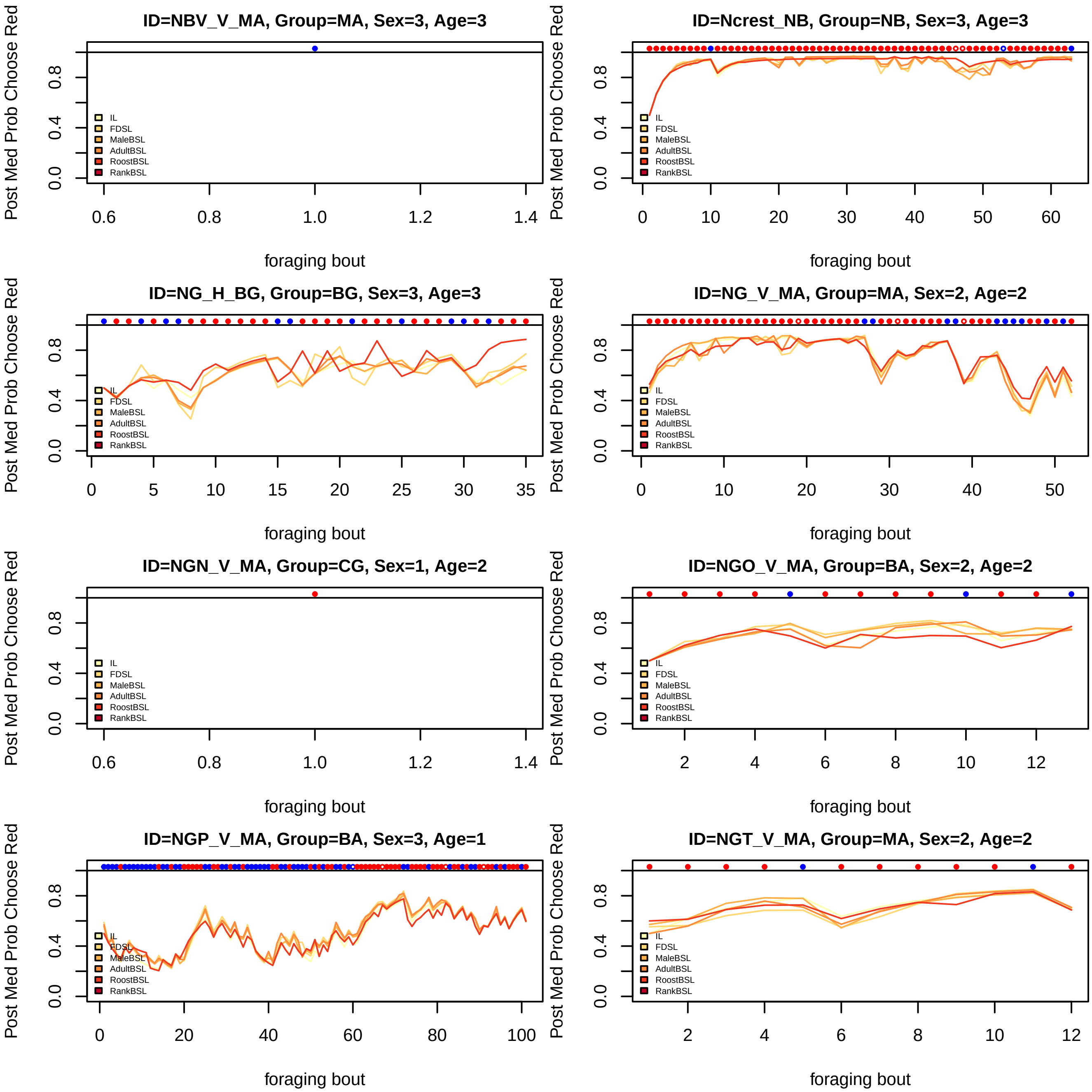
ID=NBV_V_MA, Group=MA, Sex=3, Age=3
ID=Ncrest_NB, Group=NB, Sex=3, Age=3
ID=NG_H_BG, Group=BG, Sex=3, Age=3
ID=NG_V_MA, Group=MA, Sex=2, Age=2
ID=NGN_V_MA, Group=CG, Sex=1, Age=2
ID=NGO_V_MA, Group=BA, Sex=2, Age=2
ID=NGP_V_MA, Group=BA, Sex=3, Age=1
ID=NGT_V_MA, Group=MA, Sex=2, Age=2
Post Med Prob Choose Red
foraging bout
IL
FDSL
MaleBSL
AdultBSL
RoostBSL
RankBSL

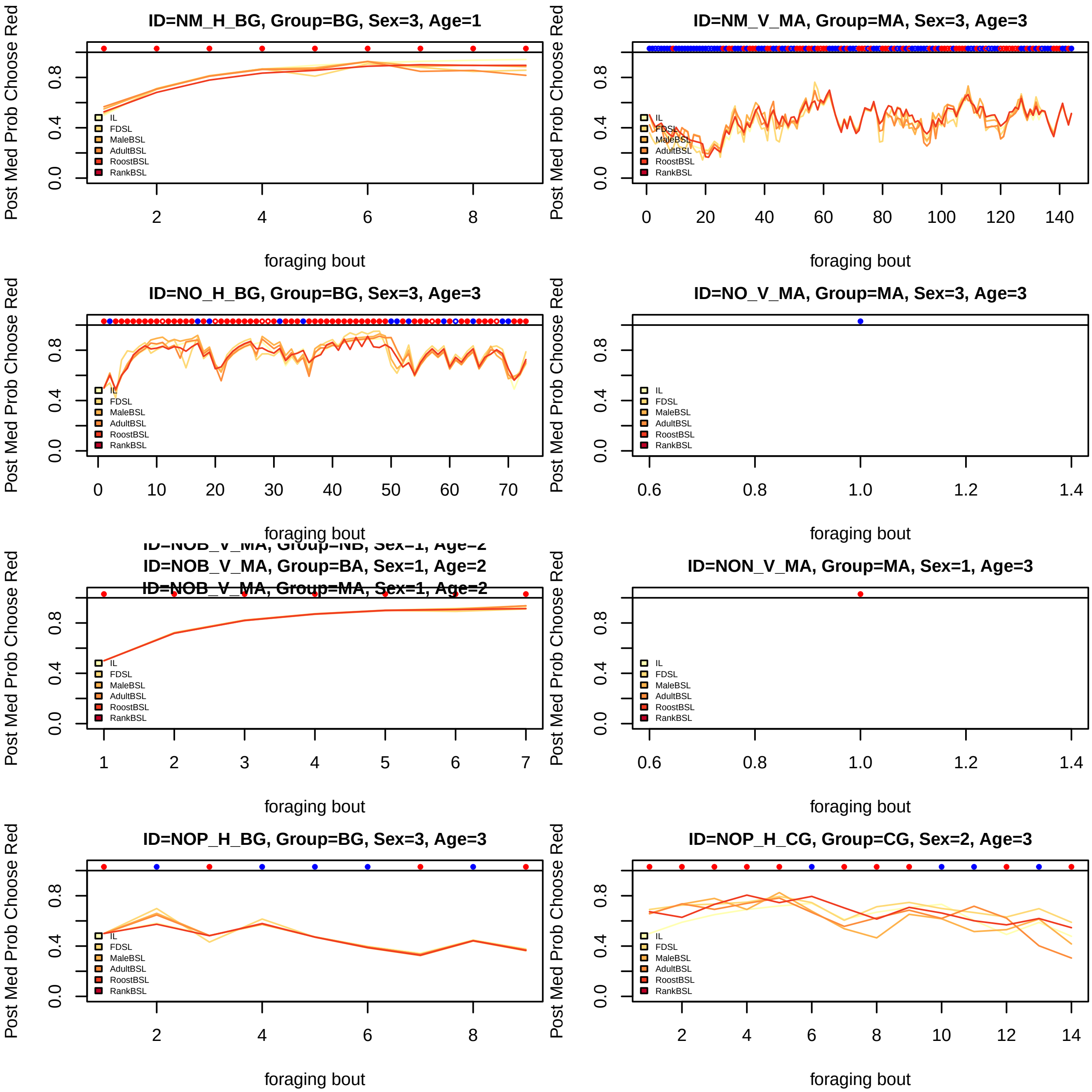
ID=NM_H_BG, Group=BG, Sex=3, Age=1
ID=NM_V_MA, Group=MA, Sex=3, Age=3
ID=NO_H_BG, Group=BG, Sex=3, Age=3
ID=NO_V_MA, Group=MA, Sex=3, Age=3
ID=NOB_V_MA, Group=BA, Sex=1, Age=2
ID=NOB_V_MA, Group=MA, Sex=1, Age=2
ID=NON_V_MA, Group=MA, Sex=1, Age=3
ID=NOP_H_BG, Group=BG, Sex=3, Age=3
ID=NOP_H_CG, Group=CG, Sex=2, Age=3
Post Med Prob Choose Red
foraging bout
IL
FDSL
MaleBSL
AdultBSL
RoostBSL
RankBSL

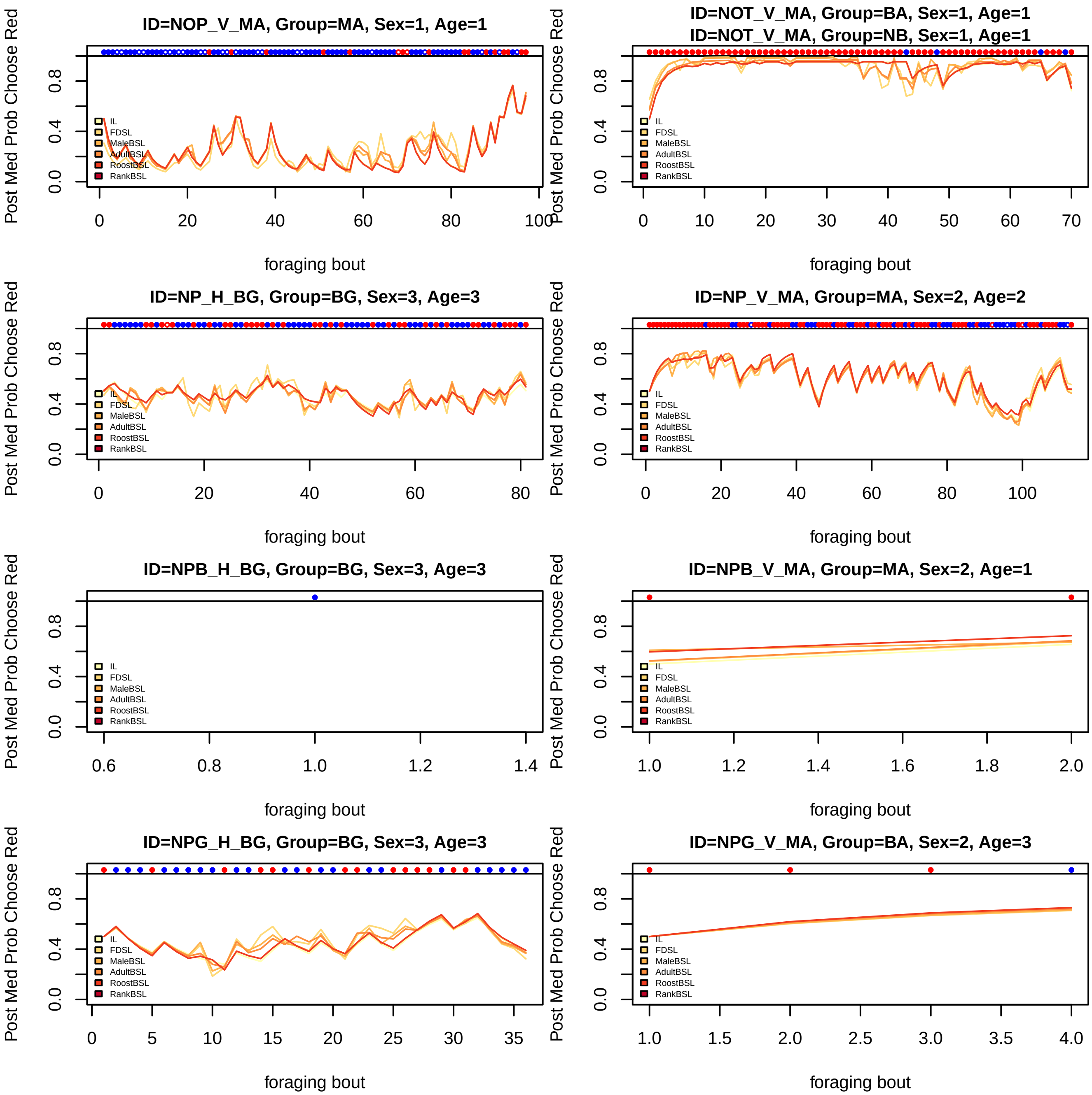
ID=NOP_V_MA, Group=MA, Sex=1, Age=1
ID=NOT_V_MA, Group=BA, Sex=1, Age=1
ID=NOT_V_MA, Group=NB, Sex=1, Age=1
ID=NP_H_BG, Group=BG, Sex=3, Age=3
ID=NP_V_MA, Group=MA, Sex=2, Age=2
ID=NPB_H_BG, Group=BG, Sex=3, Age=3
ID=NPB_V_MA, Group=MA, Sex=2, Age=1
ID=NPG_H_BG, Group=BG, Sex=3, Age=3
ID=NPG_V_MA, Group=BA, Sex=2, Age=3
Post Med Prob Choose Red
foraging bout
IL
FDSL
MaleBSL
AdultBSL
RoostBSL
RankBSL

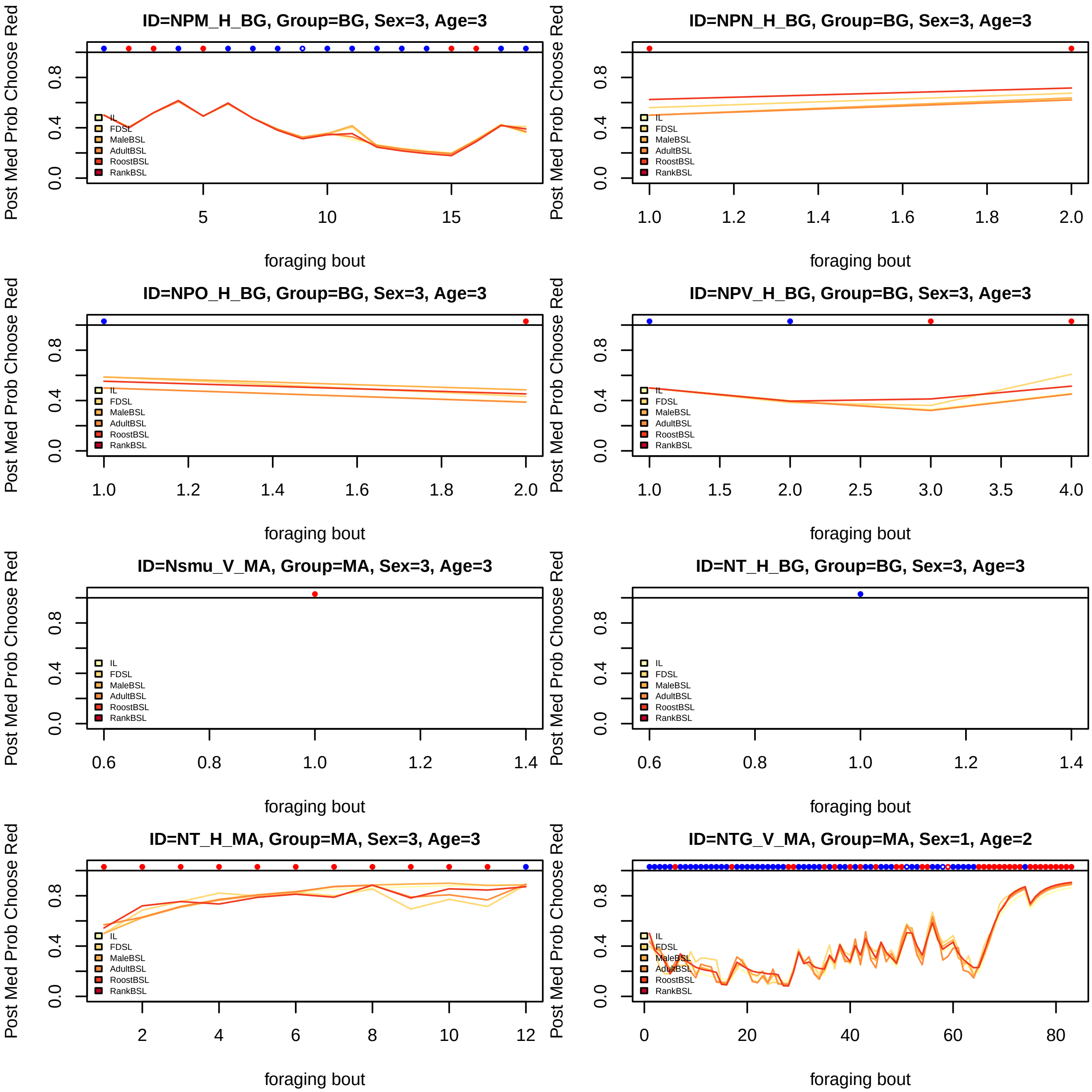
ID=NPM_H_BG, Group=BG, Sex=3, Age=3
ID=NPN_H_BG, Group=BG, Sex=3, Age=3
ID=NPO_H_BG, Group=BG, Sex=3, Age=3
ID=NPV_H_BG, Group=BG, Sex=3, Age=3
ID=Nsmu_V_MA, Group=MA, Sex=3, Age=3
ID=NT_H_BG, Group=BG, Sex=3, Age=3
ID=NT_H_MA, Group=MA, Sex=3, Age=3
ID=NTG_V_MA, Group=MA, Sex=1, Age=2
Post Med Prob Choose Red
foraging bout
IL
FDSL
MaleBSL
AdultBSL
RoostBSL
RankBSL

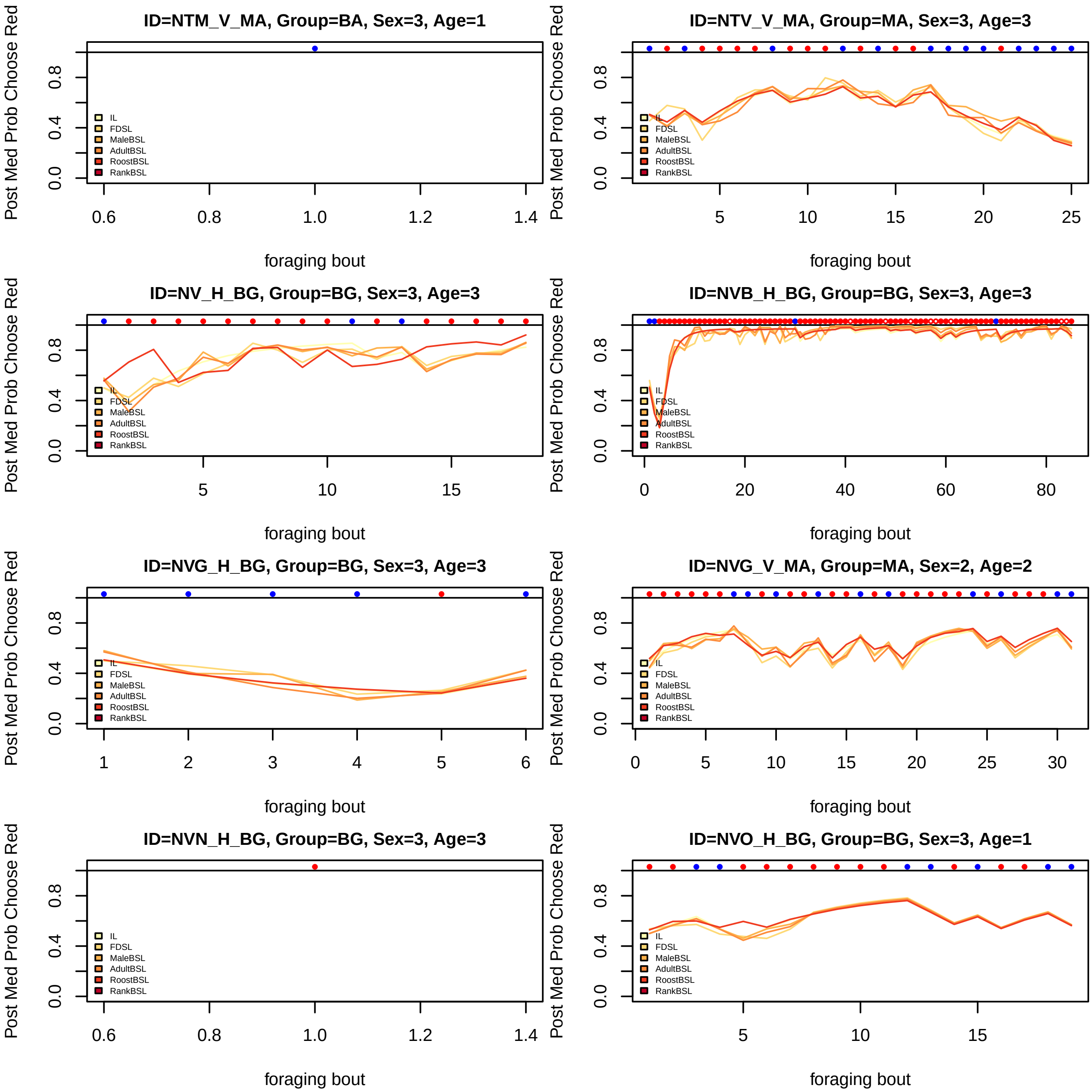

ID=NTM_V_MA, Group=BA, Sex=3, Age=1
ID=NTV_V_MA, Group=MA, Sex=3, Age=3
ID=NV_H_BG, Group=BG, Sex=3, Age=3
ID=NVB_H_BG, Group=BG, Sex=3, Age=3
ID=NVG_H_BG, Group=BG, Sex=3, Age=3
ID=NVG_V_MA, Group=MA, Sex=2, Age=2
ID=NVN_H_BG, Group=BG, Sex=3, Age=3
ID=NVO_H_BG, Group=BG, Sex=3, Age=1
Post Med Prob Choose Red
foraging bout
IL
FDSL
MaleBSL
AdultBSL
RoostBSL
RankBSL

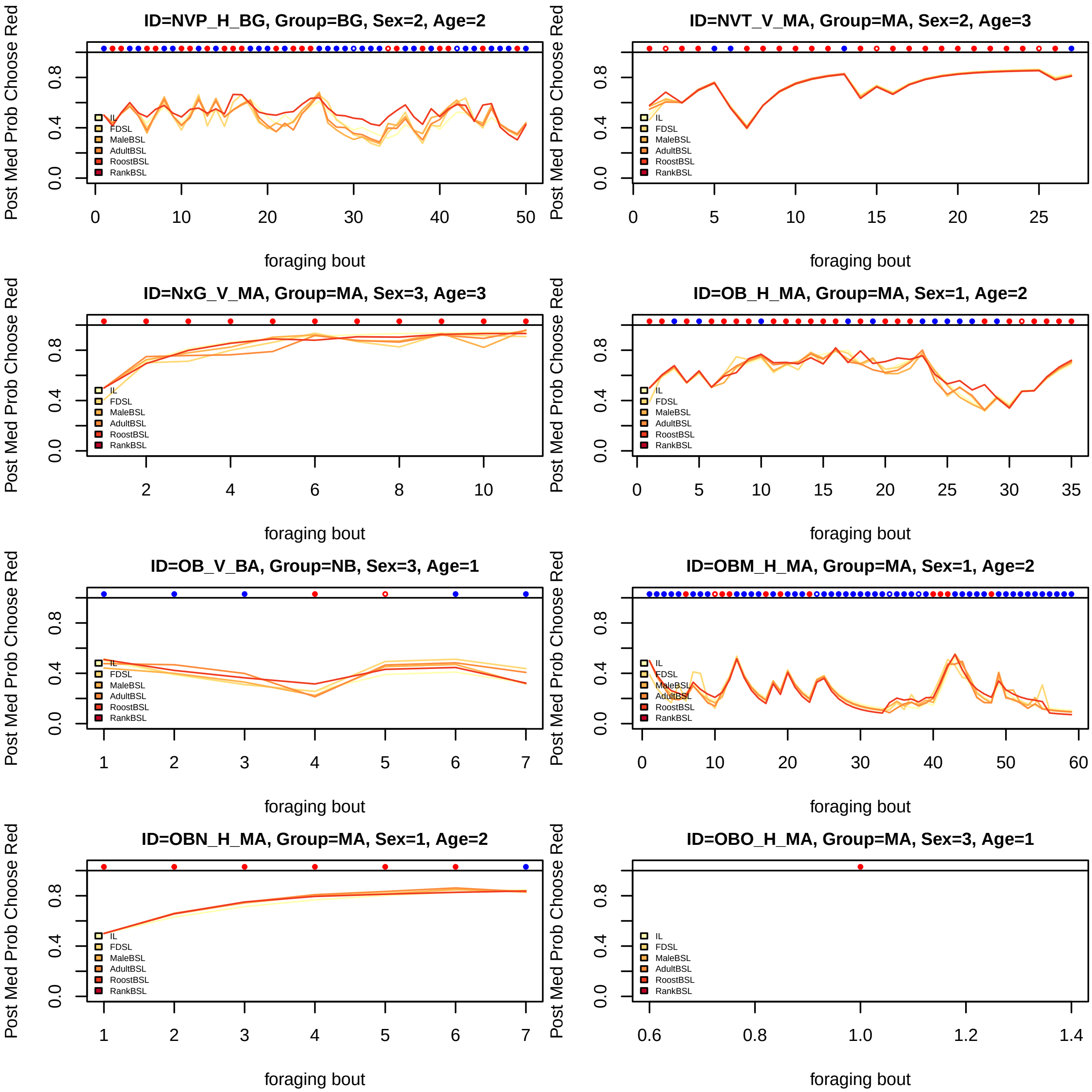
ID=NVP_H_BG, Group=BG, Sex=2, Age=2
ID=NVT_V_MA, Group=MA, Sex=2, Age=3
ID=NxG_V_MA, Group=MA, Sex=3, Age=3
ID=OB_H_MA, Group=MA, Sex=1, Age=2
ID=OB_V_BA, Group=NB, Sex=3, Age=1
ID=OBM_H_MA, Group=MA, Sex=1, Age=2
ID=OBN_H_MA, Group=MA, Sex=1, Age=2
ID=OBO_H_MA, Group=MA, Sex=3, Age=1
Post Med Prob Choose Red
foraging bout
IL
FDSL
MaleBSL
AdultBSL
RoostBSL
RankBSL

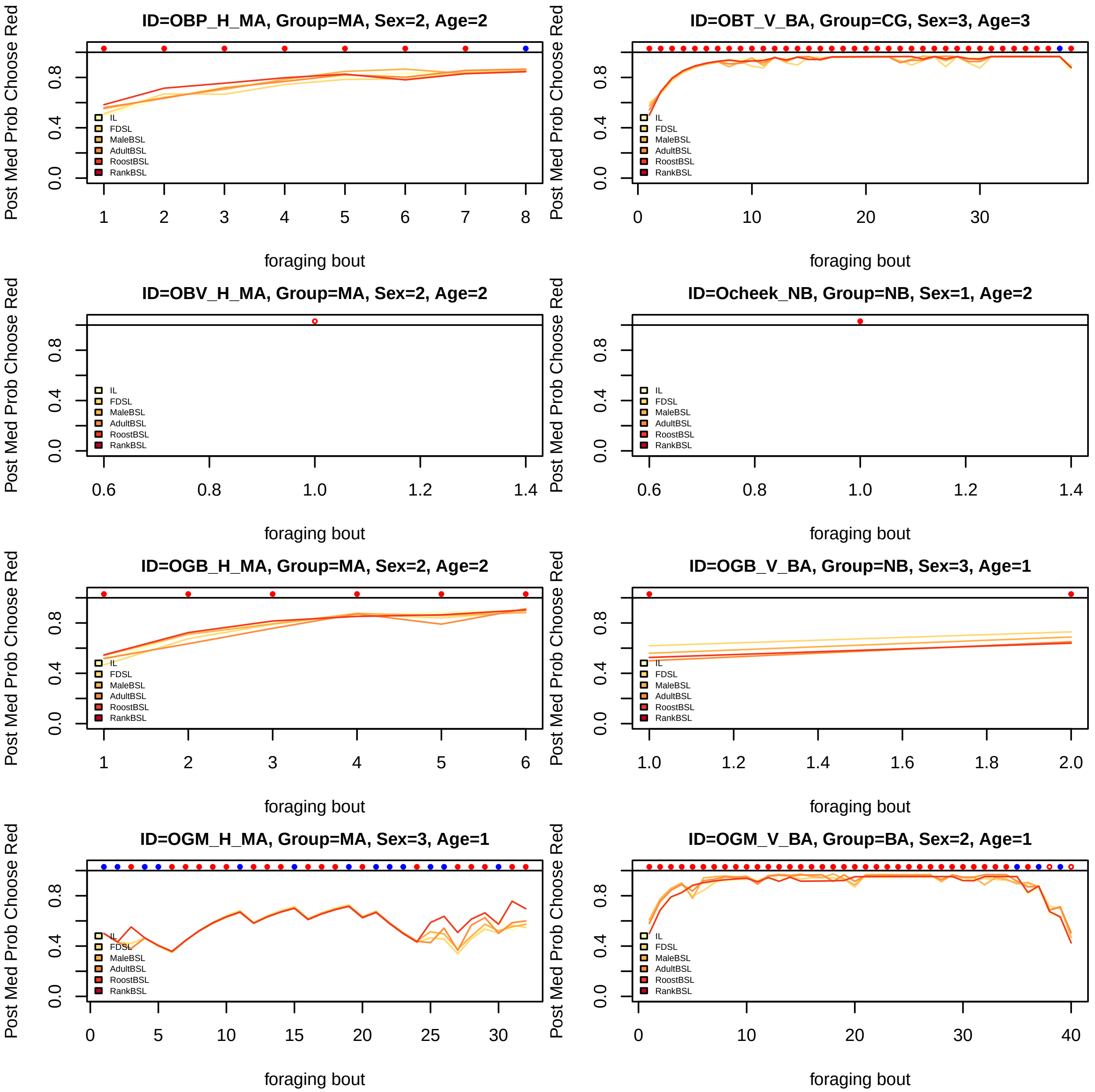
ID=OBP_H_MA, Group=MA, Sex=2, Age=2
ID=OBT_V_BA, Group=CG, Sex=3, Age=3
ID=OBV_H_MA, Group=MA, Sex=2, Age=2
ID=Ocheek_NB, Group=NB, Sex=1, Age=2
ID=OGB_H_MA, Group=MA, Sex=2, Age=2
ID=OGB_V_BA, Group=NB, Sex=3, Age=1
ID=OGM_H_MA, Group=MA, Sex=3, Age=1
ID=OGM_V_BA, Group=BA, Sex=2, Age=1
Post Med Prob Choose Red
foraging bout
IL
FDSL
MaleBSL
AdultBSL
RoostBSL
RankBSL

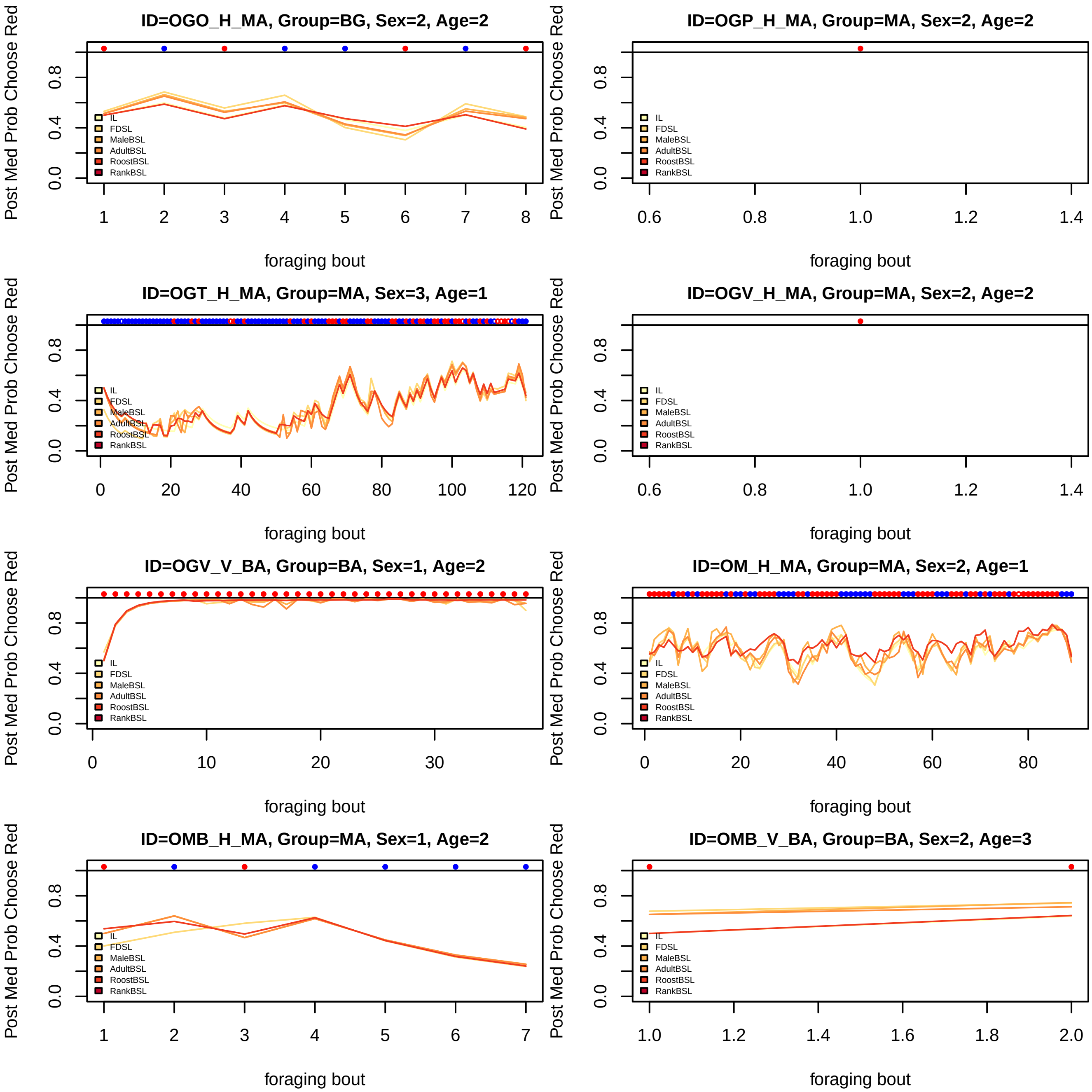
ID=OGO_H_MA, Group=BG, Sex=2, Age=2
ID=OGP_H_MA, Group=MA, Sex=2, Age=2
ID=OGT_H_MA, Group=MA, Sex=3, Age=1
ID=OGV_H_MA, Group=MA, Sex=2, Age=2
ID=OGV_V_BA, Group=BA, Sex=1, Age=2
ID=OM_H_MA, Group=MA, Sex=2, Age=1
ID=OMB_H_MA, Group=MA, Sex=1, Age=2
ID=OMB_V_BA, Group=BA, Sex=2, Age=3
Post Med Prob Choose Red
foraging bout
IL
FDSL
MaleBSL
AdultBSL
RoostBSL
RankBSL

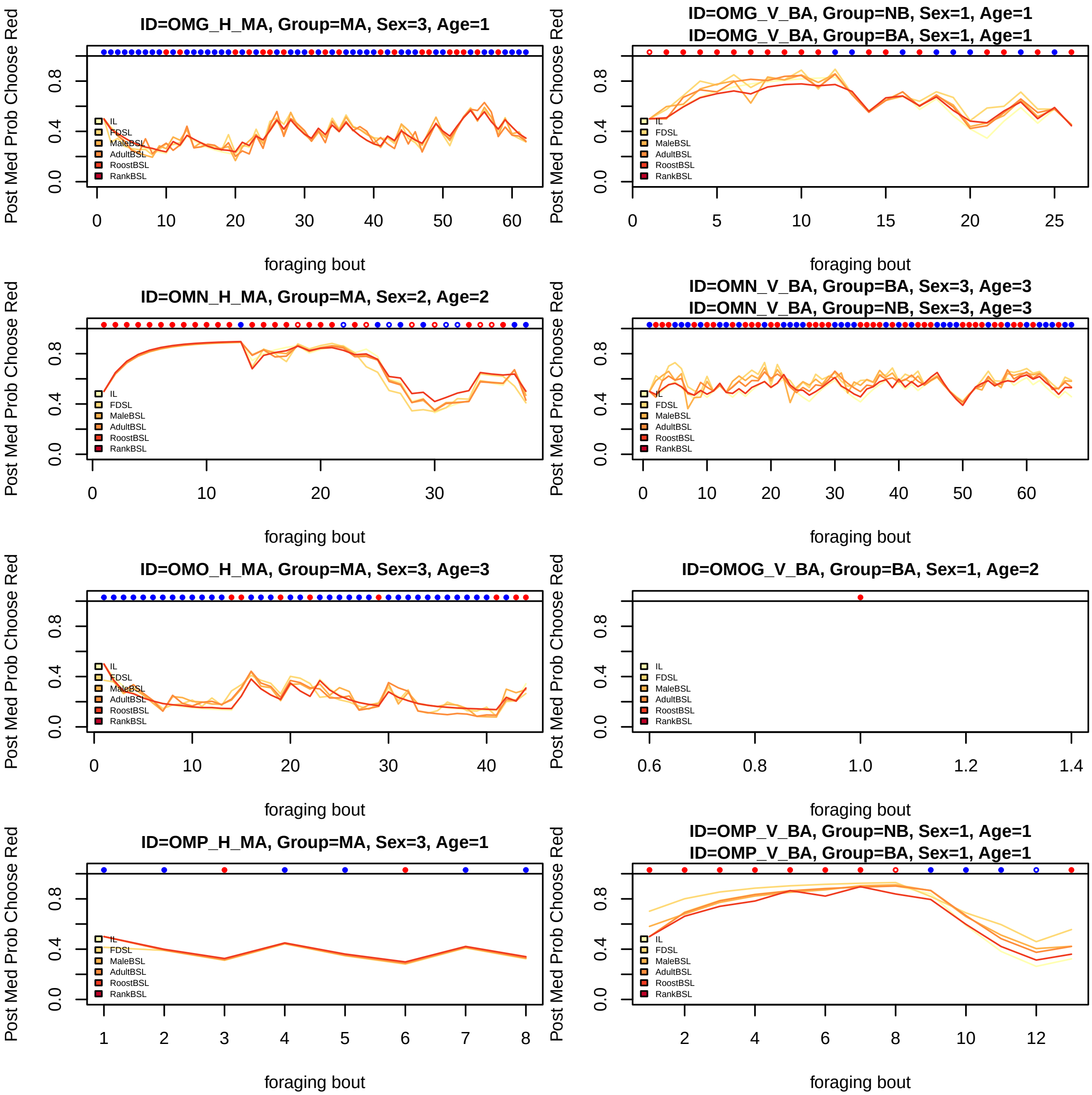
ID=OMG_H_MA, Group=MA, Sex=3, Age=1
ID=OMG_V_BA, Group=NB, Sex=1, Age=1
ID=OMG_V_BA, Group=BA, Sex=1, Age=1
ID=OMN_H_MA, Group=MA, Sex=2, Age=2
ID=OMN_V_BA, Group=BA, Sex=3, Age=3
ID=OMN_V_BA, Group=NB, Sex=3, Age=3
ID=OMO_H_MA, Group=MA, Sex=3, Age=3
ID=OMOG_V_BA, Group=BA, Sex=1, Age=2
ID=OMP_H_MA, Group=MA, Sex=3, Age=1
ID=OMP_V_BA, Group=NB, Sex=1, Age=1
ID=OMP_V_BA, Group=BA, Sex=1, Age=1
Post Med Prob Choose Red
foraging bout
IL
FDSL
MaleBSL
AdultBSL
RoostBSL
RankBSL

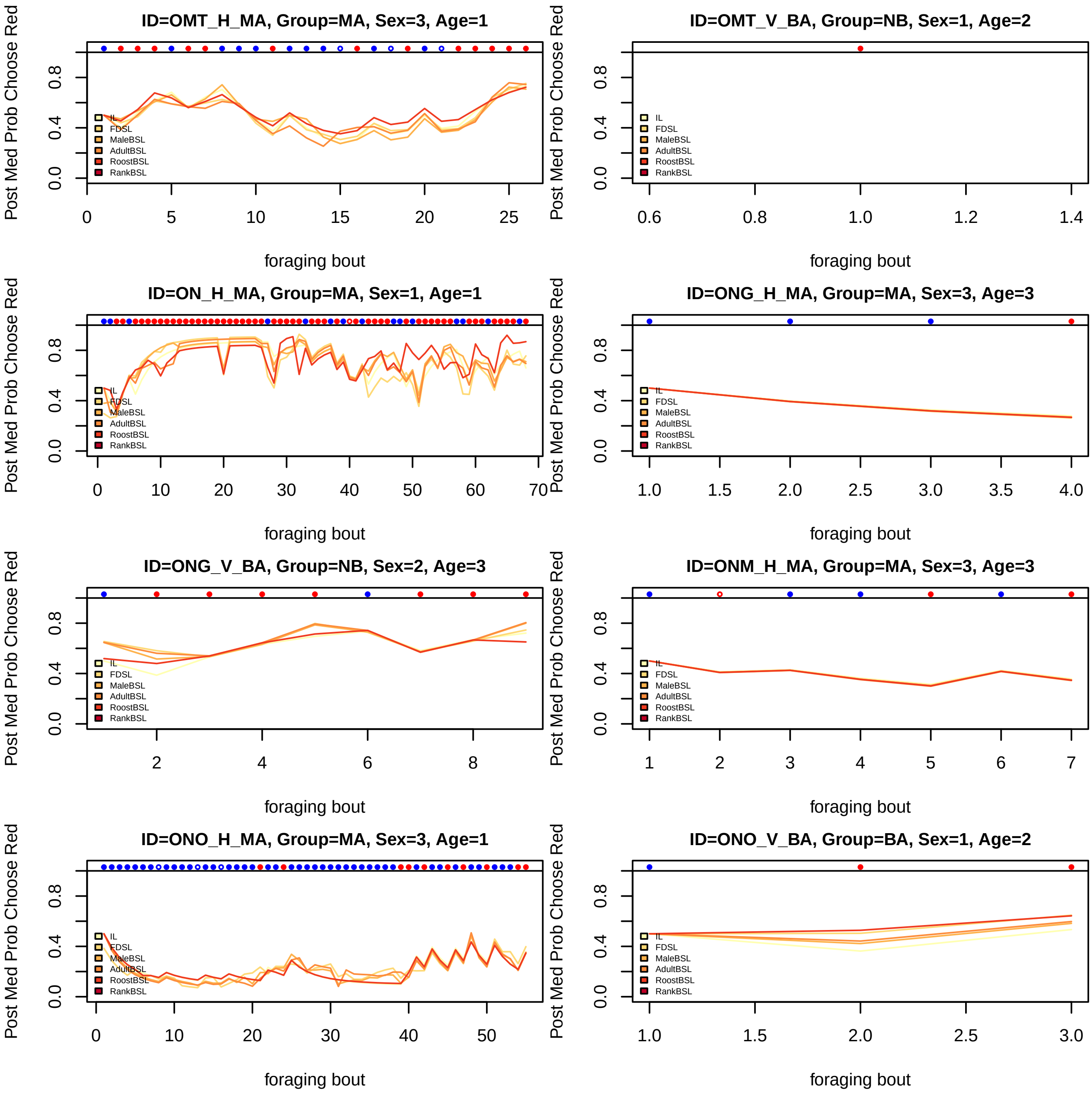
ID=OMT_H_MA, Group=MA, Sex=3, Age=1
ID=OMT_V_BA, Group=NB, Sex=1, Age=2
ID=ON_H_MA, Group=MA, Sex=1, Age=1
ID=ONG_H_MA, Group=MA, Sex=3, Age=3
ID=ONG_V_BA, Group=NB, Sex=2, Age=3
ID=ONM_H_MA, Group=MA, Sex=3, Age=3
ID=ONO_H_MA, Group=MA, Sex=3, Age=1
ID=ONO_V_BA, Group=BA, Sex=1, Age=2
Post Med Prob Choose Red
foraging bout
IL
FDSL
MaleBSL
AdultBSL
RoostBSL
RankBSL

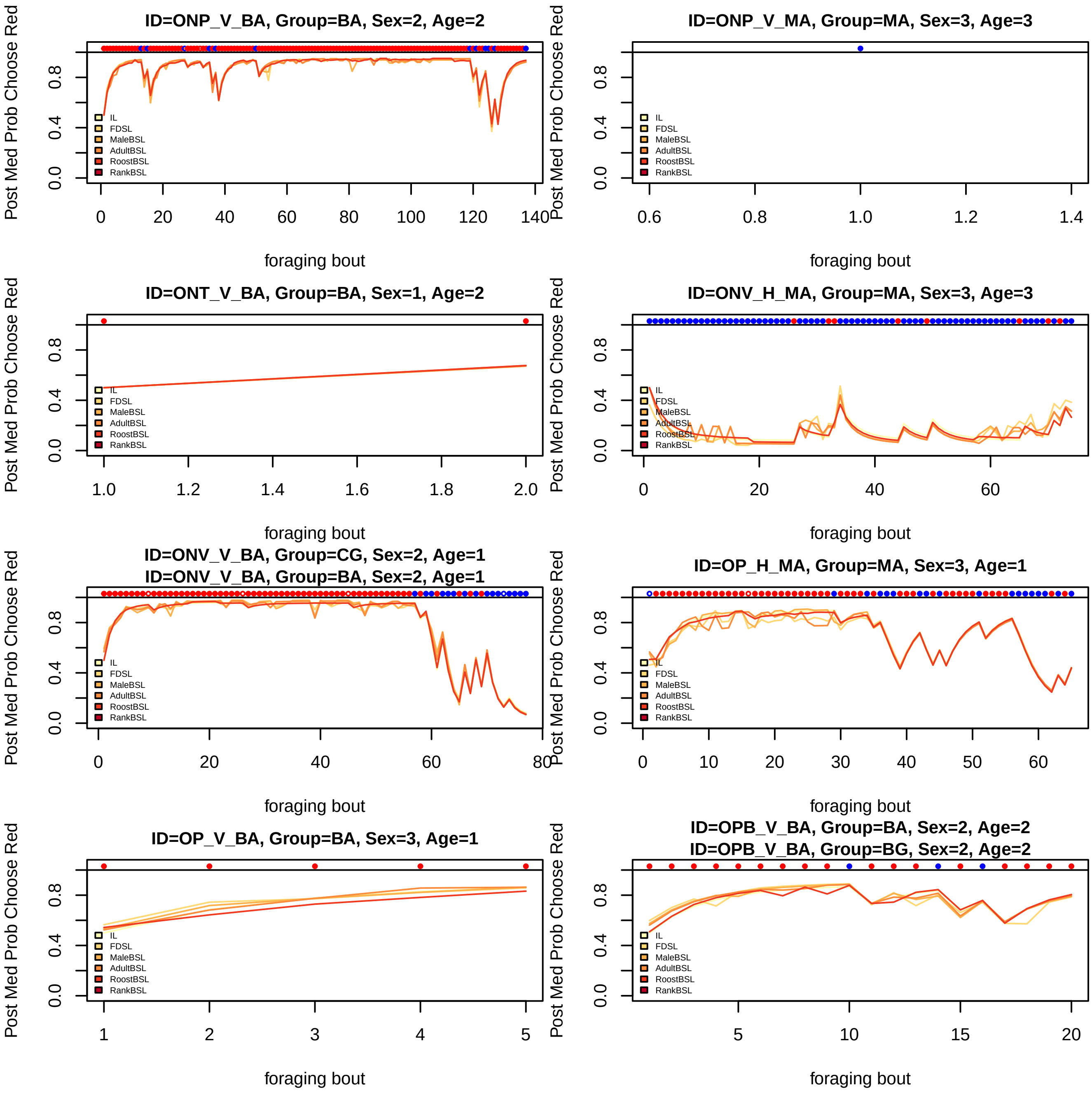
ID=ONP_V_BA, Group=BA, Sex=2, Age=2
ID=ONP_V_MA, Group=MA, Sex=3, Age=3
ID=ONT_V_BA, Group=BA, Sex=1, Age=2
ID=ONV_H_MA, Group=MA, Sex=3, Age=3
ID=ONV_V_BA, Group=CG, Sex=2, Age=1
ID=ONV_V_BA, Group=BA, Sex=2, Age=1
ID=OP_H_MA, Group=MA, Sex=3, Age=1
ID=OP_V_BA, Group=BA, Sex=3, Age=1
ID=OPB_V_BA, Group=BA, Sex=2, Age=2
ID=OPB_V_BA, Group=BG, Sex=2, Age=2
Post Med Prob Choose Red
foraging bout
IL
FDSL
MaleBSL
AdultBSL
RoostBSL
RankBSL

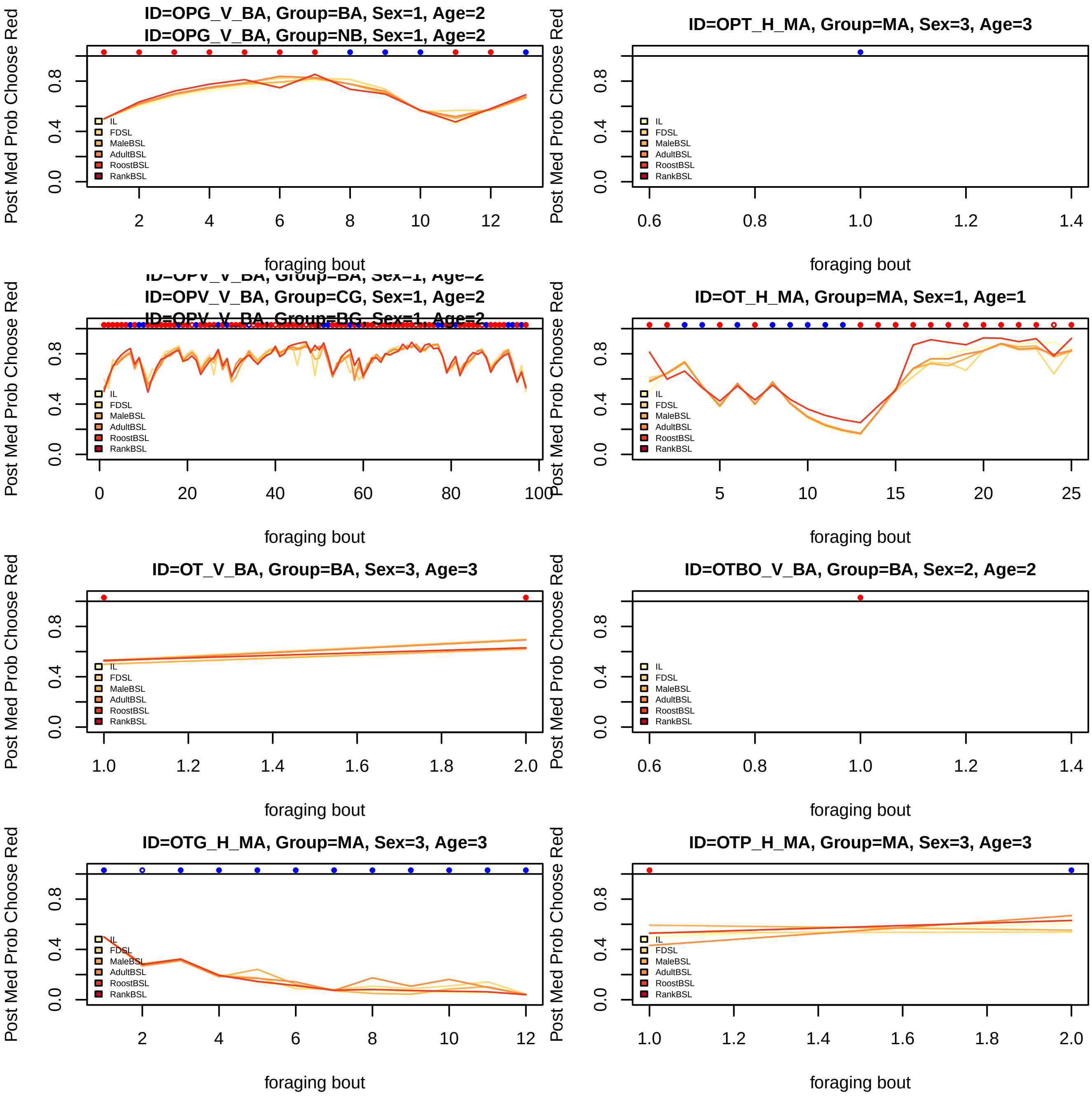
ID=OPG_V_BA, Group=BA, Sex=1, Age=2
ID=OPG_V_BA, Group=NB, Sex=1, Age=2
ID=OPT_H_MA, Group=MA, Sex=3, Age=3
ID=OPV_V_BA, Group=CG, Sex=1, Age=2
ID=OPV_V_BA, Group=BG, Sex=1, Age=2
ID=OT_H_MA, Group=MA, Sex=1, Age=1
ID=OT_V_BA, Group=BA, Sex=3, Age=3
ID=OTBO_V_BA, Group=BA, Sex=2, Age=2
ID=OTG_H_MA, Group=MA, Sex=3, Age=3
ID=OTP_H_MA, Group=MA, Sex=3, Age=3
Post Med Prob Choose Red
foraging bout
IL
FDSL
MaleBSL
AdultBSL
RoostBSL
RankBSL

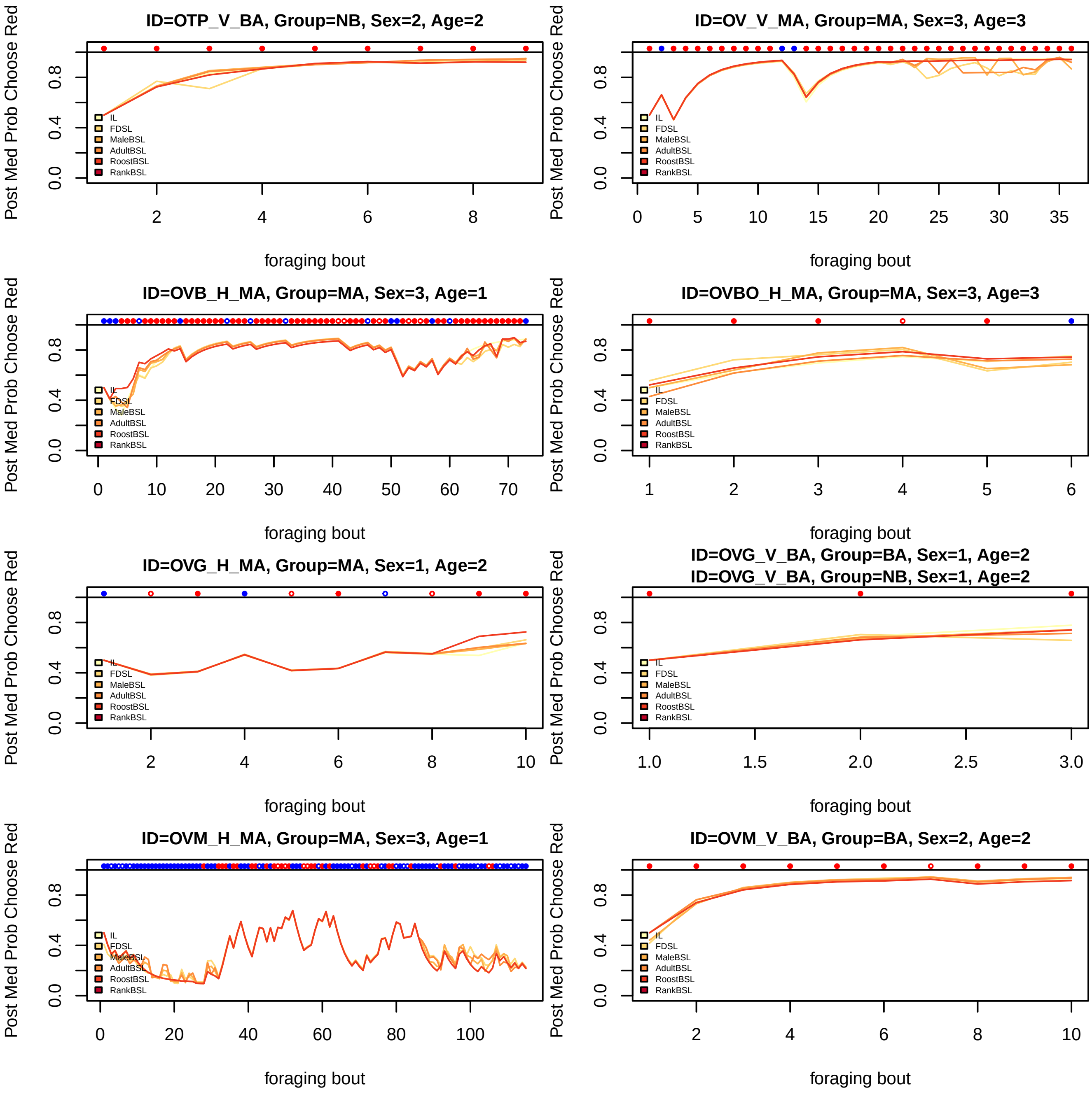
ID=OTP_V_BA, Group=NB, Sex=2, Age=2
ID=OV_V_MA, Group=MA, Sex=3, Age=3
ID=OVB_H_MA, Group=MA, Sex=3, Age=1
ID=OVBO_H_MA, Group=MA, Sex=3, Age=3
ID=OVG_H_MA, Group=MA, Sex=1, Age=2
ID=OVG_V_BA, Group=BA, Sex=1, Age=2
ID=OVG_V_BA, Group=NB, Sex=1, Age=2
ID=OVM_H_MA, Group=MA, Sex=3, Age=1
ID=OVM_V_BA, Group=BA, Sex=2, Age=2
Post Med Prob Choose Red
foraging bout
IL
FDSL
MaleBSL
AdultBSL
RoostBSL
RankBSL

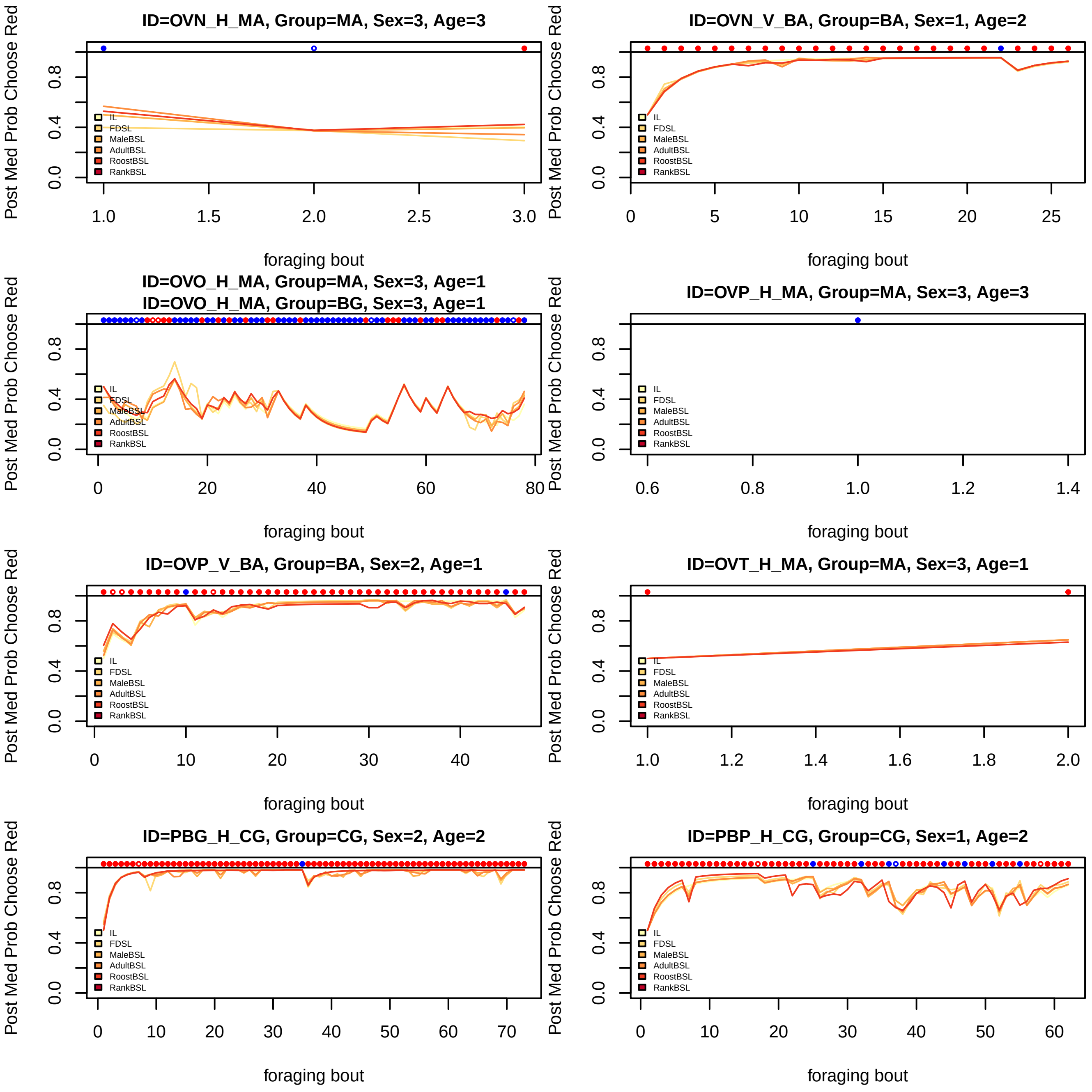
ID=OVN_H_MA, Group=MA, Sex=3, Age=3
ID=OVN_V_BA, Group=BA, Sex=1, Age=2
ID=OVO_H_MA, Group=MA, Sex=3, Age=1
ID=OVO_H_MA, Group=BG, Sex=3, Age=1
ID=OVP_H_MA, Group=MA, Sex=3, Age=3
ID=OVP_V_BA, Group=BA, Sex=2, Age=1
ID=OVT_H_MA, Group=MA, Sex=3, Age=1
ID=PBG_H_CG, Group=CG, Sex=2, Age=2
ID=PBP_H_CG, Group=CG, Sex=1, Age=2
Post Med Prob Choose Red
foraging bout
IL
FDSL
MaleBSL
AdultBSL
RoostBSL
RankBSL

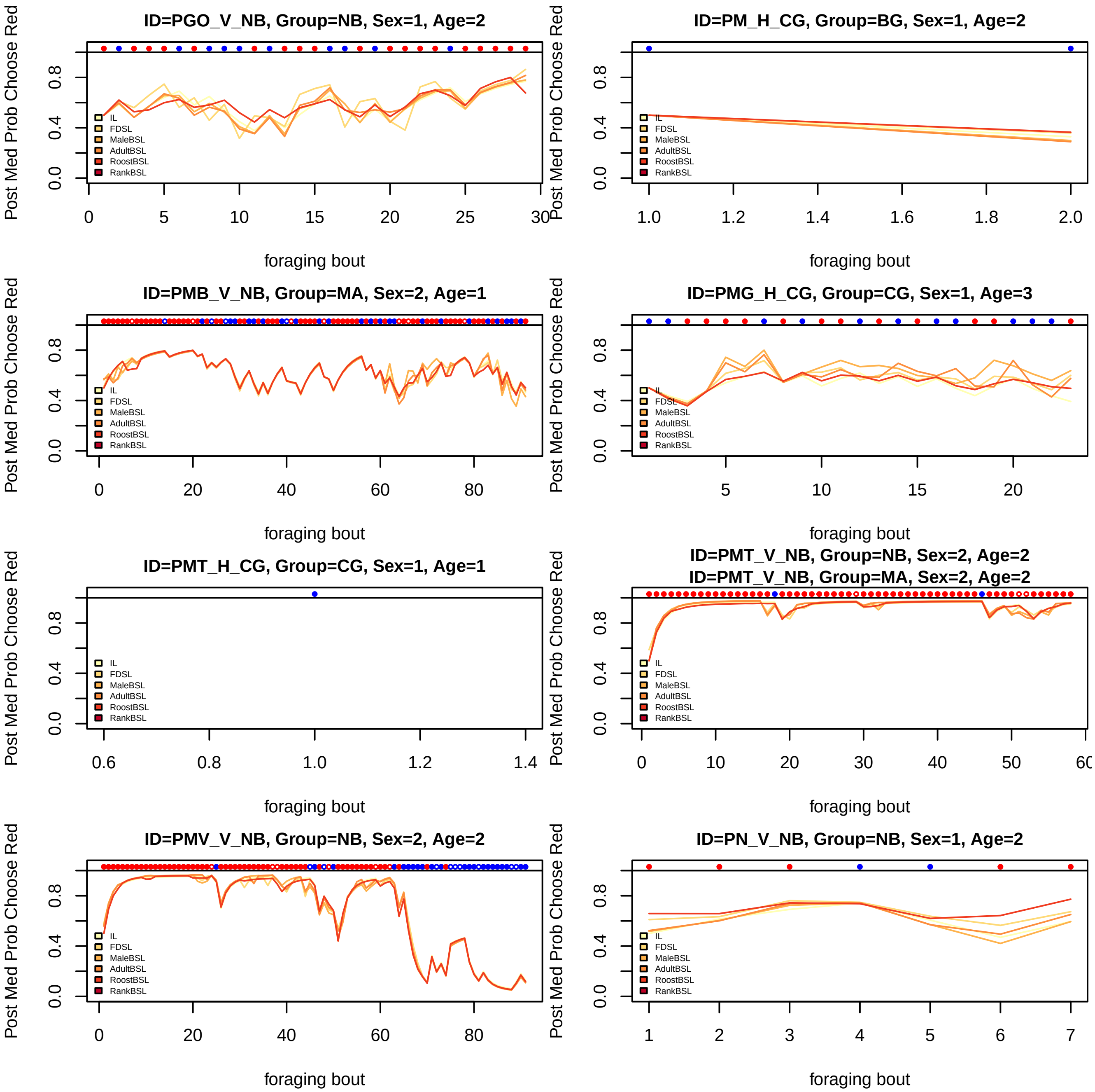
ID=PGO_V_NB, Group=NB, Sex=1, Age=2
ID=PM_H_CG, Group=BG, Sex=1, Age=2
ID=PMB_V_NB, Group=MA, Sex=2, Age=1
ID=PMG_H_CG, Group=CG, Sex=1, Age=3
ID=PMT_H_CG, Group=CG, Sex=1, Age=1
ID=PMT_V_NB, Group=NB, Sex=2, Age=2
ID=PMT_V_NB, Group=MA, Sex=2, Age=2
ID=PMV_V_NB, Group=NB, Sex=2, Age=2
ID=PN_V_NB, Group=NB, Sex=1, Age=2
Post Med Prob Choose Red
foraging bout
IL
FDSL
MaleBSL
AdultBSL
RoostBSL
RankBSL

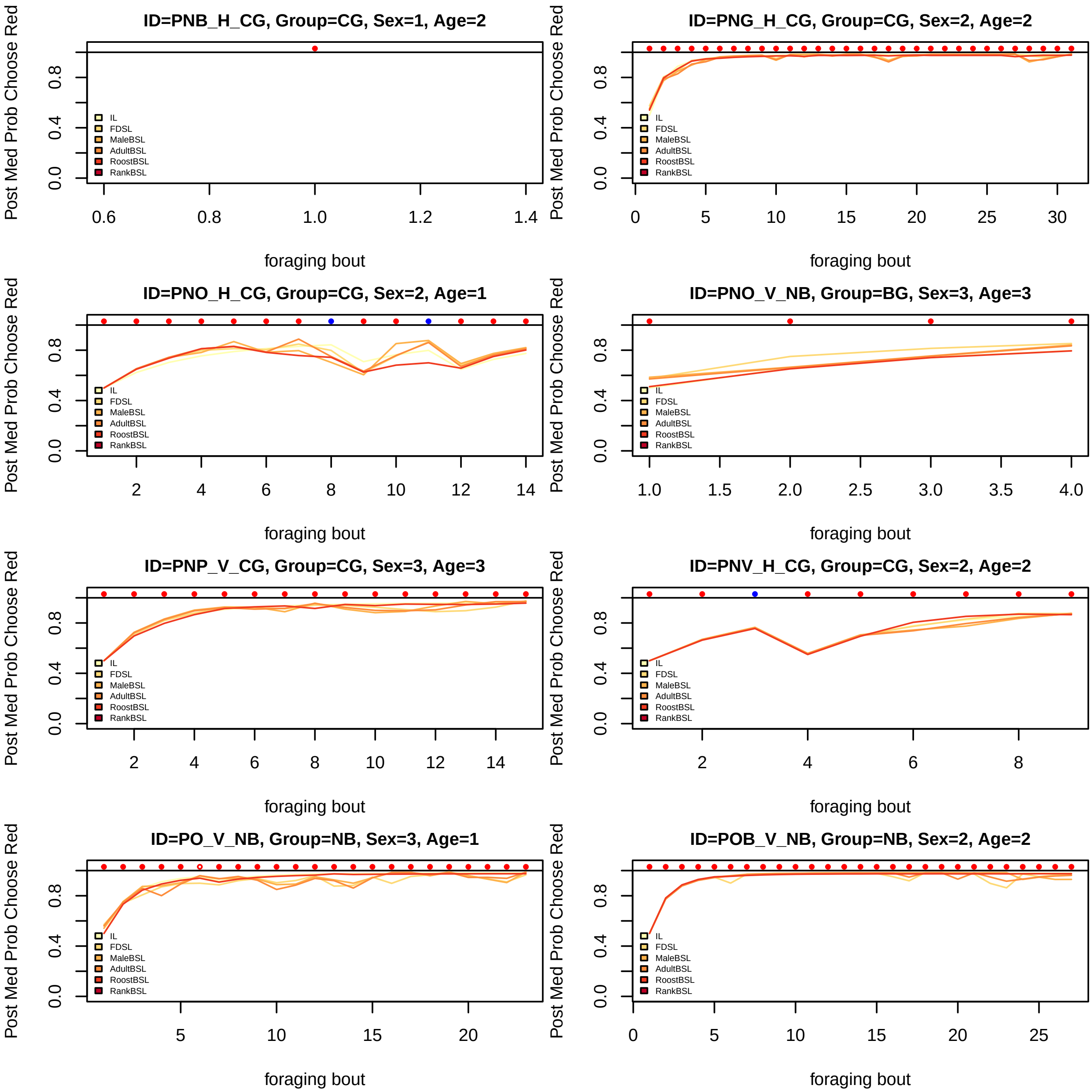
ID=PNB_H_CG, Group=CG, Sex=1, Age=2
ID=PNG_H_CG, Group=CG, Sex=2, Age=2
ID=PNO_H_CG, Group=CG, Sex=2, Age=1
ID=PNO_V_NB, Group=BG, Sex=3, Age=3
ID=PNP_V_CG, Group=CG, Sex=3, Age=3
ID=PNV_H_CG, Group=CG, Sex=2, Age=2
ID=PO_V_NB, Group=NB, Sex=3, Age=1
ID=POB_V_NB, Group=NB, Sex=2, Age=2
Post Med Prob Choose Red
foraging bout
IL
FDSL
MaleBSL
AdultBSL
RoostBSL
RankBSL

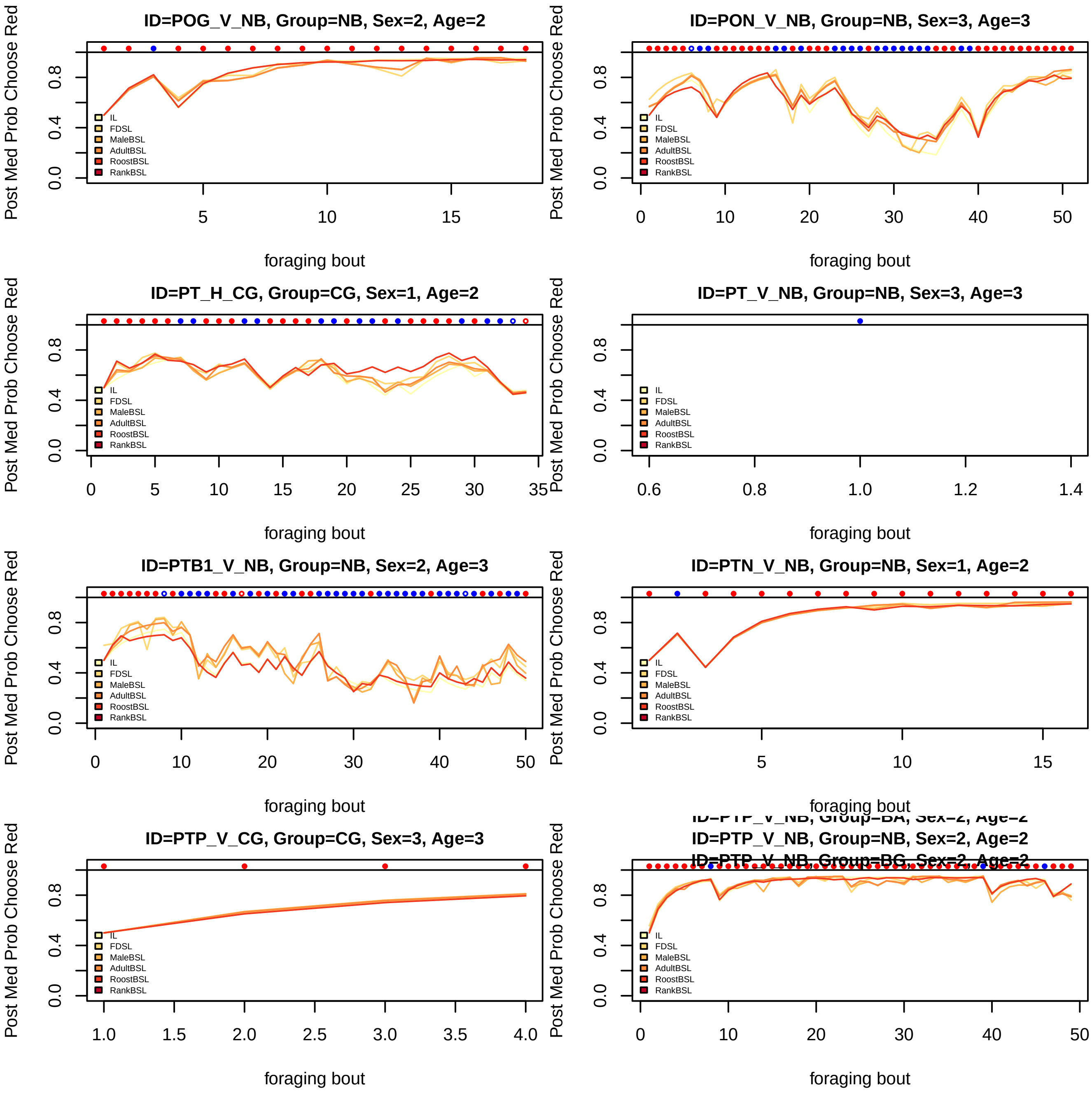
ID=POG_V_NB, Group=NB, Sex=2, Age=2
ID=PON_V_NB, Group=NB, Sex=3, Age=3
ID=PT_H_CG, Group=CG, Sex=1, Age=2
ID=PT_V_NB, Group=NB, Sex=3, Age=3
ID=PTB1_V_NB, Group=NB, Sex=2, Age=3
ID=PTN_V_NB, Group=NB, Sex=1, Age=2
ID=PTP_V_CG, Group=CG, Sex=3, Age=3
ID=PTP_V_NB, Group=NB, Sex=2, Age=2
ID=PTP_V_NB, Group=BG, Sex=2, Age=2
Post Med Prob Choose Red
foraging bout
IL
FDSL
MaleBSL
AdultBSL
RoostBSL
RankBSL

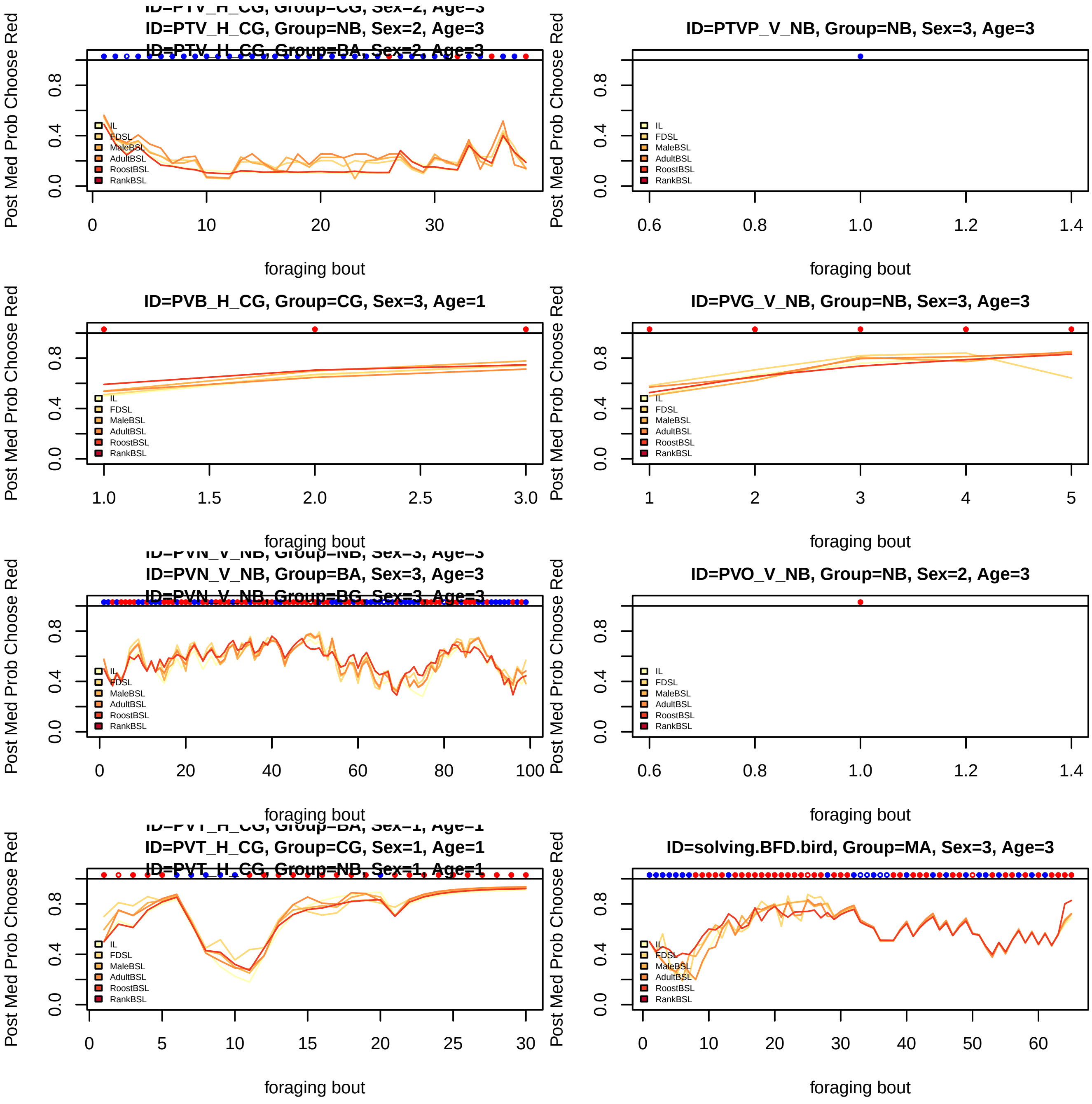
ID=PTV_H_CG, Group=NB, Sex=2, Age=3
ID=PTVP_V_NB, Group=NB, Sex=3, Age=3
ID=PVB_H_CG, Group=CG, Sex=3, Age=1
ID=PVG_V_NB, Group=NB, Sex=3, Age=3
ID=PVN_V_NB, Group=BA, Sex=3, Age=3
ID=PVO_V_NB, Group=NB, Sex=2, Age=3
ID=PVT_H_CG, Group=CG, Sex=1, Age=1
ID=solving.BFD.bird, Group=MA, Sex=3, Age=3
Post Med Prob Choose Red
foraging bout
IL
FDSL
MaleBSL
AdultBSL
RoostBSL
RankBSL

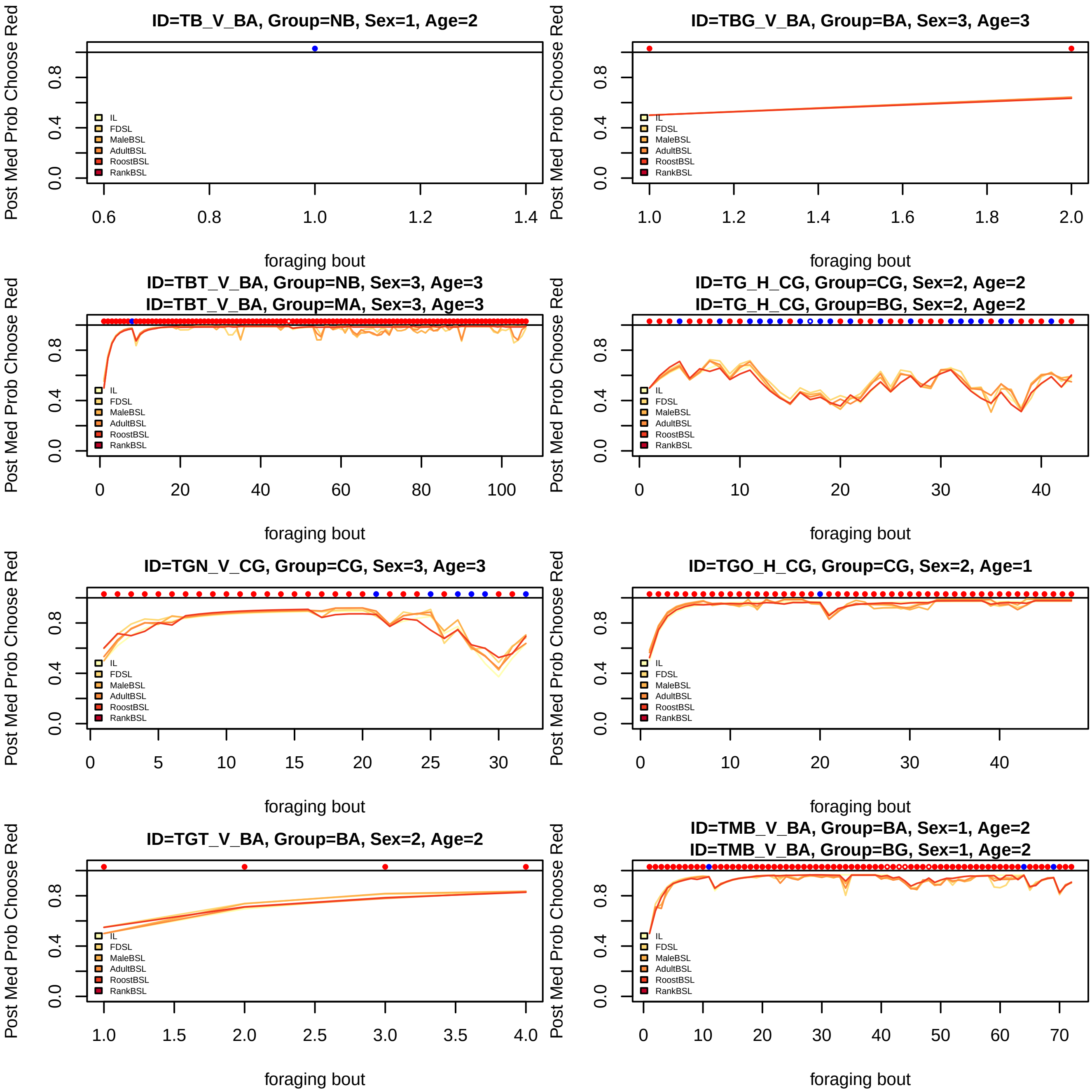
ID=TB_V_BA, Group=NB, Sex=1, Age=2
ID=TBG_V_BA, Group=BA, Sex=3, Age=3
ID=TBT_V_BA, Group=NB, Sex=3, Age=3
ID=TBT_V_BA, Group=MA, Sex=3, Age=3
ID=TG_H_CG, Group=CG, Sex=2, Age=2
ID=TG_H_CG, Group=BG, Sex=2, Age=2
ID=TGN_V_CG, Group=CG, Sex=3, Age=3
ID=TGO_H_CG, Group=CG, Sex=2, Age=1
ID=TGT_V_BA, Group=BA, Sex=2, Age=2
ID=TMB_V_BA, Group=BA, Sex=1, Age=2
ID=TMB_V_BA, Group=BG, Sex=1, Age=2
Post Med Prob Choose Red
foraging bout
IL
FDSL
MaleBSL
AdultBSL
RoostBSL
RankBSL

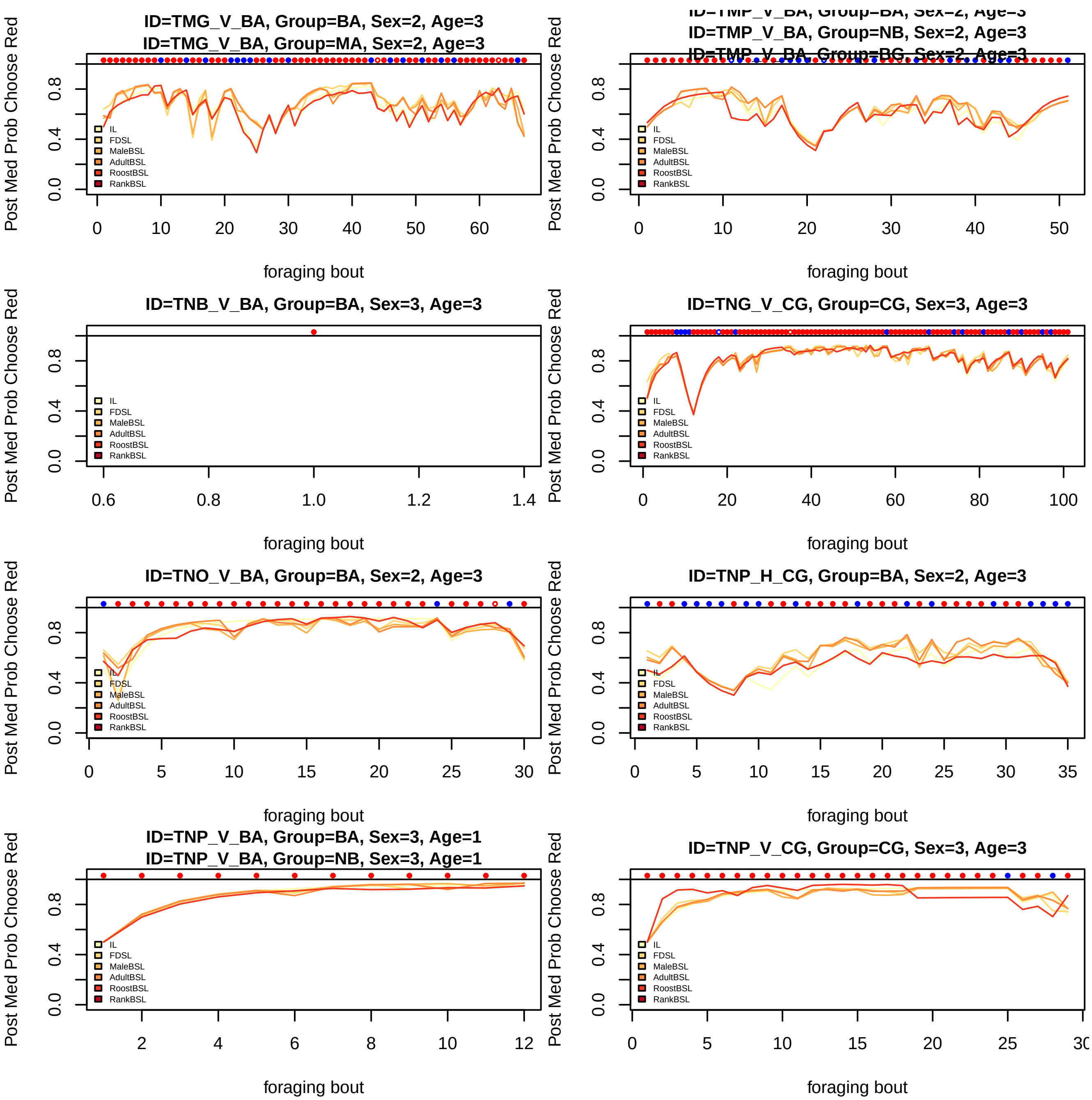
ID=TMG_V_BA, Group=BA, Sex=2, Age=3
ID=TMG_V_BA, Group=MA, Sex=2, Age=3
ID=TMP_V_BA, Group=NB, Sex=2, Age=3
ID=TMP_V_BA, Group=BG, Sex=2, Age=3
ID=TNB_V_BA, Group=BA, Sex=3, Age=3
ID=TNG_V_CG, Group=CG, Sex=3, Age=3
ID=TNO_V_BA, Group=BA, Sex=2, Age=3
ID=TNP_H_CG, Group=BA, Sex=2, Age=3
ID=TNP_V_BA, Group=BA, Sex=3, Age=1
ID=TNP_V_BA, Group=NB, Sex=3, Age=1
ID=TNP_V_CG, Group=CG, Sex=3, Age=3
Post Med Prob Choose Red
foraging bout
IL
FDSL
MaleBSL
AdultBSL
RoostBSL
RankBSL

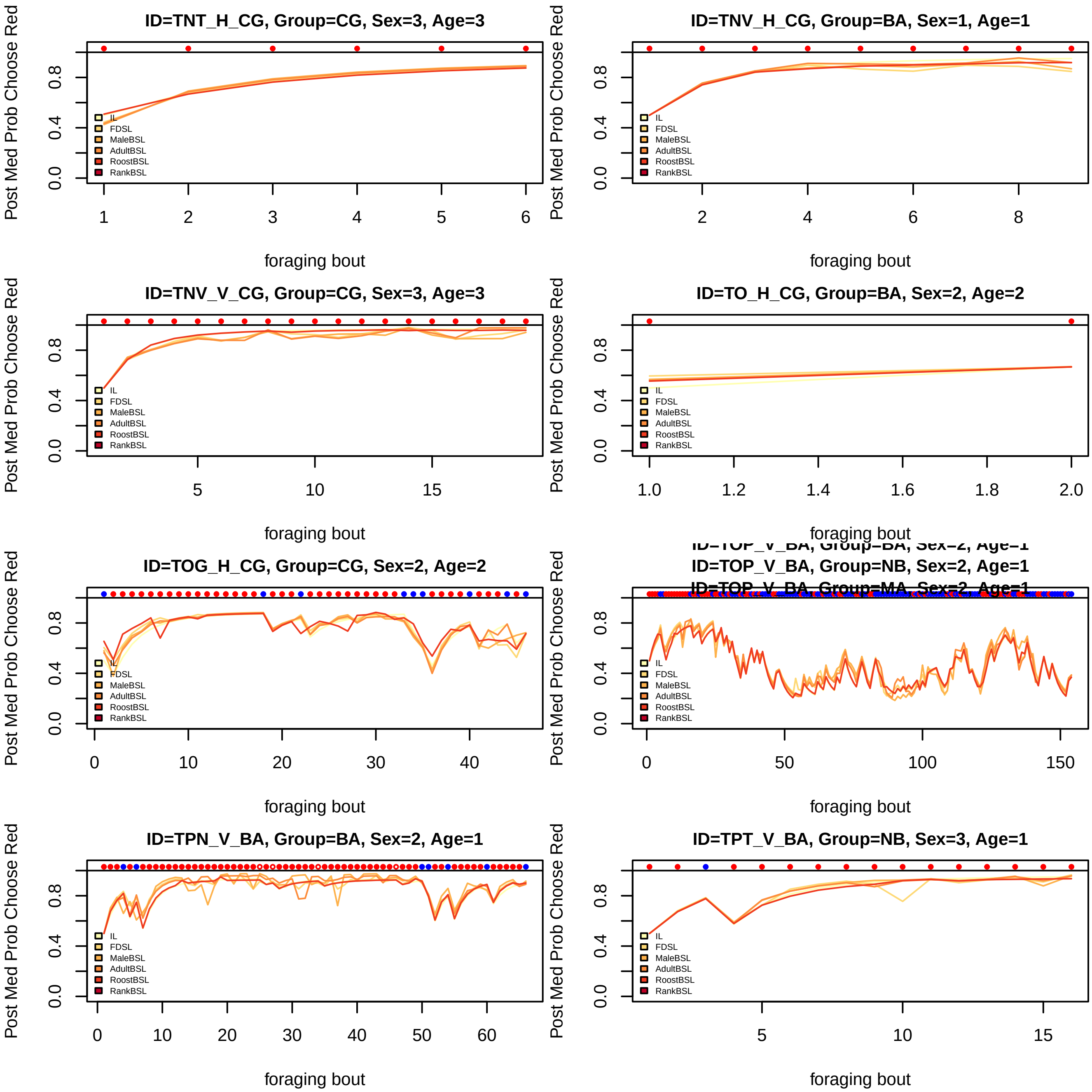
ID=TNT_H_CG, Group=CG, Sex=3, Age=3
ID=TNV_H_CG, Group=BA, Sex=1, Age=1
ID=TNV_V_CG, Group=CG, Sex=3, Age=3
ID=TO_H_CG, Group=BA, Sex=2, Age=2
ID=TOG_H_CG, Group=CG, Sex=2, Age=2
ID=TOP_V_BA, Group=NB, Sex=2, Age=1
ID=TPN_V_BA, Group=BA, Sex=2, Age=1
ID=TPT_V_BA, Group=NB, Sex=3, Age=1
Post Med Prob Choose Red
foraging bout
IL
FDSL
MaleBSL
AdultBSL
RoostBSL
RankBSL

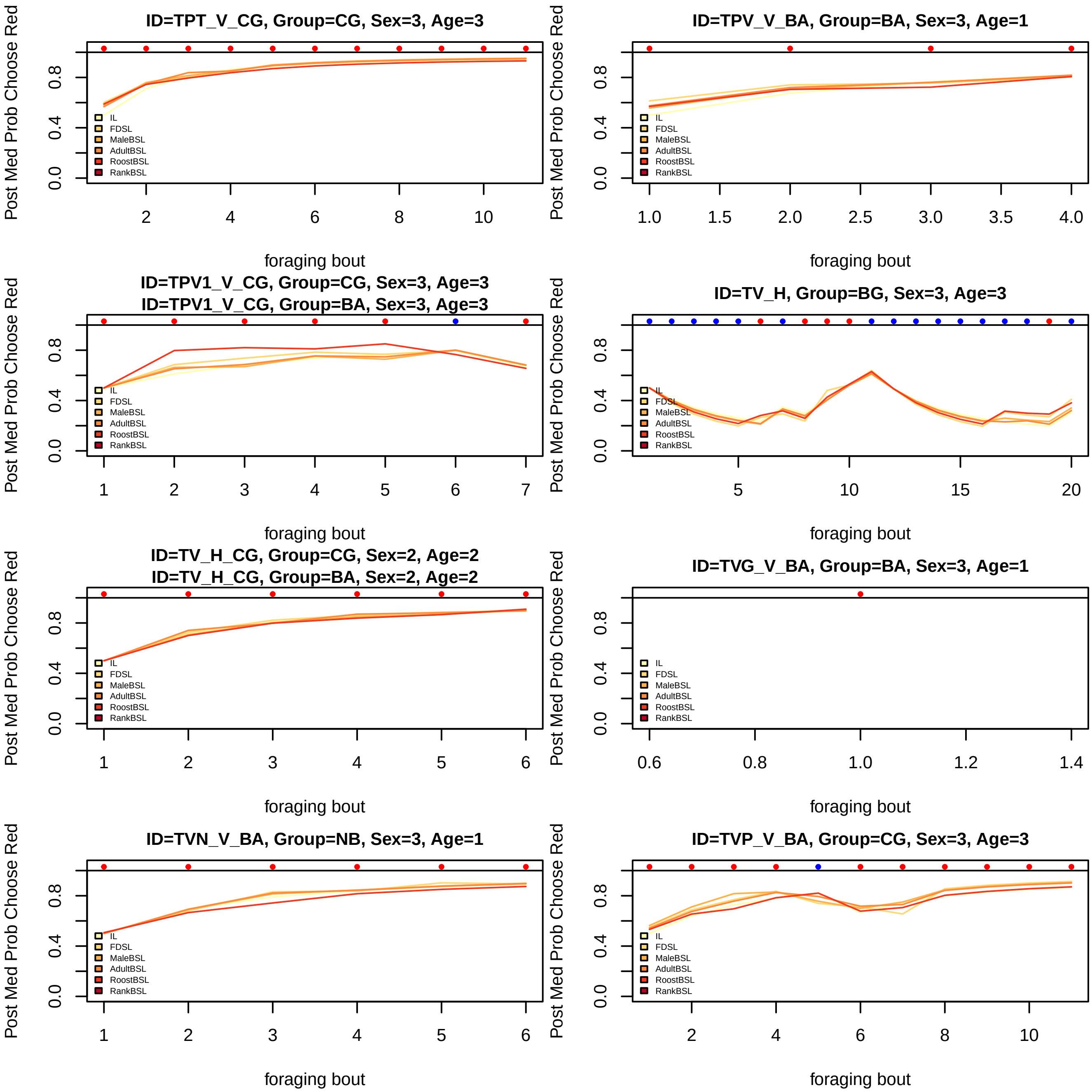

ID=TPT_V_CG, Group=CG, Sex=3, Age=3
ID=TPV_V_BA, Group=BA, Sex=3, Age=1
ID=TPV1_V_CG, Group=CG, Sex=3, Age=3
ID=TPV1_V_CG, Group=BA, Sex=3, Age=3
ID=TV_H, Group=BG, Sex=3, Age=3
ID=TV_H_CG, Group=CG, Sex=2, Age=2
ID=TV_H_CG, Group=BA, Sex=2, Age=2
ID=TVG_V_BA, Group=BA, Sex=3, Age=1
ID=TVN_V_BA, Group=NB, Sex=3, Age=1
ID=TVP_V_BA, Group=CG, Sex=3, Age=3
Post Med Prob Choose Red
foraging bout
IL
FDSL
MaleBSL
AdultBSL
RoostBSL
RankBSL

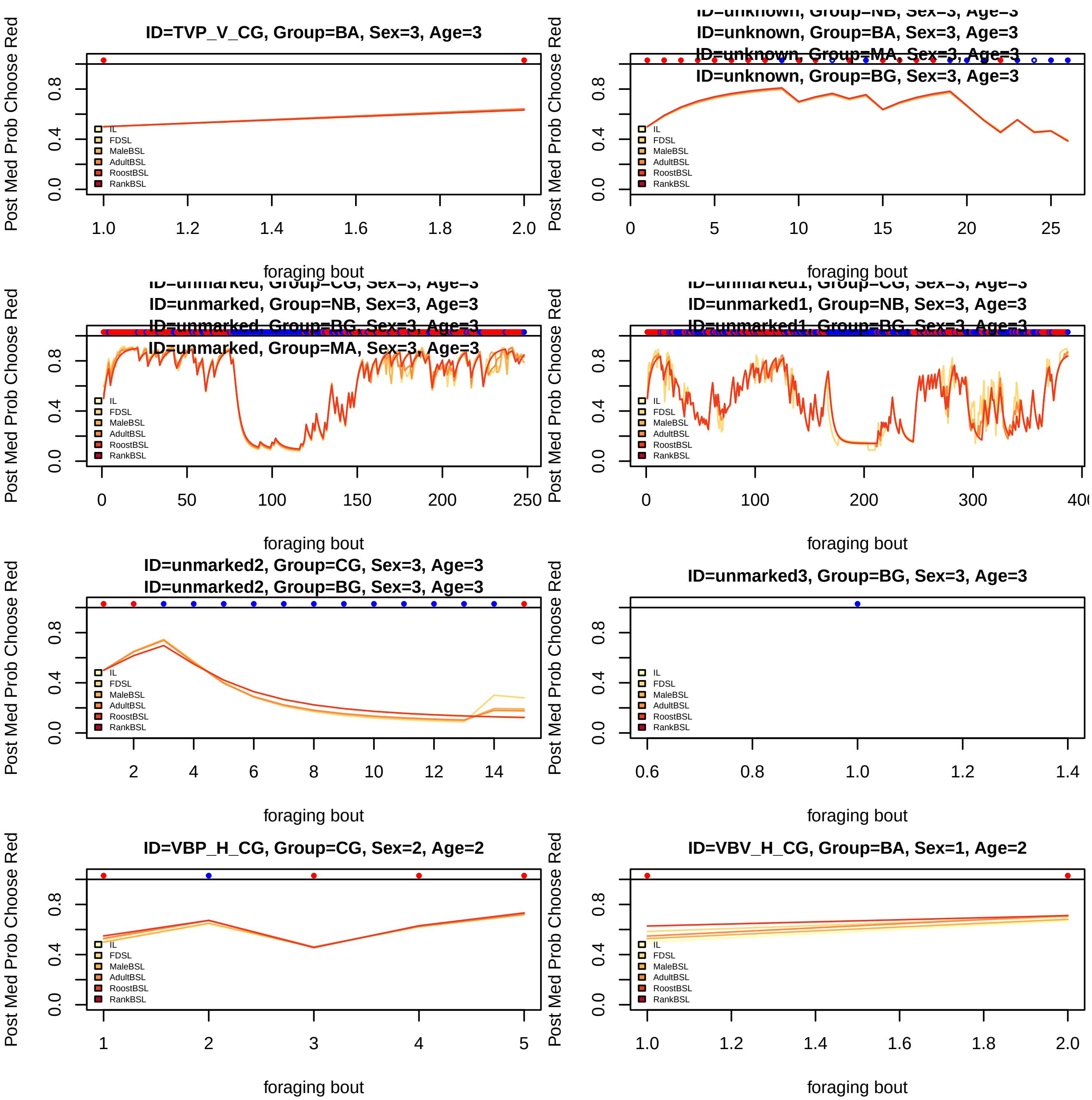
ID=TVP_V_CG, Group=BA, Sex=3, Age=3
ID=unknown, Group=BA, Sex=3, Age=3
ID=unknown, Group=MA, Sex=3, Age=3
ID=unknown, Group=BG, Sex=3, Age=3
ID=unmarked, Group=NB, Sex=3, Age=3
ID=unmarked, Group=BG, Sex=3, Age=3
ID=unmarked, Group=MA, Sex=3, Age=3
ID=unmarked1, Group=NB, Sex=3, Age=3
ID=unmarked1, Group=BG, Sex=3, Age=3
ID=unmarked2, Group=CG, Sex=3, Age=3
ID=unmarked2, Group=BG, Sex=3, Age=3
ID=unmarked3, Group=BG, Sex=3, Age=3
ID=VBP_H_CG, Group=CG, Sex=2, Age=2
ID=VBV_H_CG, Group=BA, Sex=1, Age=2
Post Med Prob Choose Red
foraging bout
IL
FDSL
MaleBSL
AdultBSL
RoostBSL
RankBSL

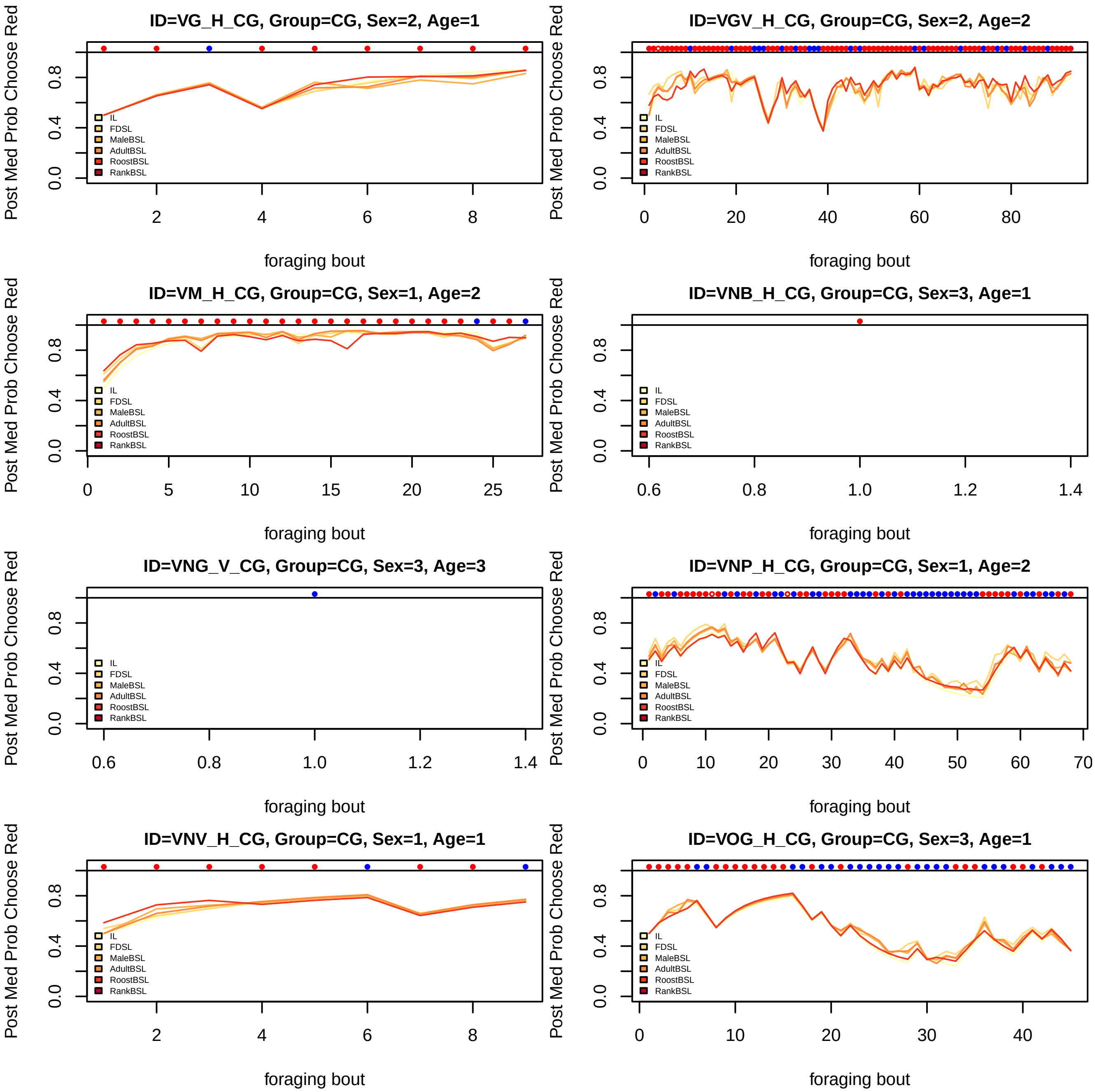
ID=VG_H_CG, Group=CG, Sex=2, Age=1
ID=VGV_H_CG, Group=CG, Sex=2, Age=2
ID=VM_H_CG, Group=CG, Sex=1, Age=2
ID=VNB_H_CG, Group=CG, Sex=3, Age=1
ID=VNG_V_CG, Group=CG, Sex=3, Age=3
ID=VNP_H_CG, Group=CG, Sex=1, Age=2
ID=VNV_H_CG, Group=CG, Sex=1, Age=1
ID=VOG_H_CG, Group=CG, Sex=3, Age=1
Post Med Prob Choose Red
foraging bout
IL
FDSL
MaleBSL
AdultBSL
RoostBSL
RankBSL

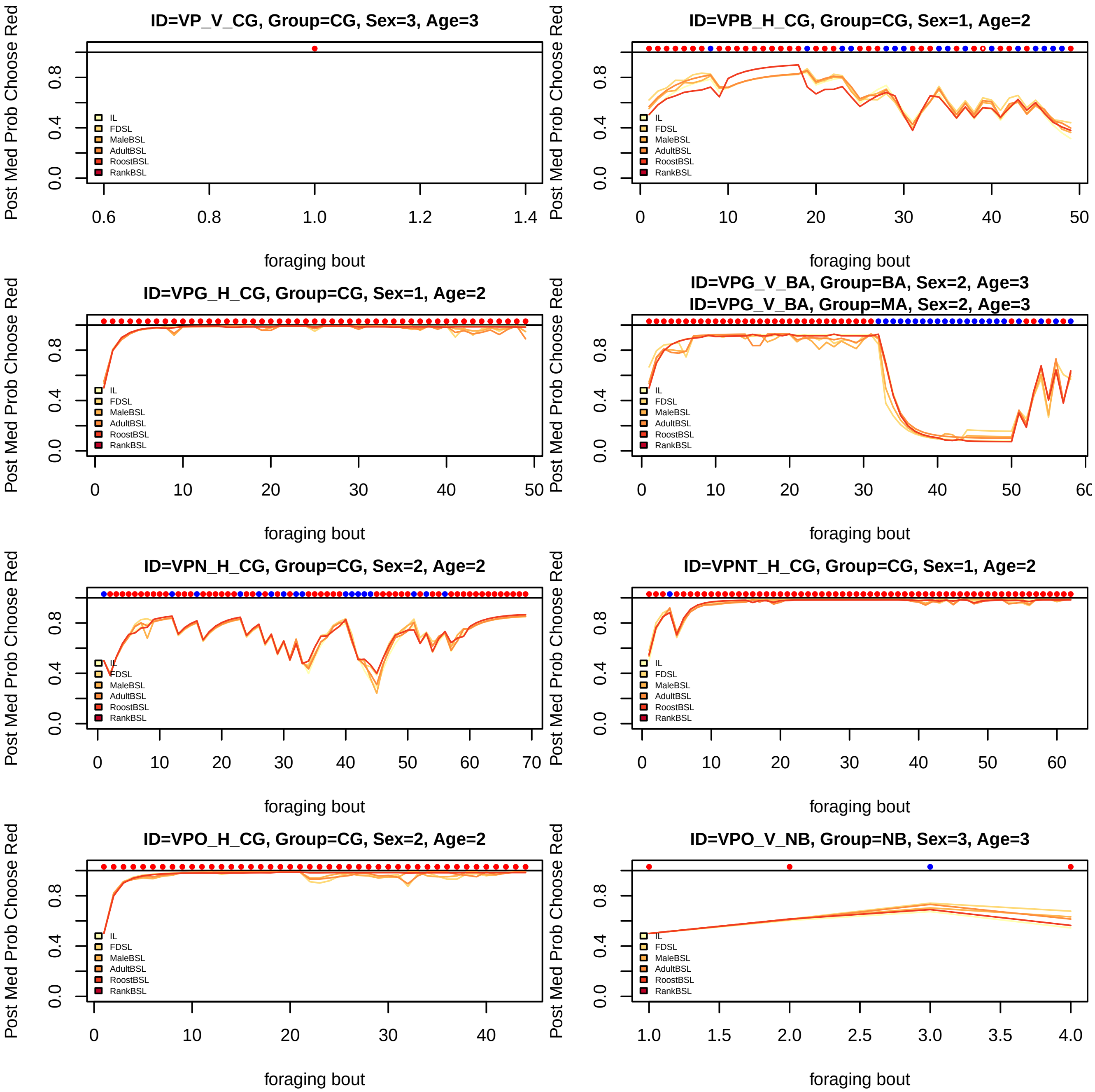
ID=VP_V_CG, Group=CG, Sex=3, Age=3
ID=VPB_H_CG, Group=CG, Sex=1, Age=2
ID=VPG_H_CG, Group=CG, Sex=1, Age=2
ID=VPG_V_BA, Group=BA, Sex=2, Age=3
ID=VPG_V_BA, Group=MA, Sex=2, Age=3
ID=VPN_H_CG, Group=CG, Sex=2, Age=2
ID=VPNT_H_CG, Group=CG, Sex=1, Age=2
ID=VPO_H_CG, Group=CG, Sex=2, Age=2
ID=VPO_V_NB, Group=NB, Sex=3, Age=3
Post Med Prob Choose Red
foraging bout
IL
FDSL
MaleBSL
AdultBSL
RoostBSL
RankBSL

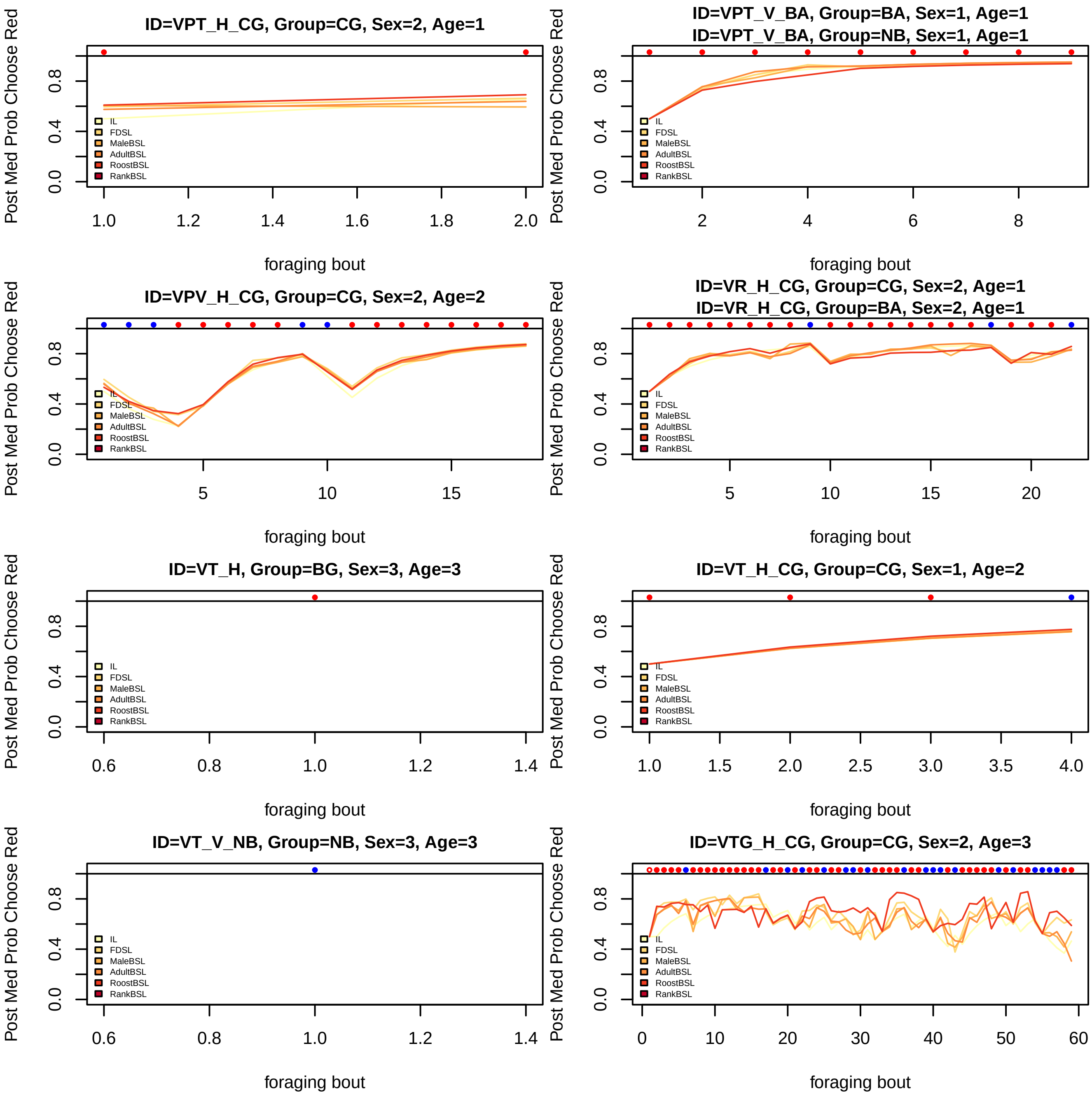
ID=VPT_H_CG, Group=CG, Sex=2, Age=1
ID=VPT_V_BA, Group=BA, Sex=1, Age=1
ID=VPT_V_BA, Group=NB, Sex=1, Age=1
ID=VPV_H_CG, Group=CG, Sex=2, Age=2
ID=VR_H_CG, Group=CG, Sex=2, Age=1
ID=VR_H_CG, Group=BA, Sex=2, Age=1
ID=VT_H, Group=BG, Sex=3, Age=3
ID=VT_H_CG, Group=CG, Sex=1, Age=2
ID=VT_V_NB, Group=NB, Sex=3, Age=3
ID=VTG_H_CG, Group=CG, Sex=2, Age=3
Post Med Prob Choose Red
foraging bout
IL
FDSL
MaleBSL
AdultBSL
RoostBSL
RankBSL

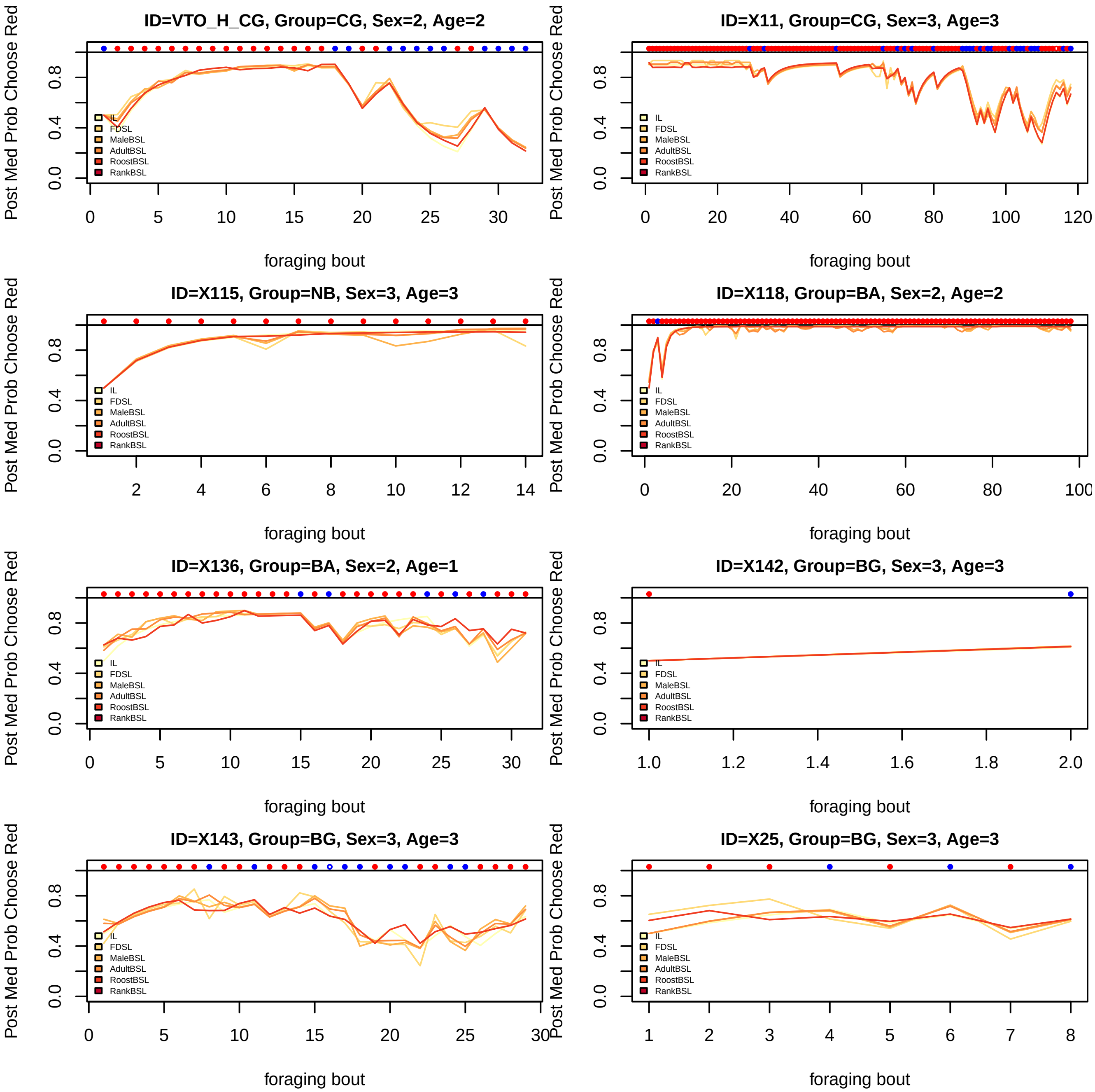

ID=VTO_H_CG, Group=CG, Sex=2, Age=2
ID=X11, Group=CG, Sex=3, Age=3
ID=X115, Group=NB, Sex=3, Age=3
ID=X118, Group=BA, Sex=2, Age=2
ID=X136, Group=BA, Sex=2, Age=1
ID=X142, Group=BG, Sex=3, Age=3
ID=X143, Group=BG, Sex=3, Age=3
ID=X25, Group=BG, Sex=3, Age=3
Post Med Prob Choose Red
foraging bout
0.0
0.4
0.8
IL
FDSL
MaleBSL
AdultBSL
RoostBSL
RankBSL

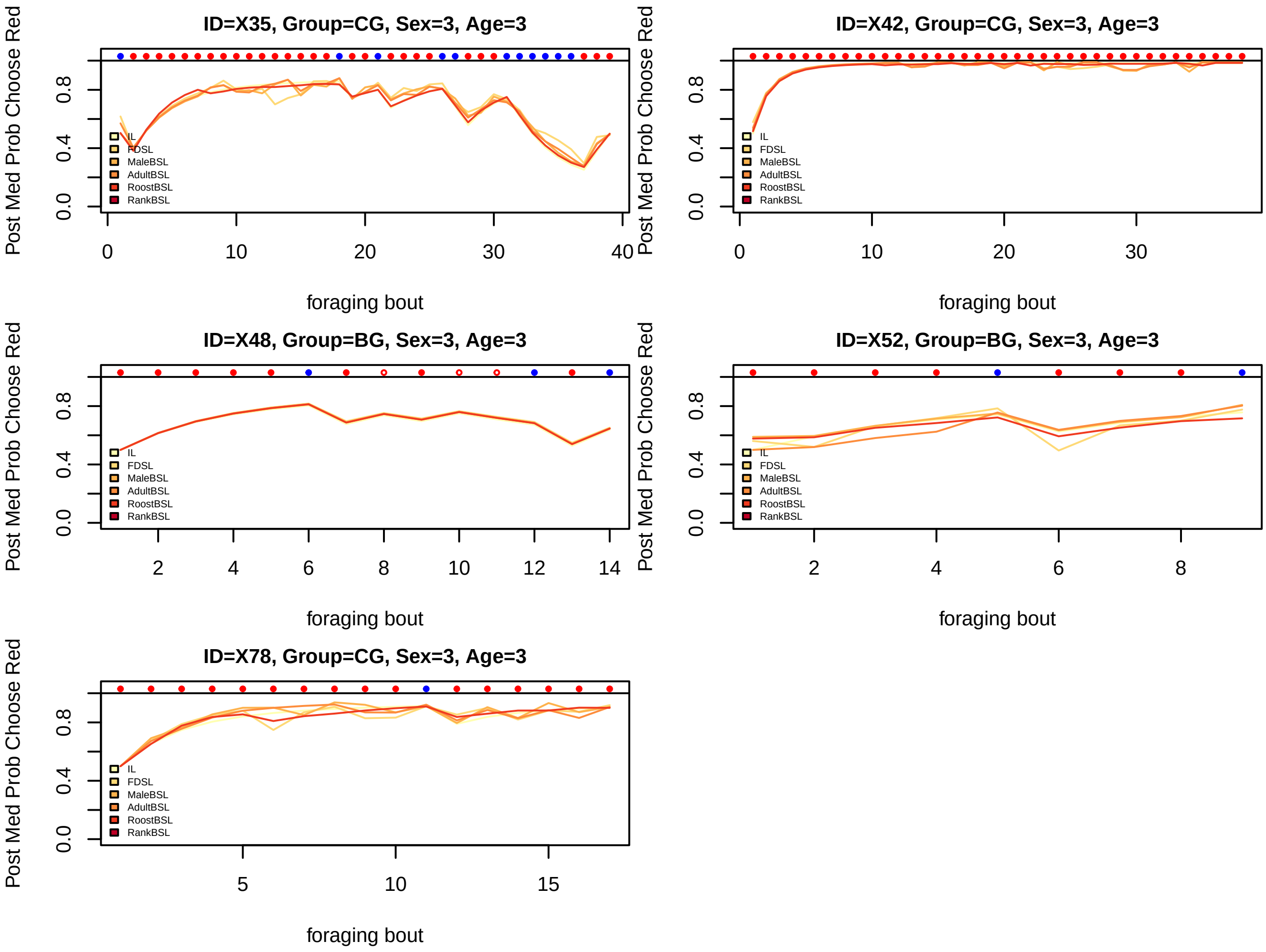
ID=X35, Group=CG, Sex=3, Age=3
ID=X42, Group=CG, Sex=3, Age=3
ID=X48, Group=BG, Sex=3, Age=3
ID=X52, Group=BG, Sex=3, Age=3
ID=X78, Group=CG, Sex=3, Age=3
Post Med Prob Choose Red
foraging bout
IL
FDSL
MaleBSL
AdultBSL
RoostBSL
RankBSL
0.0
0.4
0.8

Appendix 0—figure 8: Model predictions of the probability of choosing red across foraging bouts. Each panel represents one individual. The ID, sex and age of the individual are indicated at the top of each panel. The top row of each graph represents the colour choice of the individual (red or blue) at each foraging bouts. Filled dots represent success, while empty circles represent failures (i.e. individuals dropping the almond with 3s after picking it up from the dispenser). Coloured lines represent the prediction by each of the considered models. The roost(s) recorded at the top of each graph show at which site(s) an individual solved over the course of the experiment. The data underlying this figure can be found in our data and code repository (https://doi.org/10.5281/zenodo.19052060).
