# Supplementary material for: Wild parrots exhibit age-dependent conformity when learning about novel food
Source: PLoS Biol. 2026 Apr 30;24(4):e3003741. doi: 10.1371/journal.pbio.3003741 (PMC13132227; doi:10.1371/journal.pbio.3003741)
Supplement: S1 Table — (PDF) [file pbio.3003741.s003.pdf]

Description of the five best NBDA-models, and their respective Akaike weights.

Predictors	Akaike Weight
Social network, no ILVs	0.280
Social network, sex influencing asocial learning	0.111
Social network, age influencing asocial learning	0.108
Social network, age influencing social learning	0.102
Social network, sex influencing social learning	0.101
