# Supplementary material for: Wild parrots exhibit age-dependent conformity when learning about novel food
Source: PLoS Biol. 2026 Apr 30;24(4):e3003741. doi: 10.1371/journal.pbio.3003741 (PMC13132227; doi:10.1371/journal.pbio.3003741)
Supplement: S2 Table — (PDF) [file pbio.3003741.s004.pdf]

Parameter estimates for each age (a=adult, j: juvenile, u: unknown) and sex (f: female, m: male, u: unknown) group and each model.

Parameter	Ind. Learn	Freq. Dep	Male-bias	Adult-bias	Roost-Bias
lambda[a,f]	2.66	2.90	2.78	2.72	2.56
lambda[a,m]	2.35	2.53	2.61	2.48	2.54
lambda[a,u]	2.63	2.93	2.84	2.78	2.58
lambda[j,f]	2.80	2.83	2.81	2.81	3.05
lambda[j,m]	2.44	2.41	2.47	2.45	2.57
lambda[j,u]	2.70	2.72	2.71	2.69	2.70
lambda[u,f]	1.98	1.98	2.01	1.89	1.89
lambda[u,m]	1.76	1.47	1.62	1.63	1.76
lambda[u,u]	2.24	2.56	2.46	2.47	2.55
phi[a,f]	0.25	0.23	0.25	0.25	0.24
phi[a,m]	0.22	0.22	0.21	0.22	0.22
phi[a,u]	0.18	0.17	0.17	0.18	0.17
phi[j,f]	0.20	0.21	0.20	0.20	0.20
phi[j,m]	0.23	0.24	0.23	0.23	0.24
phi[j,u]	0.51	0.50	0.50	0.50	0.50
phi[u,f]	0.21	0.25	0.23	0.24	0.22
phi[u,m]	0.24	0.26	0.27	0.29	0.28
phi[u,u]	0.19	0.18	0.19	0.19	0.19
gamma[a,f]		0.35	0.15	0.13	0.14
gamma[a,m]		0.17	0.19	0.14	0.16
gamma[a,u]		0.22	0.10	0.14	0.07
gamma[j,f]		0.19	0.08	0.11	0.13
gamma[j,m]		0.17	0.12	0.11	0.09
gamma[j,u]		0.50	0.50	0.50	0.50
gamma[u,f]		0.50	0.38	0.31	0.39
gamma[u,m]		0.36	0.27	0.28	0.29
gamma[u,u]		0.25	0.15	0.13	0.15
f[a,f]		1.14			
f[a,m]		0.89			
f[a,u]		1.05			
f[j,f]		1.67			
f[j,m]		1.95			
f[j,u]		0.98			
f[u,f]		0.73			
f[u,m]		1.79			
f[u,u]		1.37			
beta[a,f]					0.47
beta[a,m]					0.56
beta[a,u]					0.35
beta[j,f]					0.55
beta[j,m]					0.89
beta[j,u]					-0.00
beta[u,f]					-0.02
beta[u,m]					0.64
beta[u,u]					1.36
sigma(log_lambda)	0.48	1.92	1.15	1.76	1.51
sigma(logit_phi)	0.38	0.60	0.50	0.87	0.80
sigma(logit_gamma)		3.16	1.14	1.13	7.49
sigma(log_f)		4.61			
sigma(beta)					9.69
